# Resolving the nanoparticles' structure-property relationships at the atomic level: a study of Pt-based electrocatalysts

**DOI:** 10.1016/j.isci.2021.102102

**Published:** 2021-01-28

**Authors:** Leonard Jean Moriau, Armin Hrnjić, Andraž Pavlišič, Ana Rebeka Kamšek, Urša Petek, Francisco Ruiz-Zepeda, Martin Šala, Luka Pavko, Vid Simon Šelih, Marjan Bele, Primož Jovanovič, Matija Gatalo, Nejc Hodnik

**Affiliations:** 1 Department of Materials Chemistry, National Institute of Chemistry, Hajdrihova 19, 1000 Ljubljana, Slovenia; 2 Department of Catalysis and Chemical Reaction Engineering, National Institute of Chemistry, Hajdrihova 19, 1000 Ljubljana, Slovenia; 3 Department of Analytical Chemistry, National Institute of Chemistry, Hajdrihova 19, 1000 Ljubljana, Slovenia

**Keywords:** electrochemistry, materials science, materials chemistry, materials characterization, energy materials

## Abstract

Achieving highly active and stable oxygen reduction reaction performance at low platinum-group-metal loadings remains one of the grand challenges in the proton-exchange membrane fuel cells community. Currently, state-of-the-art electrocatalysts are high-surface-area-carbon-supported nanoalloys of platinum with different transition metals (Cu, Ni, Fe, and Co). Despite years of focused research, the established structure-property relationships are not able to explain and predict the electrochemical performance and behavior of the real nanoparticulate systems. In the first part of this work, we reveal the complexity of commercially available platinum-based electrocatalysts and their electrochemical behavior. In the second part, we introduce a bottom-up approach where atomically resolved properties, structural changes, and strain analysis are recorded as well as analyzed on an individual nanoparticle before and after electrochemical conditions (e.g. high current density). Our methodology offers a new level of understanding of structure-stability relationships of practically viable nanoparticulate systems.

## Graphical Abstract

**Figure:**
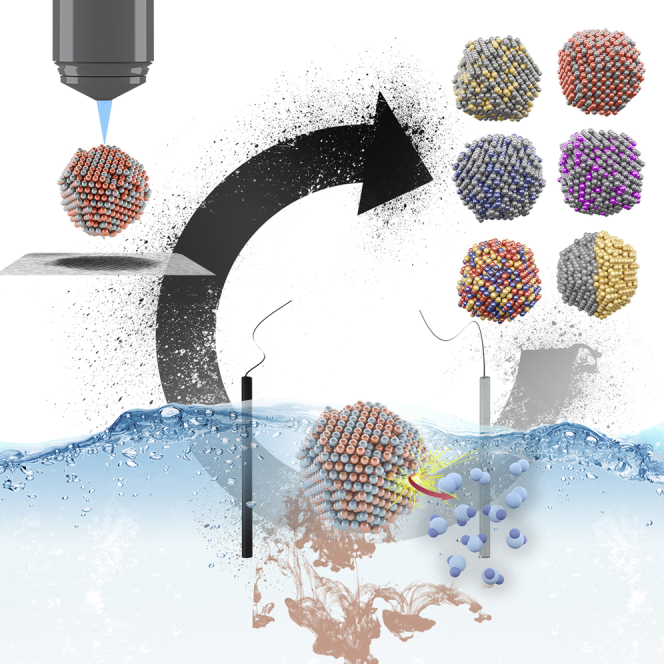

## Introduction

In the race toward cutting the World's greenhouse gas emissions, low-temperature proton-exchange membrane fuel cells (LT-PEMFCs) and batteries are needed to carry out electrochemical reactions to transform and store sustainably produced energy (Gröger et al., 2015). Although PEMFC technology is finally on the cusp of mass adoption, its high cost is becoming a major bottleneck—with Pt-based electrocatalyst contributing almost 50% of the total cost of PEMFC system manufacturing (DOE, 2017). The majority of these Pt-based electrocatalysts are necessary to catalyze the sluggish cathodic oxygen reduction reaction (ORR). This motivates optimizing the electrocatalyst price versus performance ratio using various approaches, with alloying of Pt with a less-expensive 3d transition metal (Pt-M; M = Cu, Ni, Fe, Co, etc.) being the closest to the production phase (Banham and Ye, 2017; Escudero-Escribano et al., 2018). Not only do Pt-M alloys enable better utilization of Pt by diluting the core of the particles with a more abundant alloying metal (Stamenković et al., 2007a) but they also substantially enhance the ORR intrinsic activity by influencing the Pt surface structure-electronic properties through the well-documented ligand and/or strain effect (Bu et al., 2016; Mezzavilla et al., 2016; Oezaslan et al., 2012a; Strasser et al., 2010). We note that ensemble effects are also at play (Greeley et al., 2002), however not as widely discussed in the Pt-alloy ORR community.

These performance improvements of Pt alloying, however, come with an inherent price, namely the fact that the alloying transition metals (M) are thermodynamically unstable (Pourbaix, 1974) and readily dissolve from the electrode under acidic conditions inside the PEMFCs. The dissolution of M can be slowed (but not completely stopped) by depleting the surface and near-surface region of Pt-M nanoparticles of M and thus forming a Pt-rich overlayer (Gatalo et al., 2019a). This is referred to as the activation process, where M is dissolved either chemically by acid washing (Gatalo et al., 2019a) or electrochemically (Gatalo et al., 2019b) by for example *in-situ* electrochemical cycling. However, although this initial dissolution is highly desired, further dissolution of M can diminish its positive ligand and/or strain effect and result in a loss of ORR performance (Hodnik et al., 2014; Jovanovič et al., 2016; Papadias et al., 2018). Furthermore, the dissolved M ions can also negatively affect the overall performance, especially in the membrane-electrode assembly (MEA)(Ahluwalia et al., 2018a; Braaten et al., 2019; Gasteiger et al., 2005; Mayrhofer et al., 2009). One such example is the interaction between the dissolved M ions and Pt surface that blocks the active surface and thus affects its electrochemical performance. In this sense, the most prominent example can be shown in the case of Pt-Cu alloy where Cu ions strongly interact, namely adsorb and reduce, on the Pt surface *via* well-known under-potential deposition (UPD); Cu can be found on both the cathode and the anode (Gatalo et al., 2019a, 2019b; Jia et al., 2013; Yu et al., 2012; Zhu et al., 2020). The second example is the replacement of protons in the ionomer with M cations, which results in higher O_2_ resistance as well as changes the water-uptake that consequently lowers the proton conductivity of the ionomer (Braaten et al., 2017, 2019). This causes ohmic losses in the cell as well as slows the ORR reaction rate, as fewer protons are available at the electrode (Kienitz et al., 2011; Okada et al., 2002). Lastly, any M present in the proton-exchange membrane will likely result in Fenton reactions and thus, its degradation that at the end leads to cell failure (Singh et al., 2018; Strlič et al., 2003). Consequently, despite the improvements in Pt utilization, intrinsic ORR activity, and overall electrocatalyst costs, the grand challenge of Pt-M electrocatalysts seems to be the long-term durability (Han et al., 2015). Therefore, if we wish to use Pt-M-containing electrocatalysts in PEMFCs, it is of paramount importance to understand, lower, and eventually eliminate leaching of M. In order to do this, it is crucial for the PEMFC community to first understand the related chemical and electrochemical phenomena on a fundamental level. Many extensive studies have already addressed the structure-property behavior of Pt-based electrocatalysts (Baldizzone et al., 2015a; Gatalo et al., 2018; Gong et al., 2020; Han et al., 2015; Li et al., 2014; Oezaslan et al., 2012b; Strasser and Kühl, 2016; Strasser et al., 2010; Yu et al., 2012; Zalitis et al., 2020). However, most of these studies are based on the oversimplified, perfectly shaped model systems, which are based on either model single-crystal measurements or a result of purely theoretical calculations, and thus, do not cover the necessary complexity of real nanoparticulate systems behind these relations.

**Figure 1:**
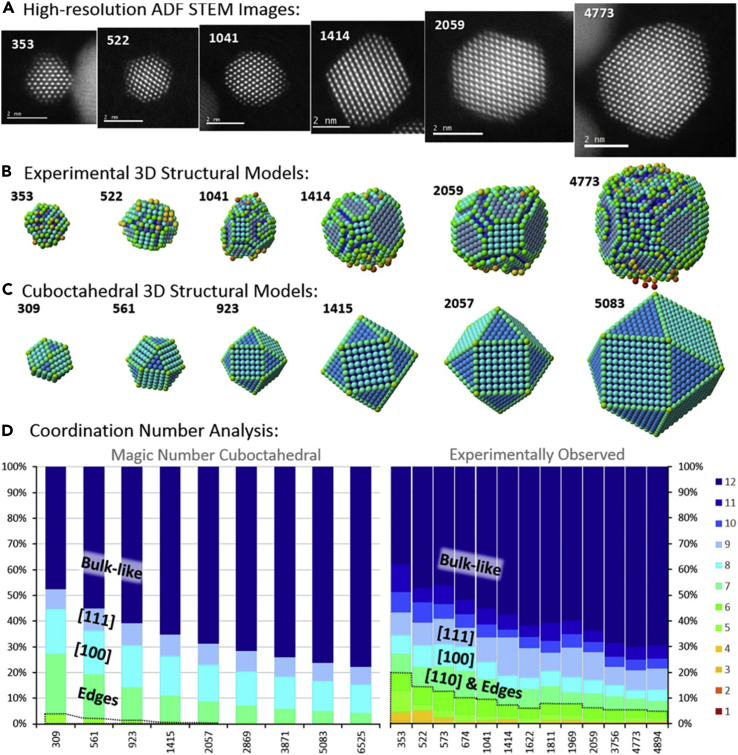
HR-STEM images, 3D structural models and coordination number analysis (A and B) (A) HR-STEM images and (B) accompanying hard-sphere models for experimental particles observed with atom counts near the magic numbers; models are colored by atom CNs (12) and rotated to show their dominant facets. (C) Magic number cuboctahedra are shown for comparison rotated to the same orientations. (D) Population histograms show the CN fractions as a function of particle atom-count for both magic (left) and experimentally observed particles (right). The 7, 8, and 9 coordination atoms correspond loosely to both (110) facets and edges between facets, whereas {100} and {111} surfaces, as well as the “bulk-like” atoms of ≥10 coordination, are labeled respectively. The dashed area represents the fraction of low-coordination adatom, corner, and step sites. The figure is adapted with permission from Ref (Aarons et al., 2017). Copyright (2017) American Chemical Society.

**Scheme 1:**
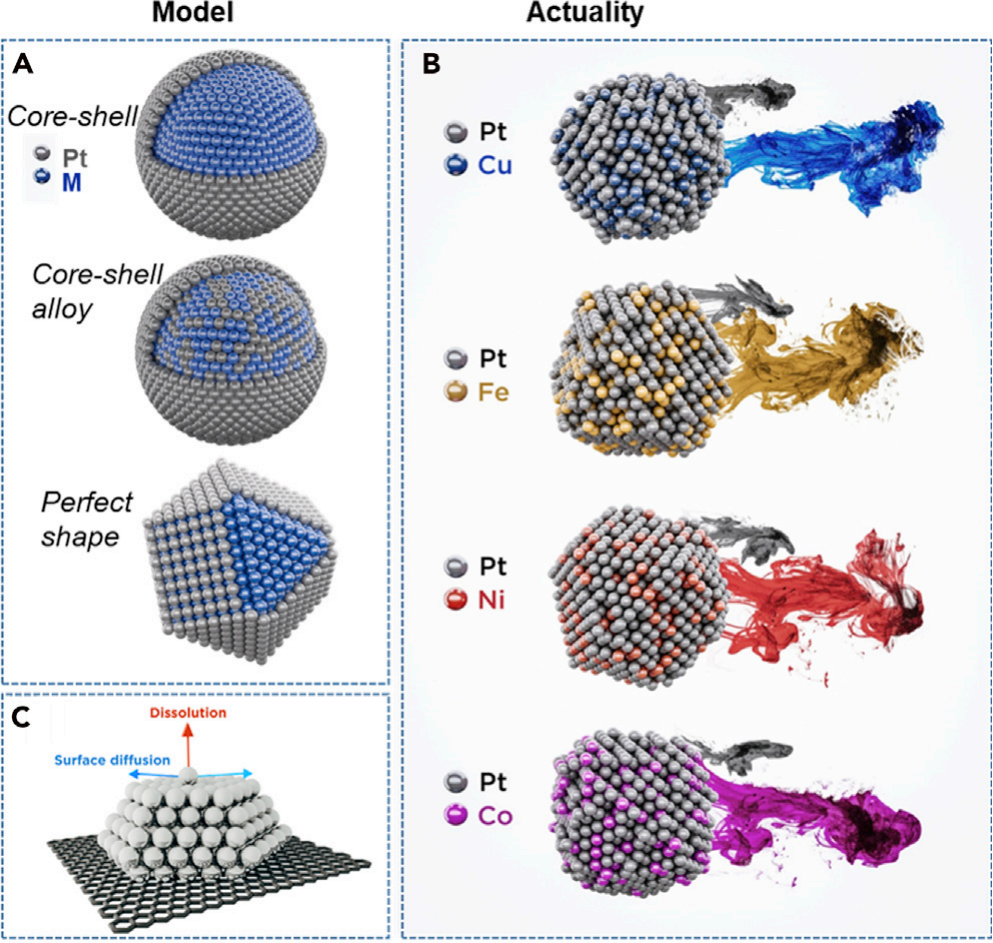
Model and actual structures of Pt-alloy nanoparticles (A) A scheme of an ideal model core-shell Pt-alloy systems. (B) A scheme of what most commonly a Pt-M electrocatalyst would look like, with many surface irregularities, kinks, steps, and real behavior in the PEMFC, namely their dissolution of Pt and M. The colors of M (Cu = blue, Fe = orange, Ni = red and Co = magenta) correspond to the ones used for Pt-M/C electrocatalysts in all the figures. (C) A schematic representation of specific surface atoms movement and/or removal.

**Figure 2:**
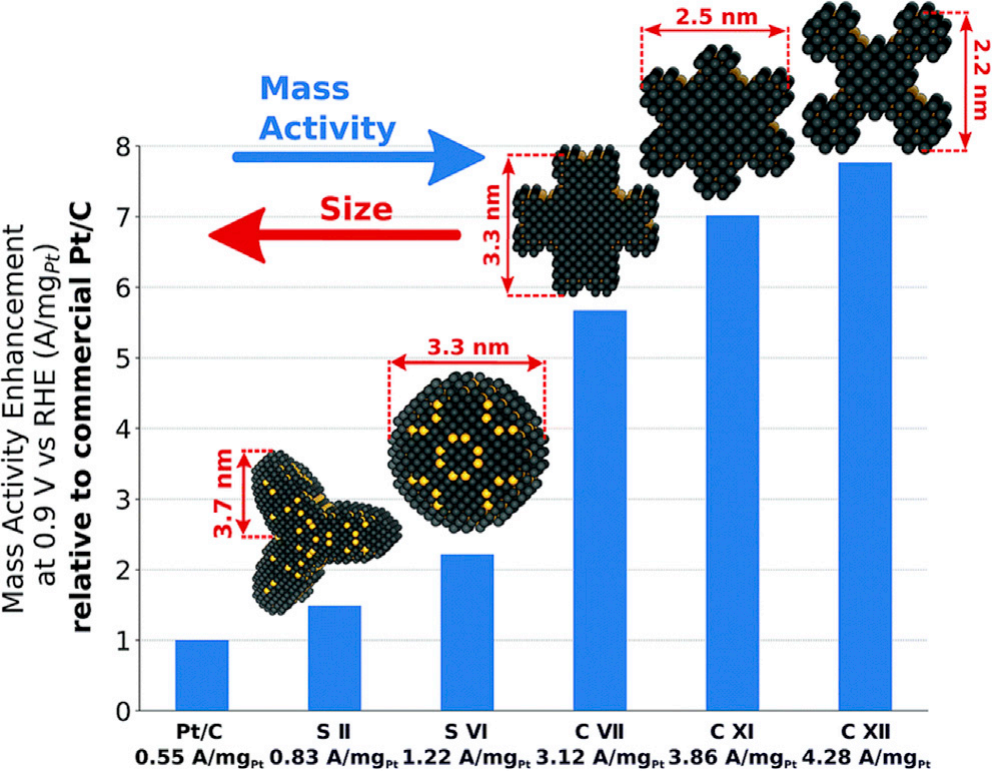
Mass activity enhancement of S II, S VI, C VII, C XI, and C XII over Tanaka commercial Pt/C electrocatalysts The figure is reproduced from Ref. (Rück et al., 2019) with permission from the Royal Society of Chemistry.

Upon looking at the structure-property of “real”’ electrocatalysts, the opportunity to improve the Pt-M electrocatalysts still lies in optimizing their structure so that the activity improvement caused by M is the highest while minimizing the adverse effects of the leached M ions and thus, possible issues related to durability. However, this task is tremendously complex. It is far from trivial to decide which structural feature to optimize in a given Pt-M system for the best overall performance, as real nanostructures are structurally far more diverse than the static model ones usually presented in the literature (for instance, pure Pt magic number cuboctahedra in Figure 1 and Pt-alloy model core-shell nanoparticles in Scheme 1A) (Aarons et al., 2017). Even for pure Pt surface, each atom has a certain coordination number (CN) that should affect its properties differently. More specifically, the parameter of generalized coordination number (gCN), which also takes the second sphere of neighbors into the account, was shown to directly correlate with ORR activity in the form of a volcano plot (Calle-Vallejo et al., 2015). Interestingly, it was recognized that the concave kink sites with gCN of 8.1 are optimal and that the ORR activity of Pt nanoparticles with the size of approximately 2–4 nm can be enhanced by up to almost eight times merely by changing their shape (Figure 2) (Rück et al., 2019). On the other hand, Aarons et al., demonstrated that “real” nanoparticles (from approximately 2 to 6 nm) contain a significantly different distribution of surface CNs with increased roughness (more defects) compared with the ideal cuboctahedral/truncated-octahedral nanoparticles (Figure 1). This results in more sites with lower CN and thus higher O-binding ability that reduces the ORR activity. In addition, they also observed that the amounts of active sites, with the optimal ∗OH-binding energy of 0.15–0.2 eV weaker than that of the Pt{111} surface (that is gCN is approximately 8.1 compared with 7.5 on Pt{111}), can vary for more than a factor of two at Pt particles with similar sizes.

When it comes to Pt nanoalloys, the degree of complexity that influences their activity and stability *via* the well-accepted structure-property relationships (Yang et al., 2017) becomes even higher. Some examples of parameters increasing the complexity are chemical composition of the alloy (Gatalo et al., 2019c; Mezzavilla et al., 2016; Oezaslan et al., 2012a; Zalitis et al., 2020), degree of order in the crystal structure (intermetallic chemical order/disorder phases) (Xiong et al., 2019), the strain of surface Pt induced by, for instance, the thickness of Pt-shell, amount of retained less noble metal (Strasser et al., 2010) etc. Many of them are interconnected and thus challenging to control. Both extreme sensitivity and the complexity of Pt ORR electrocatalysts are directly addressed and elaborated in the recent viewpoint of Chattot et al. (Chattot et al., 2020). In addition, besides the detailed atomic structure, which contains defects such as steps, adatoms, twinning, and dislocations, we must also consider the fact that the surface is not stagnant with the time under operation. Therefore, the structure and thus property of each individual particle, which is far from the idealized models of spherical or cuboctahedral or truncated-octahedral core-shell particles (Scheme 1A), is also dynamically changing. For this reason, dynamic alternations of nanostructures should be recognized as a structure-stability parameter, namely atomic movement and/or dissolution (Scheme 1B). Looking at merely one TEM snapshot of nanoparticles atomic structure does not necessarily provide the only structure that induces certain function and also not a realistic idea of if and how specific atoms move or get dissolved (Scheme 1C).

On the other hand, when looking at the Pt-M electrocatalysts from the synthesis point of view, the most crucial step toward optimizing the structure and behavior of an electrocatalyst is to be able to tune its structural properties. Indeed, colloidal chemistry recipes can produce nanoparticles with controlled shape by governing its growth *via* capping agents (Ahmadi et al., 1996; Neumann et al., 2017; Quinson et al., 2018; Speder et al., 2016; Xia et al., 2015). However, besides issues related to the removal of this stabilizing molecules from the particles surface, it is a fact that it is practically impossible to synthesize a perfect model system that contains millions of ideal core-shell (spherical or cuboctahedra) nanoparticles with identical atomic structures, such as the one shown in Figure 1C and Scheme 1A.

A major challenge for Pt-M/C electrocatalysts is, besides shape, also optimizing the high-temperature thermal annealing treatments for each individual alloy to achieve the desired alloy phase (as defined in the phase diagrams). In addition, each less-noble metal (as well as the Pt alloy itself) can also interact with the carbon support during annealing, as defined in the M-C phase diagrams, which further complicates the synthesis by forming thin, sometimes graphitized, carbon shells on the surface of the particles, depending on the nature of metal or alloy type (Baldizzone et al., 2014; Chong et al., 2018; Mezzavilla et al., 2016; Wang et al., 2019a; Xiao et al., 2019).

An additional step that is necessary for alloy-based electrocatalysts is activation, *i.e.* exposing the as-synthesized/thermally annealed material to appropriately corrosive chemical or electrochemical conditions (e.g. potential cycling activation; PCA (Gatalo et al., 2019b)) in order to remove M from the surface and near-surface region and thus reveal Pt active surface, namely Pt-shell. The dealloying or leaching electrochemical phenomenon, of course, changes the structure of the nanoparticles depending on the initial shape, composition, crystal phase of the native alloy, and conditions (Pavlišič et al., 2016). This process was shown to create some very active structures, referred to as stressed sites or surface distortion with the local disorder and straining (Chattot et al., 2018; Li et al., 2016). At this point, one can ask an intriguing question whether the function of alloying Pt with unstable 3D metals is to create the superiorly active stressed sites with optimal gCNs (merely the atomic arrangement of Pt atoms in pure Pt nanostructures, as portrayed in Figures 1 and 2) or is it to alter Pt surface adsorption properties from underneath *via* strain and/or ligand effects (metal atoms below Pt surface in Pt-alloy nanostructures, as in Scheme 1A).

Overall, each Pt-M combination is uniquely defined by its properties and thus with its own set of constraints. Therefore, one must understand their differences, which is again a very complex task. These range from the synthesis (Gatalo et al., 2019c), alloys phase diagrams, metals mixing behavior (Dean et al., 2020), M-specific nanoalloys anisotropic inhomogeneity (Cui et al., 2013; Gan et al., 2014; Ruiz-Zepeda et al., 2019), standard redox potentials and/or Pourbaix diagrams of the less-noble metal, interaction of dissolved M ions from the electrolyte with Pt surfaces such as UPD (Gatalo et al., 2019b), pre-treatment (Baldizzone et al., 2015b; Gatalo et al., 2019a), general stability-dissolution-corrosion behavior (Gatalo et al., 2019b), and behavior in the PEMFC three-phase boundary environment (Ahluwalia et al., 2018a; Yu et al., 2012). Lastly, there is currently no reliable database of as-synthesized shapes and their expected shape transformations (1) upon exposure to high temperatures of thermal annealing processes (Gan et al., 2016) and/or (2) upon activation (de-alloying) (Ruiz-Zepeda et al., 2019), much less a database that could take into the account such a wide range of before-mentioned parameters.

**Scheme 2:**
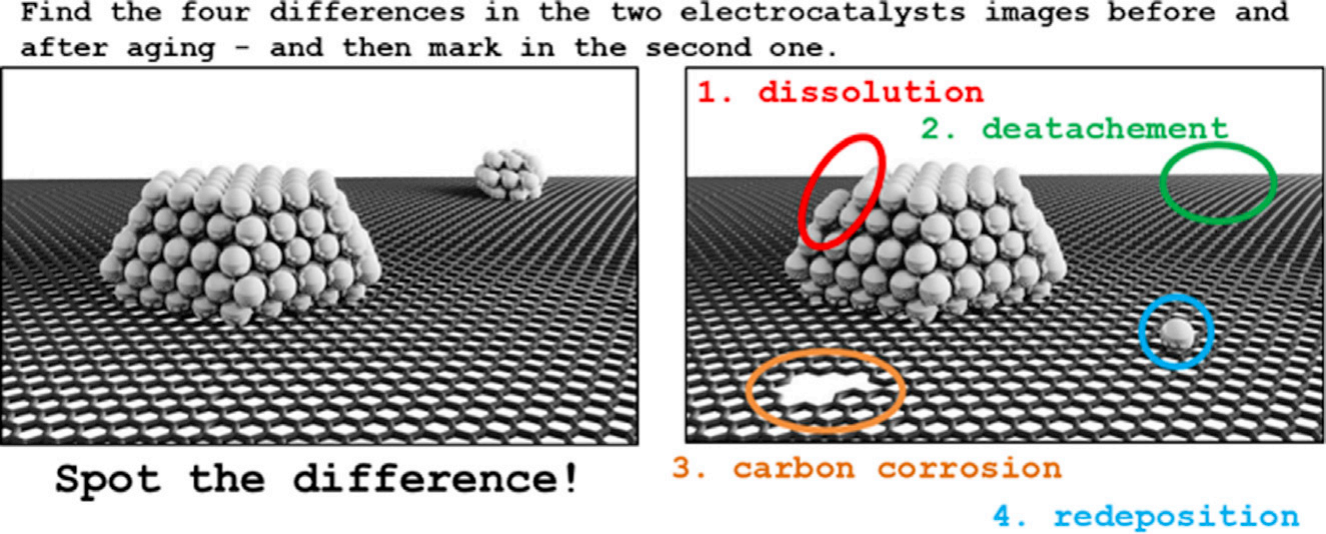
A scheme of “Spot the difference approach” in electron microscopy The figure is adapted with permission from Ref. (Hodnik and Cherevko, 2019). Copyright (2019) Elsevier.

As part of this work, we have tried to observe this structural complexity by investigating in total eight different commercially available Pt-based electrocatalysts, namely (1) four Pt/C electrocatalysts (from Umicore, TKK, and JM) and (2) four Pt-M/C electrocatalysts (M = Cu, Fe, Ni, Co; PK Catalyst; purchased at Fuel Cell Store). In the first part, these electrocatalysts are initially systematically investigated using a rather conventional top-down approach with the purpose of exposing the differences of the seemingly comparable systems (Scheme 1). Our structural investigation of these commercial electrocatalysts reveals a far more complex picture, especially when compared with the ideal model systems. The results suggest that it is practically impossible to control the synthesis of Pt-based nanoparticles in a way that would enable to fundamentally study and understand the structure-property behaviors as well as achieve major breakthroughs using a conventional top-down approach of studying such systems. As a solution, we in the second part introduce a bottom-up approach by “playing the game” of “Spot the difference” (Scheme 2) (Hodnik and Cherevko, 2019) where an individual nanoparticle is studied at the atomic level before and after an electrochemical treatment. By understanding the history of this specific nanoparticle and its changes at the atomic level, we gain indisputable evidence on some of the fundamental electrochemical phenomena and thus reliable structure-property relations. This is, in our opinion, the only viable approach to study the structure-property relationships of Pt-based nanostructures.

In order to provide a proper comparison between both approaches (top-down and bottom-up), physical, chemical, and electrochemical properties were systematically characterized with a combination of classical characterization techniques. These include X-ray diffraction (XRD), *ex-situ* transmission electron microscopy (TEM), thin-film rotating disc electrode (TF-RDE), as well as novel advanced characterization techniques such as highly sensitive online measurements of electrochemically dissolved metals (Pt and M; using electrochemical flow cell coupled to an inductively coupled plasma mass spectrometer; EFC-ICP-MS (Gatalo et al., 2016, 2019a, 2019b; Jovanovič et al., 2016, 2017a)) and detection of volatile compounds by direct coupling of an electrochemical cell to a mass spectrometer (EC-MS), which enables studying carbon corrosion *via* CO_2_ detection. In addition, a newly developed modified floating electrode (MFE) methodology was used to enable identical location transmission electron microscopy (IL-TEM) with atomic resolution.

## Results and discussion

### Top-down approach of studying the structural complexity of Pt and Pt-alloy nanoparticles on high-surface-area carbons

#### Structural analysis of the “as-purchased” Pt-based electrocatalysts

**Figure 3:**
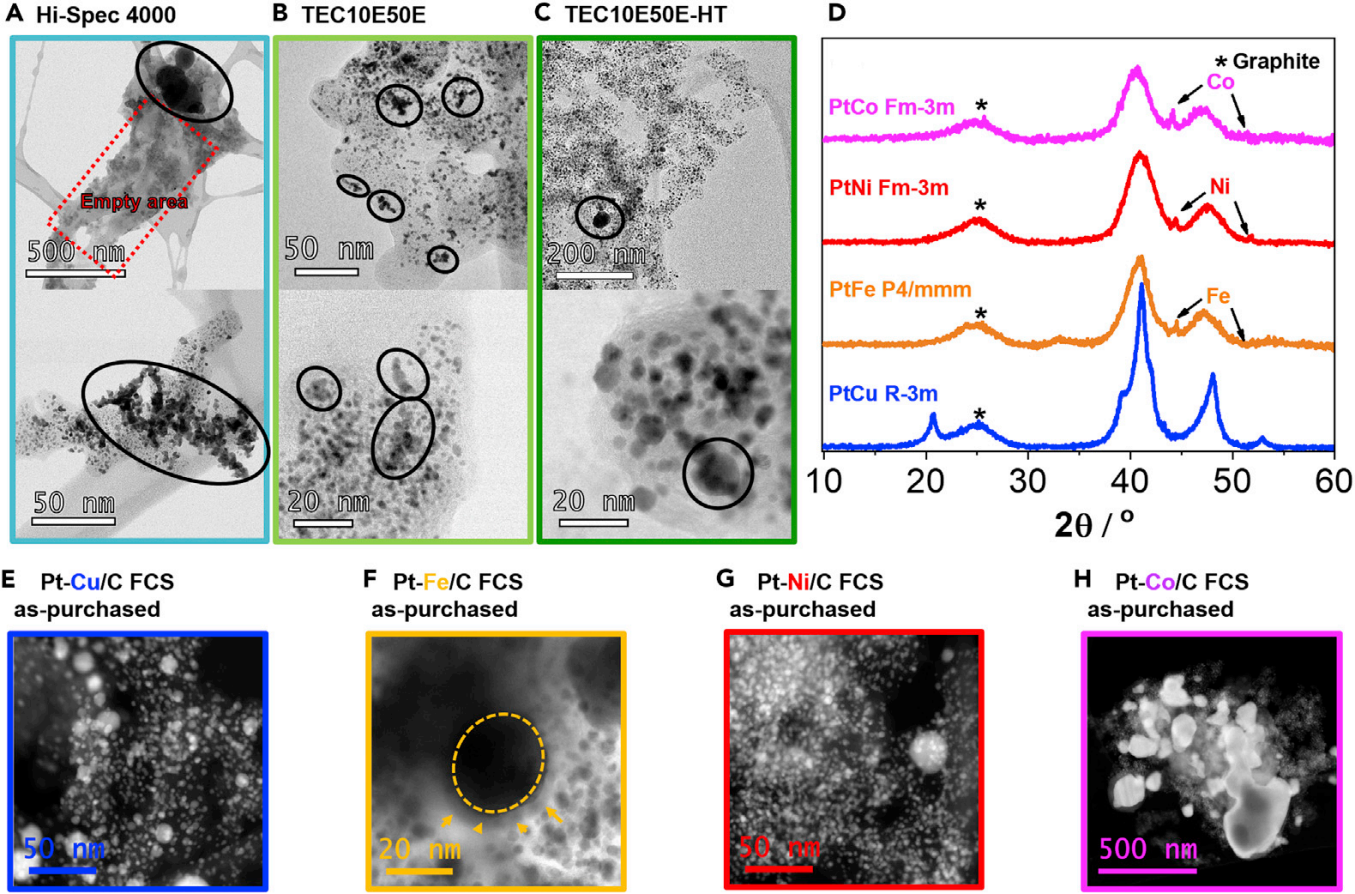
TEM and XRD analysis of commercially available Pt-based electrocatalysts (A–C) TEM analysis of commercially available Pt/C electrocatalysts (Hi-spec 4000 from JM—teal, TEC10E50E from TKK—light green and TEC10E50E-HT from TKK—green; see also Supplemental information, Figure S1 and Table S1 for TF-RDE analysis). (D) XRD analysis of as-purchased Pt-M/C electrocatalysts from FCS. (E–H) STEM analysis of all four Pt-M/C electrocatalysts in the as-purchased state. See also Supplemental information, Figures S2–S9 for EDX analysis. The colors (Pt-Cu = blue, Pt-Fe = orange, Pt-Ni = red and Pt-Co = magenta) correspond to the ones used for the graphs and borders in all of the figures.

**Pt/C**: Figures 3A–3C show TEM analysis of the investigated Pt/C references. What is common with all of them is that these electrocatalysts are all far from perfect with evident cases of big(ger) particles, metal-free areas on carbon support, as well as smaller or larger agglomerates. These imperfections are all a consequence of the currently used industrial production methods (Matsutani et al., 2010) used to deposit Pt nanoparticles on carbon supports at high Pt loadings (>40 wt%) (Taylor et al., 2016). As shown by our TF-RDE analysis, the electrocatalysts also vary in their electrochemical performance (see Supplemental information, Figure S1 for ORR polarization curves and CO-electrooxidation CVs as well as Table S1 for the TF-RDE data). For non-thermally annealed Pt/C electrocatalysts (Hi-Spec 4000 and TEC10E50E), their specific activity (SA) seems to be within the range of error (~0.6 mA cm^−2^_Pt_), whereas the main difference between the two is a much higher ECSA normalized using CO electrooxidation (ECSA_CO_; ~80 m^2^ g^−1^_Pt_ for TEC10E50E in comparison to ~53 m^2^ g^−1^_Pt_ for Hi-Spec 4000). This could be either due to slight differences in the used production methods or due to differences in the available surface area (BET) of carbon support. Although Hi-Spec 4000 is supported on Vulcan XC-72 (BET = 250 m^2^ g^−1^), TEC10E50E is supported on Ketjen Black EC300J (BET = 800 m^2^ g^−1^) with a much higher surface area. On the other hand, when comparing TF-RDE characterization of thermally annealed Pt/C electrocatalysts (TEC10E50E-HT and Elyst Pt50 0550) we notice the similarity between the two both in terms of SA (~0.4 mA cm^−2^_Pt_) and ECSA (~50 m^2^ g^−1^_Pt_ for both). Both electrocatalysts also use Ketjen Black EC300J as the carbon support. Although all electrocatalysts have a high enough ECSA (above 40 m^2^ g^−1^_Pt_ (Kongkanand and Mathias, 2016)) to perform well in an MEA, better initial dispersion of Pt over carbon allows for retaining a higher ECSA after thermal annealing (Gatalo et al., 2019c). The thermal annealing is immensely important in terms of electrocatalyst stability because of better resistance to Pt dissolution due to larger average particle size, crystallinity, and improved stability of the carbon support (Jovanovič et al., 2014; Maselj et al., 2020; Matsutani et al., 2010). For practical use at the MEA level, the authors recommend the PEMFC scientific community to use TEC10E50E-HT or Elyst Pt50 0550 as a benchmark cathode and/or anode Pt/C electrocatalyst (or similar Pt-Co analogues available at TKK/Umicore). This is because the latter offers a good compromise between both kinetic and high-current density performance as well as stability (Padgett et al., 2019). However, it is also important to note that the electrocatalysts commercially available to the scientific community might differ from their state-of-the-art analogues used in commercial MEAs.

**Pt-M/C**: In order to reveal the complexity behind already merely variation in M (M = Cu, Fe, Ni, or Co), we have compared four Pt-M/C electrocatalysts purchased at Fuel Cell Store (FCS) (produced by PK catalyst). Based on the product description (20% Platinum M (1:1 ratio) supported on Vulcan XC-72 (carbon) with an average particle size of 2–3 nm (Author Anonmous, 2020a), we have expected the type of M (Cu, Fe, Ni or Co) to be the only major difference. Although ICP-OES digestion results of electrocatalysts in the as-purchased state confirmed the stated 1:1 atomic ratio of Pt to M and a total metal loading (Pt + M) of approximately 20 wt% (see Supplemental information, Table S2), X-ray diffraction (XRD) spectra (Figure 3D) and TEM analysis (Figures 3E–3H) reveal many features that substantially differentiate the as-purchased Pt-Co/C FCS electrocatalysts. The first parameter that differentiates all four Pt-M/C electrocatalysts is their crystal phase. Although XRD spectra of Pt-Co/C (Ref. code 03-065-8968) and Pt-Ni/C (Ref. code 03-065-9445) electrocatalysts show the same disordered Fm-3m crystal structure, the nanoparticles in Pt-Fe/C and Pt-Cu/C exhibit ordered intermetallic P4/mmm (Ref. code 03-065-9121) and R3-M (Ref. code 00-042-1326) phases, respectively (Figure 3D). Furthermore, although the producer has not revealed their exact thermal annealing protocol, we anticipate that the observed crystal structures of the alloy nanoparticles would anyway differ even at the same thermal treatments due to unique phase diagrams of analyzed Pt-M combinations. In the literature, intermetallic alloys have already caught much attention due to their promising ORR performance (Gamler et al., 2018; Hodnik et al., 2014; Li and Sun, 2019; Pavlišič et al., 2016; Rößner and Armbrüster, 2019; Zhang et al., 2020). However, although we can find many reports on intermetallic alloys of Pt-Cu (Bele et al., 2014; Gatalo et al, 2019a, 2019b, 2019c, 2019d; Hodnik et al., 2012a, 2014; Pavlišič et al., 2016) and Pt-Fe (Liu et al., 2019; Wang et al., 2019b; Wittig et al., 2017), the reports on intermetallic phases of more industrially relevant Pt-Co (Cui et al., 2020; Xiong et al., 2019) and even more so Pt-Ni alloys (Lu et al., 2009) are still very scarce (Leonard et al., 2011; Zou et al., 2015). Careful examination of the XRD spectra also reveals the presence of pure M phases in Pt-M/C electrocatalysts containing Fe, Ni, and Co (Figure 3D; as pointed by the arrows). Although the relative intensity of peaks for pure M is relatively low (which usually suggests that only a small amount of pure M phase is present), the peaks are also narrow—pointing toward the presence of larger aggregates of M. In that sense, STEM analysis (Figures 3E–3H) provides us with some clues; although it is already well known that M particles can get encapsulated in layers of graphitic carbon (Baldizzone et al., 2014; Chong et al., 2018; Mezzavilla et al., 2016; Wang et al., 2019a), this is not often connected to the thermal annealing of Pt-M electrocatalysts. Although some M during thermal annealing usually always ends up alloying with Pt, a well-known phenomenon (especially in synthesis of graphene (Li et al., 2009)) is the solubility of carbon in M phase at high temperatures. Upon cooling, it results in surface segregation of carbon in the form of a graphitized carbon shell as exemplified in Figure 3F. The absence of pure Cu phase is, however, expected, given that the solubility of carbon in Cu compared with other metals is several orders of magnitudes lower (Li et al., 2009). How efficiently the alloying metal M is incorporated into the Pt alloy and how much of it remains un-alloyed is a crucial parameter to consider when designing the synthetic protocol for different Pt-M alloy nanoparticles. Not only is the Pt:M ratio in the alloyed particles more difficult to control if a pure M phase is also formed but the pure M particles present a potential danger if the graphitic shell is punctured *via* mechanical force or electrochemical oxidation. Especially when one considers the integration of the electrocatalyst into the MEA, the pure M phase needs to be removed beforehand (for example, by chemical activation (Baldizzone et al., 2015b)).

The presence of pure M particles means that the Pt-M nanoparticles are in-fact more Pt rich than as stated by the producer (Author Anonmous, 2020a) and confirmed by ICP-OES (1:1 atomic ratio, as shown in Supplemental information, Table S2). The inhomogeneity of compositional distribution of metals in the as-purchased Pt-M/C electrocatalysts was additionally analyzed using EDX in STEM. Two types of sample areas were selected, namely those where only small (2–3 nm) Pt-M particles were observed and those where also larger particles were present. The areas containing predominantly small nanoparticles show approximately 60–65 at% Pt (as exemplified in Supplemental information, Figures S2–S5), which is higher than the approximately 50 at% values obtained with ICP-OES analysis. However, areas that include larger particles (above 10 nm), on the other hand, consistently show above 50 at% (and up to as much as 97 at%) of M (as exemplified in Supplemental information, Figures S6–S9).

#### Electrocatalytic performance and structural changes of Pt-M electrocatalysts after PCA and after ADT

**Figure 4:**
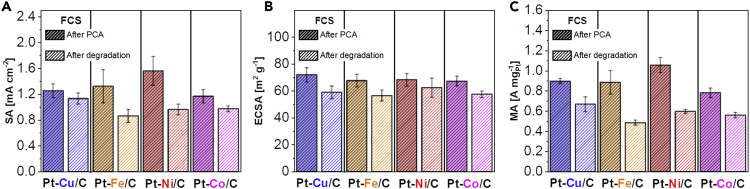
SA, ECSA and MA comparison of Pt-M/C FCS electrocatalysts after PCA and after ADT Comparison of (A) specific activity (SA at 0.9 V_RHE_), (B) ECSA_CO_ and (C) mass activity (MA at 0.9 V_RHE_) of Pt-M/C electrocatalysts evaluated both after PCA (200 cycles in 0.1 M HClO_4_, 0.05–1.2 V_RHE_, 300 mV s^−1^, Ar saturated, 600 rpm) and degradation (ADT; 5000 cycles in 0.1 M HClO_4_, 0.4–1.2 V_RHE_, 1 V s^−1^, Ar saturated, 600 rpm). See also Supplemental informationFigures S18–S20 for ORR polarization curves, CO electrooxidation experiments and calculated Tafel plots that compare the activity after PCA and after ADT. The colors (Pt-Cu = blue, Pt-Fe = orange, Pt-Ni = red and Pt-Co = magenta) correspond to the ones used for the graphs and borders in all of the figures.

Figures 4A–4C compare SA, ECSA_CO_, and MA for all four Pt-M/C (FCS) electrocatalysts using TF-RDE (1) after PCA (200 cycles in 0.1 M HClO_4_, 0.05–1.2 V_RHE_, 300 mV s^−1^, Ar saturated, 600 rpm), as well as (2) after an accelerated degradation test (ADT; 5000 cycles in 0.1 M HClO_4_, 0.4–1.2 V_RHE_, 1 V s^−1^, Ar saturated, 600 rpm). Although the activity of Pt-Cu/C, Pt-Fe/C, and Pt-Co/C is within the margin of error (around 1.1.–1.2 mA cm^−2^_Pt_, within a margin of error deduced by 3–5 repetitions for each electrocatalyst), Pt-Ni/C clearly exhibits the highest SA after PCA reaching 1.6 mA cm^−2^_Pt_ (Figure 4A). Due to similar average particle sizes of 2–3 nm reported by the producer (PK Catalyst; FCS), ECSA_CO_ values were all within the margin of error (Figure 4B). Consequently, MAs follow the trends of SAs (Figure 4C), namely around 0.8–0.9 A mg^−1^_Pt_ and 1.1 A mg^−1^_Pt_ for Pt-Ni/C (Figure 4C). After performing the ADT, the highest SA was exhibited by the Pt-Cu/C analogue, namely around 1.1 mA cm^−2^_Pt_ (Figure 4A). SA for the other three materials was, on the other hand, lower and comparable, around 0.8–0.9 mA cm^−2^_Pt_ (Figure 4A). The most noticeable feature here is that relative SA losses exhibited by Pt-Ni/C and Pt-Fe/C analogues were much larger in contrast to the Pt-Cu/C and Pt-Co/C analogues. On the other hand, because ECSA_CO_ losses between the samples are rather similar for all four Pt-M/C electrocatalysts (Figure 4B), consequently, MAs again follow the trends of SAs (Figure 4C), namely around 0.5–0.6 A mg^−1^_Pt_ for all four Pt-M/C electrocatalysts.

**Figure 5:**
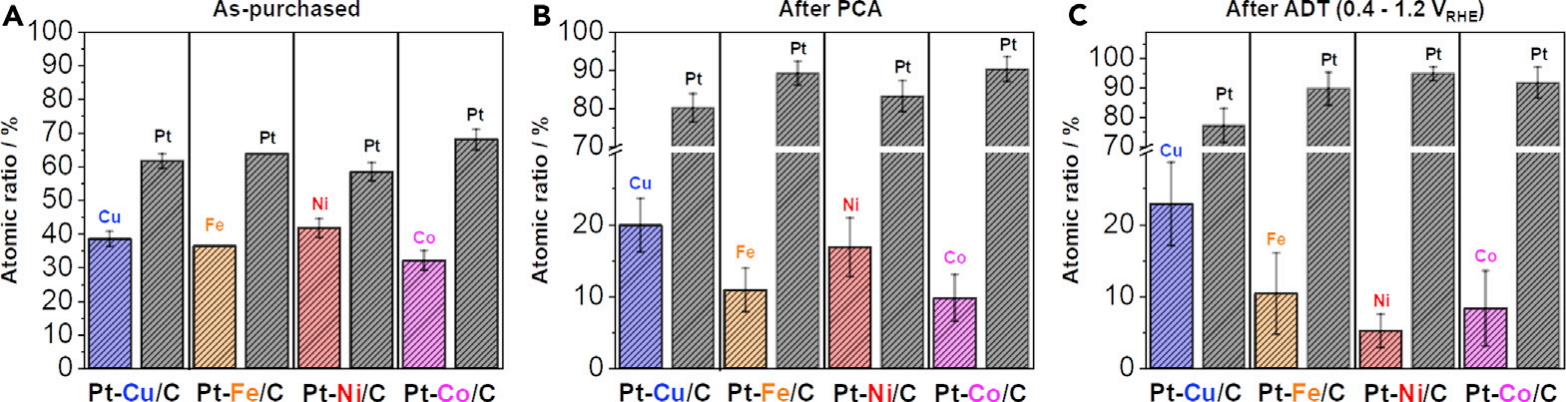
*ex-situ* EDX-STEM comparison of Pt-M/C FCS electrocatalysts after PCA and after ADT *Ex-situ* EDX-STEM analysis of Pt-M/C electrocatalysts (B) after PCA (200 cycles in 0.1 M HClO_4_, 0.05–1.2 V_RHE_, 300 mV s^−1^, Ar saturated, 600 rpm) and (C) after ADT (5000 cycles in 0.1 M HClO_4_, 0.4–1.2 V_RHE_, 1 V s^−1^, Ar saturated, 600 rpm). A minimum of four large areas containing several hundred nanoparticles were measured each time. For more EDX-STEM of Pt-M/C electrocatalysts see also Supplemental informationFiguresS2–S9. The colors in the bar graphs (Pt = black, Cu = blue, Fe = orange, Ni = red and Co = magenta) correspond to the atomic percentage ratios.

This activity decrease after the degradation protocol in Pt-alloy catalysts is usually attributed to the loss of M and, thus, the loss of its positive effect on Pt-M activity (ligand/strain effect) (Mezzavilla et al., 2016). However, without the exact atomically resolved structure and compositional information of each nanoparticle, it is misleading to flawlessly interpret these results comprehensively. As shown in XRD and *ex-situ* STEM analysis in Figures 3D–3H, each investigated Pt-M/C electrocatalyst is different enough that it is practically impossible to attribute the differences to any single structural feature. In order to gain a better understanding, we have additionally used *ex-situ* EDX-STEM analysis to compare chemical compositions of all four Pt-M/C electrocatalysts in the as-purchased state (Figure 5A), after PCA (Figure 5B) as well as after ADT (Figure 5C). However, the analysis of as-purchased Pt-M/C electrocatalysts reveals that even when providing a statistically significant pool of several hundreds of nanoparticles (analyzing many different areas), the use of *ex-situ* EDX-STEM is rather questionable due to previously discussed local in-homogeneities (Figures 3E–3H). As shown in Figure 5A, by avoiding areas with pure M phases or very M-rich phases, the general chemical composition is much more Pt rich than the 1:1 ratio reported by the producer and what we measured using ICP-OES. However, for the interpretation of electrochemical activity, it is the de-alloyed structure (after PCA) that should be considered and not as-synthesized one (as-purchased state). De-alloying of M from the Pt-M nanoparticles leads to two typical structural changes, namely formation of a core-shell structure (Pt-rich overlayer with a Pt-M core (Gatalo et al., 2019b; 2019d; Ruiz-Zepeda et al., 2019)) and/or porosity (Hodnik et al., 2012b). De-alloying of Pt-M nanoparticles forms pores above a certain critical size, critical Pt-M composition ratio, and critical conditions. Below these critical parameters, however, core-shell structures are expected (Hodnik et al., 2012b). Regardless of the structural changes, the hypothesis for comparing the chemical compositions after PCA and after ADT was that the highest loss of M after ADT should correlate to the highest loss of ligand/strain effect and thus, the highest loss of SA (Figure 4A). However, although we have in-fact observed a big loss of Ni in the case of Pt-Ni/C electrocatalysts, which indeed experienced the highest loss of SA, the same explanation cannot be used for Pt-Fe/C and Pt-Cu/C electrocatalysts. In both cases, the loss of M after ADT was insignificant (in the case of Pt-Cu/C even in the margin of error); however, in both cases, a loss of SA was observed with Pt-Fe/C losing a rather significant amount. This is another indication that the structure-stability relationship of Pt-based electrocatalysts is significantly more complex than what a simple one-parameter correlation can explain.

**Figure 6:**
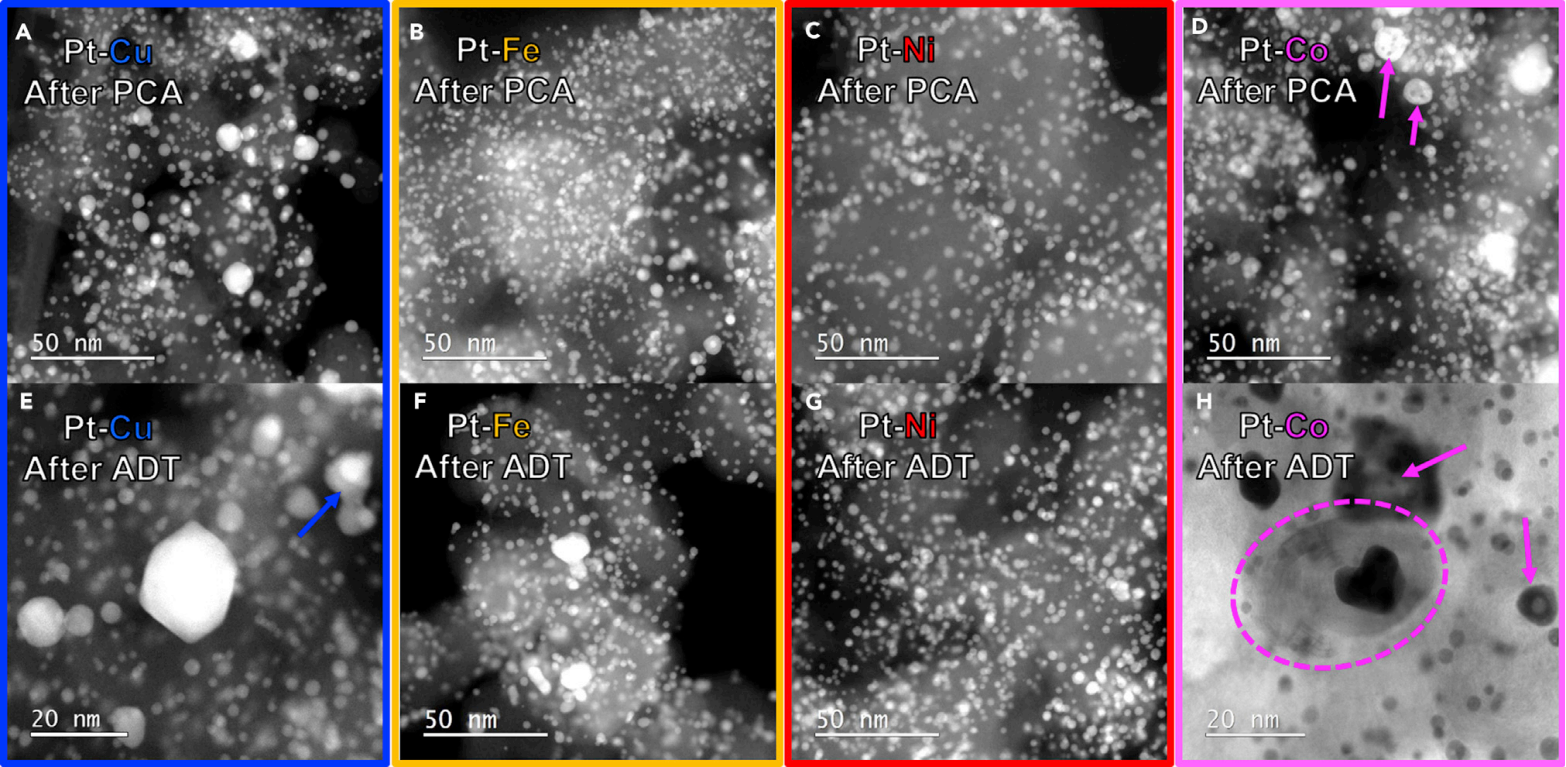
*ex-situ* STEM comparison of Pt-M/C FCS electrocatalysts after PCA and after ADT STEM analysis of all four Pt-M/C electrocatalysts (A–D) after PCA (600 rpm, Ar saturated, 200 cycles in 0.1 M HClO_4_, 0.05–1.2 V_RHE_, 300 mV s^−1^) and (E–H) after ADT (600 rpm rotation, Ar saturated, 5000 cycles in 0.1 M HClO_4_, 0.4–1.2 V_RHE_, 1 V s^−1^). Additional STEM BF and HAADF analyses are available in Supplemental information, Figures S10–S17. The colors (Pt-Cu = blue, Pt-Fe = orange, Pt-Ni = red and Pt-Co = magenta) correspond to the ones used for the graphs and borders in all of the figures.

Further *ex-situ* STEM analysis of structural changes of all four Pt-M/C electrocatalysts after PCA (Figures 6A–6D; see also Supplemental information Figures S10–S13) reveals even more differences. For example, the Pt-Cu/C electrocatalyst analyzed after PCA in Figure 6A (see also Supplemental information Figure S10 for more STEM images) shows a mix of smaller (~2–10 nm) and larges nanoparticles (10 + nm). On the other hand, Pt-Fe/C and Pt-Ni/C electrocatalysts analyzed after PCA in Figures 6B and 6C, respectively, (see also Supplemental information Figures S11 and S12 for more STEM images) show a much more uniform size distribution (in other words, without any >10 nm particles as in the case of Pt-Cu/C). Lastly, Pt-Co/C electrocatalyst analyzed after PCA in Figure 6D (see also Supplemental information Figure S13 for more STEM images) shows a mix of small and large nanoparticles as in the case of Pt-Cu/C. However, in addition, the Pt-Co/C electrocatalyst also exhibits nanoporosity. These differences after electrochemical treatment are noticed despite a very similar chemical composition of the as-purchased Pt-M/C electrocatalysts, which was measured by *ex-situ* EDX-STEM (Figure 5A). The presence of porosity in the case of Pt-Co/C electrocatalyst already after PCA might explain the lower amount of Co determined using *ex-situ* EDX-STEM after PCA in contrast to the other three Pt-M/C electrocatalysts. On the other hand, the absence of porosity in the presented Pt-Cu/C electrocatalyst may initially appear to contradict some of the previous studies on Pm-3m Pt-Cu crystal structures with the Pt:Cu ratio of 1:3 (Gatalo et al., 2016; Hodnik et al., 2014; Jeyabharathi et al., 2013; Pavlišič et al., 2016; Ruiz-Zepeda et al., 2019). We note, however, that there are additional parameters that determine if porosity formation will occur or not, namely a critical size (larger then approximately 30 nm) and a critical Pt-M composition ratio (Hodnik et al., 2012b). The type of M also influences which size and/or composition thresholds are critical (Oezaslan et al., 2012b). Thus, in the case of the 1:1 Pt-Co Fm-3m crystal structure, it seems that these critical parameters that result in porosity occur at a lower particle size and a more Pt-rich composition than in the case of 1:1 Pt-Cu R-3m crystal structure. The best way of avoiding porosity, however, is by keeping the particle size distribution small (below 5 nm) and uniform as observed for Pt-Fe/C and Pt-Co/C electrocatalysts.

After ADT (Figures 6E–6H; see also Supplemental information Figures S14–S17), however, *ex-situ* STEM analysis of structural changes reveals even more. For example, Pt-Cu/C electrocatalyst analyzed after ADT in Figure 6E (see also Supplemental information Figure S14 for more STEM images) again does not seem to show any evidence of porosity formation. This supports the *ex-situ* EDX-STEM compositional analysis where the Pt:Cu ratio stayed within the margin of error (Figures 5B and 5C). In addition to already previously observed (after PCA; Figure 6A) mix of smaller and larger particles, we also observe necking and thus, agglomeration. This explains the ECSA_CO_ losses observed in the TF-RDE comparison after PCA and ADT (Figure 4B; see also Supplemental information Figure S19A for comparison of CO-electrooxidation CVs). As in the case of Pt-Cu/C electrocatalyst, we have not observed any porosity formation in the case of Pt-Fe/C and Pt-Ni/C electrocatalysts as shown in Figures 6F and 6G (see also Supplemental information Figures S15 and S16 for more STEM images). This is in line with the initially more uniform size distribution observed after PCA in Figures 6B and 6C (see also Supplemental information Figures S11 and S12 for more STEM images). However, although Pt-Ni has lost the most Ni upon ADT (Figures 5B and 5C), its ECSA_CO_ loss (Figure 4B) is the most insignificant (see also Supplemental information Figure S19C for CO-electrooxidation CVs). On the other hand, as shown in Figure 6C (see also Supplemental information Figures S16 for more STEM images), larger nanoparticles have been observed in the case of Pt-Fe/C electrocatalyst, which correlates well with the larger degree of ECSA_CO_ loss (Figure 4B; see also Supplemental information Figure S19B for CO-electrooxidation CVs). One speculation and thus reasoning one might provide could be connected to the differences in M-C phase diagrams that can, depending on the nature of metal or alloy type, result in a formation of sometimes graphitized carbon shells on the surface of the particles (Baldizzone et al., 2014; Chong et al., 2018; Mezzavilla et al., 2016; Wang et al., 2019a; Xiao et al., 2019). Solubility of carbon in Ni is the highest among the inspected Pt-M systems, and recent studies suggest a possible inhibitory effect of carbon shell on Pt dissolution (Yamada et al., 2020). Lastly, Pt-Co/C electrocatalyst analyzed after ADT in Figure 6H (see also Supplemental information Figure S17 for more STEM images) shows again a mix of small and large nanoparticles with both a core-shell or a porous crystal structure. In addition, however, we have observed a nanoparticle with a carbon nanotube growing out of it (Figure 6H). However, because we do not know the history of this specific nanoparticle, what we cannot conclude is if this feature originated as a result of ADT, PCA, or perhaps even goes all the way back to the thermal annealing process of Pt-Co alloy formation.

Looking at all the exposed differences, we again wish to note that it would be impossible to keep these parameters constant for all the samples and just vary M. There are many fundamental thermodynamic and kinetic differences that arise simply due to varying M that highly affect the complex structure-property relationships. Thus, when studying structure-property relationships, the most acceptable way is when comparing electrocatalysts from the same batch, which are for example altered with additional mild annealing step (e.g. to induce crystal order or disorder (Hodnik et al., 2014)), the addition of some dopant or decoration (e.g. with Au (Gatalo et al., 2016; 2018; Jovanovič et al., 2016; Ruiz-Zepeda et al., 2017) or Ru (Jovanovič et al., 2018a)), or oxidation of the metal in its oxide form (e.g. Ru (Hodnik et al., 2015) or Ir (Jovanovič et al., 2017b)). This way, at least many initial parameters such as the particle sizes, loading, chemical composition, and others stay as similar as possible. Nevertheless, such an approach still makes it rather difficult to study true structure-property relationships. However, it is good for testing trends of behavior in a trial and error (“semi serendipity”) approaches.

#### Online metal dissolution of Pt-M/C electrocatalysts (EFC-ICP-MS)

Although classical analysis provides many clues on the differences in the behavior of Pt-M/C electrocatalysts, advanced methods are necessary in order to get a deeper insight. For that reason, we for the first time provide a comparison between all four widely reported Pt-M/C electrocatalyst systems (M = Cu, Fe, Ni, and Co) using our advanced electrochemical flow cell coupled to an inductively coupled plasma mass spectrometer (EFC-ICP-MS) methodology that enables an online ppb-range time- and potential-resolved dissolution dynamics of metals (Ahluwalia et al., 2018b; Jovanovič et al., 2014, 2017a, 2017b; Pavlišič et al., 2014, 2018). Both Pt and M dissolutions are followed as a function of potential cycling, with the aim to gain insights into the dissolution behavior out of different Pt-M alloys, obtain periodic trends, and also expose any potential differences between them.

**Figure 7:**
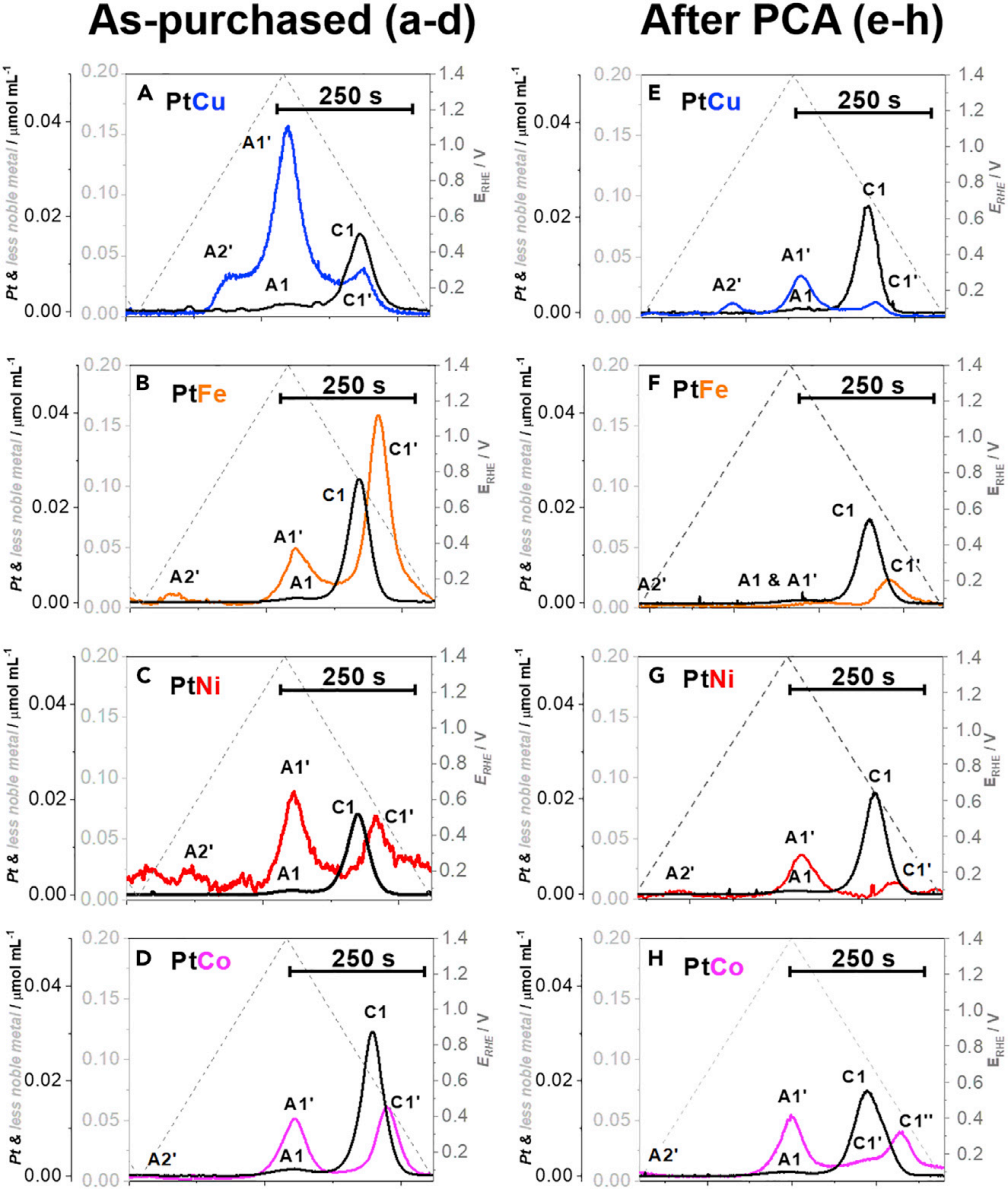
EFC-ICP-MS comparison of metal dissolution from Pt-M/C FCS electrocatalysts in the as-purchased state and after PCA Close-ups of a single cycle from 0.05 to 1.4 V_RHE_ for all Pt-M/C electrocatalysts in the (A–D) as-purchased state and (E–H) after PCA (200 cycles in 0.1 M HClO_4_, 0.05–1.2 V_RHE_, 300 mV s^−1^). See also Supplemental informationFigures S21–S24 for further comparisons of EFC-ICP-MS experiments. The colors (Pt-Cu = blue, Pt-Fe = orange, Pt-Ni = red and Pt-Co = magenta) correspond to the ones used for the graphs and borders in all of the figures.

In the first set of measurements, the dissolution behavior was monitored for all four Pt-M analogues in the as-purchased state (Figures 7A–7D; see also Supplemental information Figures S21–S24 for more EFC-ICP-MS data) by performing slow cycles (5 mV s^−1^) from 0.05 to 1.X V_RHE_ (X = 0, 2 or 4; raising the upper potential limit – UPL). The goal was to first gain insight into the intrinsic dissolution mechanisms of the different Pt-M alloys in their pristine ‘mixed” state and later compare that with the de-alloyed Pt-M alloys after PCA when the surface of the nanoparticles becomes more Pt-rich (activated). Pt dissolution profiles are in accordance with the previous literature reports, where smaller anodic (A1) and higher cathodic (C1) Pt dissolution peaks are observed (Cherevko et al., 2016; Gatalo et al., 2019b; Jovanovič et al., 2014; Topalov et al., 2012, 2014). Anodic (A1) Pt dissolution mechanism involves surface structure roughening caused by oxide place exchange mechanism (Topalov et al., 2014). This creates Pt defects (low coordination sites), which do not get passivated by oxide formation and are thus prone to dissolution (Cherevko et al., 2016), whereas cathodic (C1) dissolution occurs when Pt-oxide reduces, which again restructures and disturbs the Pt surface and induces the dissolution of Pt defects. Furthermore, what is also common across all Pt-M/C (not only those investigated in this study) is that the dissolution of M always follows the dissolution of Pt. In other words, every time Pt dissolves, this exposes previously protected M atoms and causes their subsequent dissolution (Gatalo et al., 2019b). This is observed in both the anodic scan direction (during Pt oxide formation; peaks A1 for Pt and A1′ for M) as well as cathodic one (during Pt oxide reduction; peaks C1 for Pt and C1′ for M). Thus one can conclude that in order to stabilize Pt-alloys, the focus should be more on stabilizing Pt itself than on the less-noble metal (e.g. addition of Au (Chung et al., 2020; Gatalo et al., 2016; Kodama et al., 2016; Lopes et al., 2016, 2020)). We note that when Pt does not protect M the dissolution of M can also be observed (for example peak A2′). This is possible either *via* the process of direct dissolution of un-alloyed M (such as in the case of Pt-Co, Pt-Ni and Pt-Fe in this study) or due to the stronger M-Pt surface interaction (such as the UPD interaction between Pt and Cu (Gatalo et al., 2019b)). This is typical for the catalysts that have not been properly activated by, for instance, washed with acid.

The main difference between all four Pt-M analogues appears when comparing the dissolution mechanisms. We look at which M dissolution peak becomes the dominant one (in other words, whether more M is dissolved during Pt oxidation or oxide reduction, the ratio of the A1’/C1′ intensities) upon increasing the upper potential limit (UPL) to potentials as high as 1.4 V_RHE_ (Figures 7A–7D; see also Supplemental information Figures S21A–23A). Based on this criterion, we can divide the investigated Pt-M electrocatalysts into two groups. In the first group, Pt-Cu and Pt-Ni analogues (Figures 7A and 7C; see also Supplemental information Figures S21A and S23A) upon increasing the UPL to 1.4 V_RHE_ exhibit more dominant anodic dissolution in comparison to cathodic (group one: A1’ > C1′), which is especially evident for Pt-Cu. This rather modest cathodic dissolution of Cu can be attributed to the additional UPD interaction of Cu with Pt surface that results in partial re-adsorption of dissolved Cu to the Pt surface during the cathodic scan (Gatalo et al., 2019b), whereas this is not the case for Pt-Ni. In the case of Pt-Cu, this then results in the higher intensity of peak A2′ in the following cycle (Gatalo et al., 2019b), In the second group, M dissolution trend is reversed. Upon increasing the UPL, Pt-Fe and Pt-Co analogues exhibit more dominant cathodic dissolution of M (Group two: A1’ < C1′) with the most evident case being Pt-Fe (Figures 7B and 7D; see also Supplemental information Figures S22A and S24A). One could assume that Pt-Fe has the highest portion of M in the near-surface region (a result of nanoalloy anisotropic inhomogeneity) or has the fastest surface segregation of M (followed by Pt-Co > Pt-Ni ≥ Pt-Cu) that results in the larger portion of Fe dissolution occurring only during the cathodic dissolution (Cui et al., 2013; Gan et al., 2014; Ruiz-Zepeda et al., 2019).

In order to examine if the dissolution mechanism changes upon electrocatalyst activation (PCA), the second set of measurements was performed. The as-purchased Pt-M/C electrocatalysts were first pre-treated by the same PCA protocol used in the case of TF-RDE characterization (Figure 4) prior to performing slow cycles (5 mV s^−1^) from 0.05 to 1.X V_RHE_ (X = 0, 2, or 4; again increasing the UPL). This is relevant for practical PEMFC applications, as several recent reports already provide evidence that M cations (e.g. Ni, Co, Cu, Fe, …) contamination in the PEMFC are decreasing the high-current density performance in MEA (Ahluwalia et al., 2018a; Braaten et al., 2017, 2019; Papadias et al., 2018). Thus, it is of particularly high importance to understanding how intrinsic dissolution trends change after PCA for different Pt-M alloy systems when the surface of the nanoparticles becomes Pt rich, namely, core-shell. Interestingly, even after all four Pt-M analogues were subjected to PCA, the division by comparison of the A1’/C1′ intensities remains the same (Figures 7E–7H; see also Supplemental information Figures S21–S24B). In the case of Pt-Cu and Pt-Ni analogues (Figures 7E and 7G; see also Supplemental information Figures S21B and S23B), upon raising the UPL to 1.4 V_RHE_, anodic dissolution of M is increasing in dominance (Group one: A1’> C1′). On the other hand, in the second group (Pt-Fe and Pt-Co analogues), M dissolution trend is once again reversed with again the most evident case being Pt-Fe (Figures 7F and 7H; see also Supplemental information Figures S22B and S24B). In other words, for each respective Pt alloy, the intrinsic mechanism of dissolution does not change after a substantial amount of M has been removed from the near-surface region of the nanoparticles. Furthermore, the amount of Pt dissolution remained on the same order of magnitude, whereas we noticed a drastic decrease in the amount of M dissolution. Activation (de-alloying) of Pt-M/C electrocatalysts depletes the surface and near-surface layers of M, which creates a Pt-rich overlayer that limits the dissolution of M. However, as noted before, dissolving of Pt nevertheless still results in the further dissolution of M, thus showing that stability of Pt is the limiting factor for using Pt-alloy electrocatalysts in real PEMFCs.

We believe it is important to stress some general pros and cons of each of four less noble metals for the PEMFC application. Each nanoalloy, in terms of their application in the PEM-FC, comes with not only their benefits but also restrictions. It is of paramount importance to be familiar with strong and weak points of each metal, so we know what to optimize in the development of each of them. Here we present our opinion in terms of a few pros and cons that could serve as guiding rules for planning PEMFC Pt-alloy electrocatalyst synthesis and application (Box 1).

Box 1 **Pros and Cons Pt-M/C (M = Cu, Ni, Fe, Co) electrocatalysts for the PEMFC application.**

Based on the comparisons from the present study, looking merely on the long-term ORR activity and the resilience toward the leaching of M, **Pt-Cu** seems as the best choice. Another benefit of this alloying combination is that it easily forms the intermetallic phase (Bele et al., 2014; Gatalo et al, 2019a, 2019b, 2019c, 2019d; Hodnik et al., 2012a, 2014; Pavlišič et al., 2016), which was shown to slow down the leaching of M (Pavlišič et al., 2016). Furthermore, due to very low carbon solubility in Cu (Li et al., 2009), encapsulation of Pt-M nanoparticles with a carbon shell due to high-temperature treatments necessary for (intermetallic) Pt-alloy formation is not a concern. On the downside, many negative effects of Cu ions on the PEMFC performance have been shown (Yu et al., 2012; Zhu et al., 2020). Thus, for even the slightest possibility of sensible and successful implementation of Pt-Cu alloy electrocatalysts in the industry, significant improvements in stability of Pt-alloys during real-time operation. In other words, one would need to eliminate Cu leaching entirely. In that sense, ternary alloys that focus on the stability of the less noble metal could lead to significant improvements (Gatalo et al., 2016; Tu et al., 2020).

**Pt-Fe** alloy seems like a good choice for similar reasons as Pt-Cu. Formation of the intermetallic phases is rather facile and widely present in the literature (Liu et al., 2019; Wang et al., 2019b; Wittig et al., 2017). However, in terms of applicability of Pt-Fe alloy electrocatalysts in PEMFC, similarly, but perhaps even more significantly, as in the case of Cu, even slightest amounts of Fe ions in the PEMFC are not acceptable. This is because Fe ions catalyze the formation of radicals via Fenton reaction (Strlič et al., 2003), which increases the rate of degradation of the proton-exchange membrane, resulting in failure of the PEMFC. Moreover, Fe is used as a model impurity to study such processes and stability of proton-exchange membranes in PEMFC (Singh et al., 2018). As it was shown in the current study, all Pt-M alloy electrocatalysts leach at least very small amounts of the less-noble metals even after PCA protocol and de-alloying of larger quantities of M from the surface and near-surface regions. Therefore, our opinion of Pt-Fe alloy is similar to that of Pt-Cu, and its applicability is the industry is widely reliant on the ability of significantly improving the stability of Pt and real-time operation of PEMFCs.

**Pt-Ni**, on the other hand, does not form intermetallic phases as easily as for instance Pt-Cu or Pt-Fe. We presume that this could be connected to the orders of magnitude higher dissolution of carbon in Ni compared with for example Cu (Li et al., 2009) that prevents facile crystallization due to the ternary nature of the Pt-Ni-C phase system where carbon acts as an impurity in the crystal lattice. An additional issue related to the solubility of carbon in Ni is consequential encapsulation of Pt-Ni nanoparticles with a rather thick carbon shell (Stamenković et al., 2007b) as a result of high-temperature treatments necessary for the (intermetallic) Pt-alloy formation. This imposes limitations to the high-temperature treatments in order to avoid the carbon encapsulation issues while achieving the intermetallic crystal structure. Furthermore, carbon shells, while showing possible benefits in durability, have been shown to negatively influence the high-current density performance in the PEMFC, possibly affecting the O_2_ transport resistance (Yamada et al., 2020). In addition, the presence of a carbon shell requires additional efforts for adequate chemical activation of such electrocatalysts and exposure of active Pt-surface area such as with the use of ozone (Baldizzone et al., 2015b). On the bright side, however, Ni dissolution has a much less detrimental effect on proton-exchange membrane degradation as Fe or even Cu ions (Braaten et al., 2019; Strlič et al., 2003). Furthermore, due to its low redox potential it does not block the Pt surface as Cu. Therefore, Pt-Ni could be a viable candidate and has already well been demonstrated in the PEMFCs (Ahluwalia et al., 2018a; Dionigi et al., 2019; Han et al., 2015; Myers et al., 2015). Nevertheless, Ni ions have been shown to also have a negative effect on the O_2_ transport resistance as well as influence the water uptake in the ionomer (but less detrimentally as Cu), thus affecting the three-phase-boundary and thus high current density performance in PEMFC (Braaten et al., 2019). Thus, in addition to the above-stated Pt-Ni alloy synthesis challenges, significant improvements in the resilience of Pt-Ni alloy electrocatalysts against Ni leaching are still necessary for its successful deployment in end-user products.

Lastly, Pt-Co alloy has already shown presence in the end-user products (Lohse-Busch et al., 2018; Yoshida and Kojima, 2015), and there also seems to be some rationale behind why. Firstly, any issues related to carbon solubility and encapsulation seem to be much less detrimental as in the case of Ni, showing much more similar behavior to that of Pt-Cu. Furthermore, the formation of intermetallic structures is possible (Kongkananad, 2020; Cui et al., 2020; Xiong et al., 2019), whereas the behavior of Co^2+^ ion as an impurity in the PEMFC currently also points toward similar impacts as that of Ni^2+^(Braaten et al., 2017, 2019). Similarly again to Ni, it also does not block the Pt surface. The biggest possible issue of Co, however, is in its controversial mining practices, as already highlighted in the Li-ion battery-electric vehicle segment where large efforts are going toward initially minimizing and finally completely eliminating the use of Co (Author Anonymous, 2020b). However, we note that the quantities of Co in PEMFC are much lower than in batteries (a few grams per vehicle in contrast to many kilograms in battery-electric vehicles). Thus, the usage of Co in PEMFC vehicles might not be such a huge issue after all. Nevertheless, its origins must be tracked either from recycled batteries or via fair trade mining. Analogously to the Pt-Ni alloy system, however, significant improvements in the resilience of Pt-Co alloy electrocatalysts against Co leaching are still necessary to reach deployments in end-user products while continuously reducing the required amounts of Pt.

#### Corrosion of the carbon support (EC-MS)

Studying the degradation behavior of the Pt-alloy nanoparticles is only part of the necessary efforts to understand and improve supported electrocatalysts. The other necessary component is a high-surface-area conductive material that enables high dispersion of Pt and thus its high utilization at industrially relevant loadings of metal nanoparticles (>30 wt%) (Kongkanand and Mathias, 2016; Padgett et al., 2019; Yarlagadda et al., 2018).

Firstly, the morphology of carbon substrates plays a major role in determining the utilization of Pt. Pt-based nanoparticles located in micropores of carbon blacks are known to have sub-optimal mass transport, resulting in lower Pt utilization. In addition, past studies have revealed that many properties of the carbon supports, such as degree of graphitization, surface area, porosity type and size, functional groups etc., play a significant role not only on Pt utilization but also on carbon corrosion (Antolini, 2009; Kongkananad, 2020; Castanheira et al., 2014; Padgett et al., 2019; Tang et al., 2014; Trogadas et al., 2014; Yarlagadda et al., 2018). Other studies also point toward the importance of functional groups (such as N-functionalization (Ott et al., 2020)) present in the support material, because they can greatly impact the distribution of the ionomer and thus, positively impact the performance of the Pt-based electrocatalysts. Finding the optimal interplay between the stability of carbon supports and other properties, such as type and size of porosity, is one of the crucial parameters that need to be considered when designing a stable electrocatalyst that will also enable adequate performances at high current densities in PEMFC (Kongkananad, 2020; Padgett et al., 2019; Yarlagadda et al., 2018).

Currently, the most widely used supporting materials are commercially available carbon blacks (such as Vulcan XC72 and Ketjen Black EC300J in this study), which offer a high surface area, low cost, high electrical conductivity, etc. (Kongkanand and Mathias, 2016; Padgett et al., 2019; Stephens et al., 2012). Despite these carbon supports being relatively stable under relevant electrochemical conditions, carbon corrosion is still one of the inherent largest contributors to the loss of ECSA as a result of secondary degradation mechanisms of Pt-based nanoparticles, resulting in the agglomeration and/or detachment (Gu et al., 2007; Maselj et al., 2020; Meier et al., 2014). Slowing down support corrosion is a major challenge; on one hand, electrochemical carbon oxidation is thermodynamically feasible already at a very low standard electrode potential E_CO2/C_^0^ = 0.207 V_RHE_ and on the other, the kinetics of oxidation is accelerated by Pt, especially at the operational conditions of the PEMFC (e.g. 80°C) (Maselj et al., 2020; Pizzutilo et al., 2016).

The here-in investigated materials are supported either on Vulcan XC-72 (Hi-Spec 4000, all Pt-M/C FCS materials) or on Ketjen Black EC300J (TEC10E50E, TEC10E50E-HT, and Elyst Pt50 0550) carbon black supports. The corrosion behavior of these materials can be assessed by tracking the CO_2_ released from the material as a consequence of the applied electrochemical protocol. This can be done by direct coupling of a thin-layer electrochemical cell to a mass spectrometer through a porous interface that enables transition of volatile compounds formed at the working electrode. The so-called electrochemistry–mass spectrometry (EC-MS) technique is described in detail elsewhere (Trimarco et al., 2015, 2018) and is equivalent in its general principle (detecting electrochemically formed volatile species) to the differential electrochemical mass spectrometry (DEMS) or on-line electrochemical mass spectrometry (OLEMS) (Ashton and Arenz, 2011; Grote et al., 2014). Given that Pt-M/C electrocatalysts are all supported on the same type of carbon black (Vulcan XC-72) and have very similar macro properties of the dispersed metal, it is not surprising to see that their carbon degradation behavior, shown in Figure S25A, is indistinguishable. This also suggests that the M ions leached from the alloys—while crucial for the degradation of fuel cell membranes—do not significantly impact the carbon degradation under the tested conditions (i.e. room temperature, low number of potentiodynamic cycles). In contrast, the degradation of carbon in the Pt/C electrocatalysts (Figure S25B) gives notable higher CO_2_ signals than the Pt-M/C materials. This is most likely a consequence of higher Pt loading, which is around 14–18 wt% for the alloy Pt-M/C electrocatalysts and in the range of 40–50 wt% for the Pt/C materials.

Alternatively to the materials in the present study, graphene-based materials could address the limitations of the commercially available carbon black supports by offering unique properties, leading to the required improvements of addressing both the porosity and durability issues (Antolini, 2009; Higgins et al., 2016; Hu et al., 2017). Last but not least, conductive ceramic-based supports may one day replace the carbon-based materials (Uchida, 2020). In our opinion, for that to happen, the ceramic alternatives would have to match or exceed current state-of-the-art carbon-based supports in every single crucial property, while also showing improved durability. In our opinion, however, one of the biggest barriers will be matching the excellent conductivity of carbon-based supports.

Although time- and potential-resolved EFC-ICP-MS methodology, EC-MS, and standard techniques for determining the materials structural properties and electrocatalytic behavior provide an enormous amount of relevant and useful data, such methods alone will not be enough for the much-needed breakthroughs that are necessary for the deployment of stable Pt-alloy electrocatalysts and the crucially significant decrease of the required Pt for PEMFC operation. We believe we provided enough data to establish the complexity of the studied systems as well as expose the risks of only using a top-down approach for the interpretation of the structure-property functions of nanoparticulate bulk systems. Moving forward, we wish to present you a bottom-up methodology that enables direct study of the structure-property relationships of one nanoparticle at a time.

### Bottom-up approach to directly study structure-property relationships

Because the complexity of nanoparticles at the atomic level is enormous, it is necessary to study the evolution of exactly the same nanoparticles by playing the game of “Spot the difference,” i.e. by finding the changes in the arrangement of atoms in the particle (Hodnik and Cherevko, 2019; Ruiz-Zepeda et al., 2019). In other words, in order to be able to reliably interpret the structural changes and improve our understanding of the structure-property relationships, it is necessary to know the nanoparticles' history by comparing how the structure looked not just after an electrochemical treatment but also before. Everything else is comparing “apples and oranges.” However, due to a much wider variety and complexity of metallic nanoparticles (Aarons et al., 2017; Li et al., 2014; Zalitis et al., 2020), it is not possible to follow a similar approach as in other disciplines where image analysis is employed to collect structural information from multiple individual motives in the sample. As for instance the use of cryogenic electron microscopy (cryo-EM) analysis in the field of biology, where viruses, proteins, and molecules, in general, have defined structures, and it is presumed that all of the motives investigated have the same structure, just different rotation; and it is then appropriate to use these images as projections to build a 3D representation (Luque and Castón, 2020). Due to a much wider variety and complexity, we cannot do the same with metallic nanoparticles. At the same time, it is important to study the effects of conditions that closely mimic those practically experienced by the material in MEA. Only then can conclusions be relevant for FC applications. Although in-situ liquid microscopy would, of course, provide the most straightforward insights into potential-induced structural changes, the beam-induced side reactions (e.g. radiolysis) still present a problem that has not been overcome (Hodnik et al., 2016).

#### IL-(S)TEM in combination with electrochemical characterization using modified floating electrode (MFE) methodology

IL-TEM is a method that allows tracking of changes in the individual nanostructures. Since the inceptions of the methodology by K. J.J. Mayrhofer et al.(Meier et al, 2012a, 2012b, 2014; Schlögl et al., 2011), the game of “Spot the difference” (Scheme 2) (Hodnik and Cherevko, 2019) has been recently re-defined to retrieve information at the atomic scale (Jovanovič et al., 2018b; Rasouli et al., 2019; Ruiz-Zepeda et al., 2019), adapting it to something more close to a semi *in-situ* method. Crucially, atomic-resolution electron microscopy images provide much more information than just those recorded with a nanometer-scale resolution. Due to advancements in high-resolution TEM, we are, in recent years, for the first time gaining the ability to start answering some of the fundamental questions related to the structure-property (e.g. stability) relationship of nanostructures. That is, we have gained the ability to answer the questions if and how does even an individual nanoparticle change on the atomic scale upon a specific electrochemical treatment by being able to compare its exact structure before and after (Hodnik and Cherevko, 2019). As we have shown in this work so far, a top-down approach enables studying of average properties (namely TF-RDE, XRD, EFC-ICP-MS, EC-MS, ICP-OES) as well as some of the more local, however still considered average, properties (*ex-situ* (EDX) TEM). Because the complexity of real electrocatalysts is immensely high, especially at the atomic level, many of our conclusions have to come down to the presumptions and interpretations based on the investigation of similar electrocatalyst systems published before. Similarly can be said for the *ex-situ* TEM imaging where on the contrary to IL-TEM, in the random *ex-situ* imaging of electrocatalysts before and after the history of the observed nanostructures is not known and thus for particular features is hard to draw exact conclusions. Thus, as part of this work, we wish to showcase our bottom-up IL-TEM approach that enables studying structure-stability relationships.

**Scheme 3:**
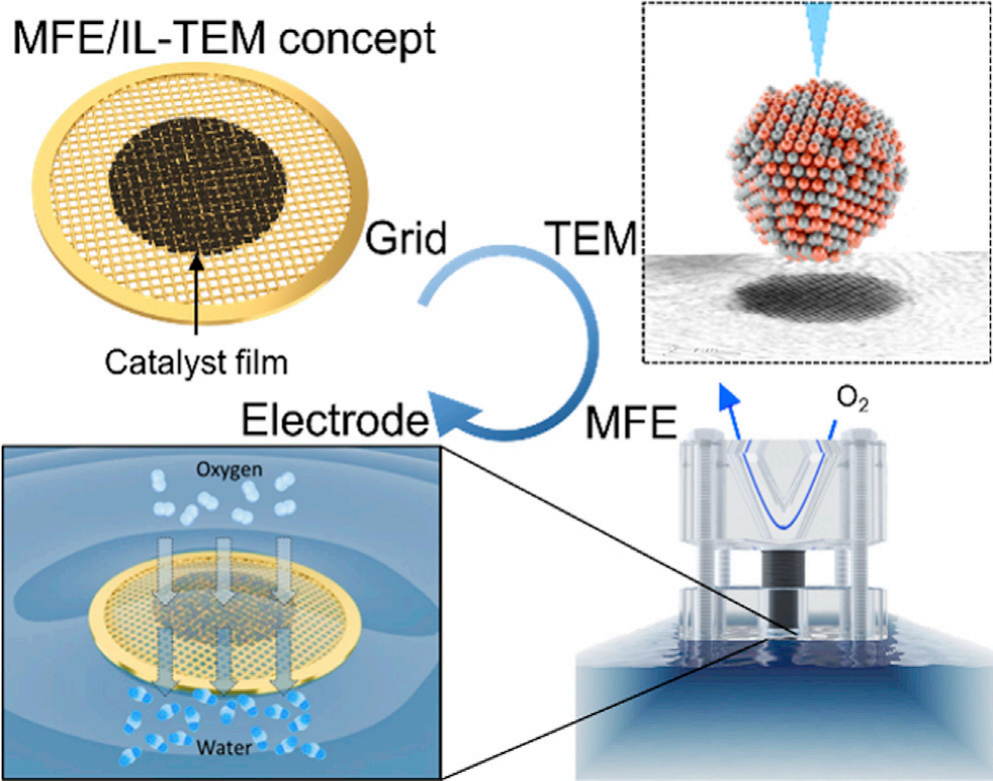
Graphical representation of the MFE and IL-TEM methodology concept The catalyst film deposited on a TEM grid can be investigated at atomic resolution under the microscope before being exposed to electrochemical treatment in a MFE cell where enhanced oxygen-mass-transport conditions provide realistic MEA current densities. Afterward, the TEM imaging can be repeated on the same location on the material to analyze the electrochemically induced changes to the structure of nanoparticles at an atomic level.

In order to advance fundamental studies from the conventional TF-RDE methodology to the actual application, namely industry-relevant (high) current densities, it would be beneficial to expose Pt-based nanoparticles to the enhanced mass transport conditions. For this, we introduce the modified floating electrode (MFE) platform (Scheme 3) (Hrnjić et al., 2020). MFE allows using a TEM grid as a working electrode and thus is synergistic with the IL-TEM methodology (Hodnik and Cherevko, 2019; Hrnjić et al., 2020). The main characteristic of the MFE setup is that the working electrode operates in the so-called floating mode (Lin et al., 2020; Zalitis et al, 2013, 2015, 2020). This means that the electrode is not dipped in the electrolyte solution, as is the case with TF-RDE, but is instead placed on its surface. Because the electrode is floating on the liquid phase, gas reactants (in the present case oxygen) are delivered almost directly from the gas phase (or at least through a very thin liquid layer). Consequently, fast mass transport to the electrocatalyst layer is enabled, which allows inspection of substantially higher ORR current densities compared with the TF-RDE methodology.

For demonstrative purposes, the MFE measurements and corresponding IL-(S)TEM analysis were performed on Pt-Co/C electrocatalyst solely.

**Figure 8:**
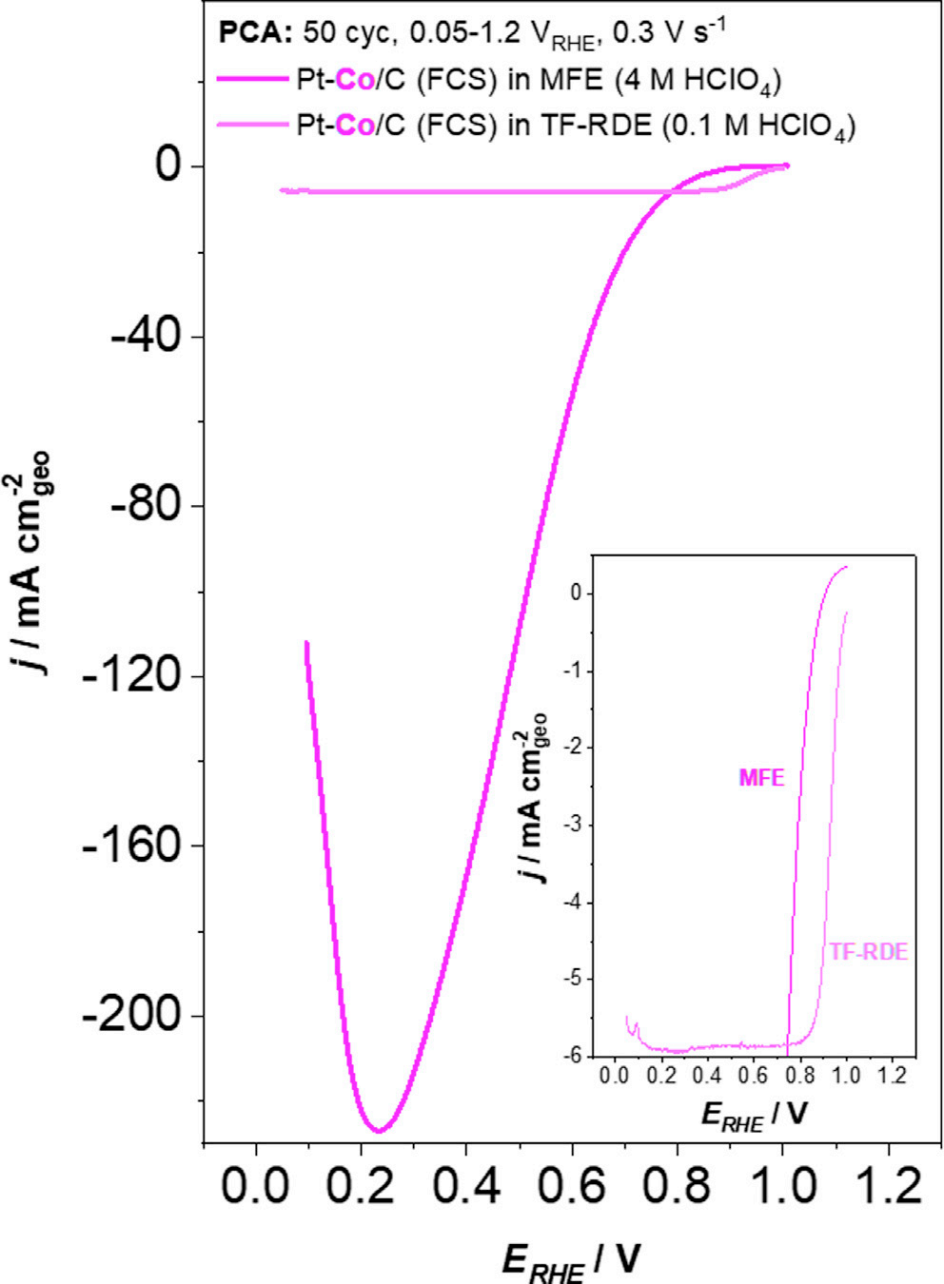
Comparison of Pt-Co/C FCS electrocatalyst ORR polarisation curves measured by MFE and TF-RDE Comparison of ORR polarization curves for the Pt-Co/C FCS electrocatalyst obtained with conventional TF-RDE (0.1 M HClO_4_, performed at 1600 rpm) and MFE (4 M HClO_4_) methodologies after PCA. Only the current response in the anodic scan is shown. The color (Pt-Co = magenta) correspond to the one used for the graphs and borders in all of the figures.

By introducing oxygen to the top of the working electrode, a well-resolved corresponding ORR polarization curve is obtained (Figure 8). The most obvious feature is that the MFE current densities significantly exceed the ones obtained in TF-RDE setups. Evidently, in contrast to TF-RDE (Mayrhofer et al., 2008) no mass-transport-limited polarization regime is expressed in the MFE setup where a maximal current density of 225 mA cm^−2^_geo_ is obtained. For comparison, under most elevated hydrodynamic conditions still reachable to state-of-the-art RDEs, i.e., 10,000 RPM, the theoretical mass transport limited current density is only approximately 14 mA cm^−2^_geo_ for ORR according to the Koutecký–Levich equation (1600 rpm and 5–6 mA cm^−2^_geo_ in the present study) (Hrnjić et al., 2020; Kucernak and Toyoda, 2008). However, we stress that at this point only a rough comparison between the two methods is possible. Namely, as seen from ORR comparison of the two setups higher current densities in comparison to MFE are obtained in TF-RDE case above ~0.8 V. Firstly, this discrepancy can be due to the different concentration of acidic electrolyte (4 M for MFE in contrast to 0.1 M for TF-RDE, respectively) which maximizes blocking of Pt ORR active surface by adsorption of ClO_4_^−^ anions (Láng and Horányi, 2003; Omura et al., 2014; Sievers et al., 2019; Zalitis et al., 2020). Secondly, substantially different electrocatalyst loadings were used depending on the method (0.9 μgPt cm^−2^_geo_ for MFE in contrast to 18 μgPt cm^−2^_geo_ for TF-RDE). In addition, it should also be noted that ORR response under conditions of elevated mass transport such as the ones present in the MFE or similar setups is typically strongly related to several experimental parameters. These encompass electrocatalyst loading, ink composition (choice of solvents, I/C ratio, additives), presence of metal impurities etc. All of these need to be separately investigated, as they affect both the ORR kinetic and mass transport at high current density. Therefore, much work still needs to be done in order to understand how the differences in experimental setup affect the electrochemical performance.

**Figure 9:**
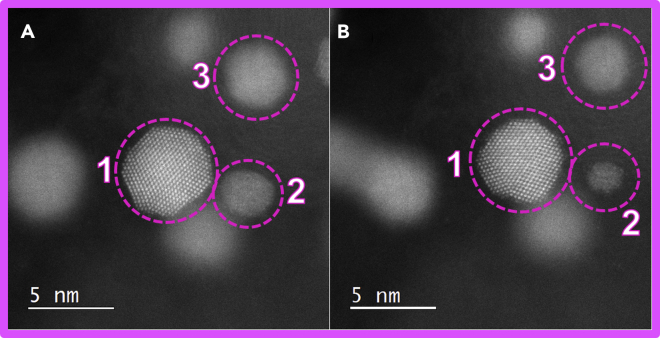
IL-STEM comparison of Pt-Co/C FCS electrocatalyst before and after PCA IL-(S)TEM ADF comparison of Pt-Co/C FCS electrocatalyst before (A) and after (B) PCA using MFE methodology. The color (Pt-Co = magenta) correspond to the one used for the graphs and borders in all of the figures.

To showcase our novel bottom-up approach platform combining IL-(S)TEM and MFE methodologies, we are comparing the Pt-Co/C FCS electrocatalyst before and after an electrochemical activation protocol (PCA; 200 cycles in 4 M HClO_4_, 0.05–1.2 V_RHE_, 300 mV s^−1^). A “low-magnification” comparison of Pt-Co/C FCS electrocatalyst before (see Supplemental information, Figures S26A and S26B) and after PCA (see Supplemental information, Figures S26C and S26D) does not easily reveal any significant changes, with the exception of particle coarsening of some of the largest nanoparticles (above 10 nm). In order to reveal the structural differences, one has to compare the images with atomic-resolution before (Figure 9A) and after (Figure 9B) PCA. By closely examining the nanoparticles in focus, interesting differences can be observed. Focusing on nanoparticle number 1, we can clearly see a big difference in both the size of the particle (marked by a circle for easier evaluation of shrinkage) and the change in the morphology, namely exposed facets, resulting from PCA. In addition, we also clearly observe a decrease in the size of nanoparticles number 2 and 3; presumably, as a result of de-alloying of Co during PCA, both nanoparticles have significantly shrunk. What one can consider from both low and high magnification examples is that phenomena the electrocatalyst experiences during activation are similar as in the degradation phases. Although activation results in desirable phenomena such as initial de-alloying of Co and formation of Pt-rich overlayer, it can also result in less desirable phenomena such as agglomeration of nanoparticles and thus, loss of ECSA. The PEMFC scientific community will benefit not only from better understanding of the degradation of Pt-alloy electrocatalysts (Hodnik and Cherevko, 2019; Meier et al, 2012a, 2012b, 2014; Ruiz-Zepeda et al., 2019) but also from understanding the structure-property evolution during Pt-alloy electrocatalyst activation (Gatalo et al., 2019a, 2019b).

**Figure 10:**
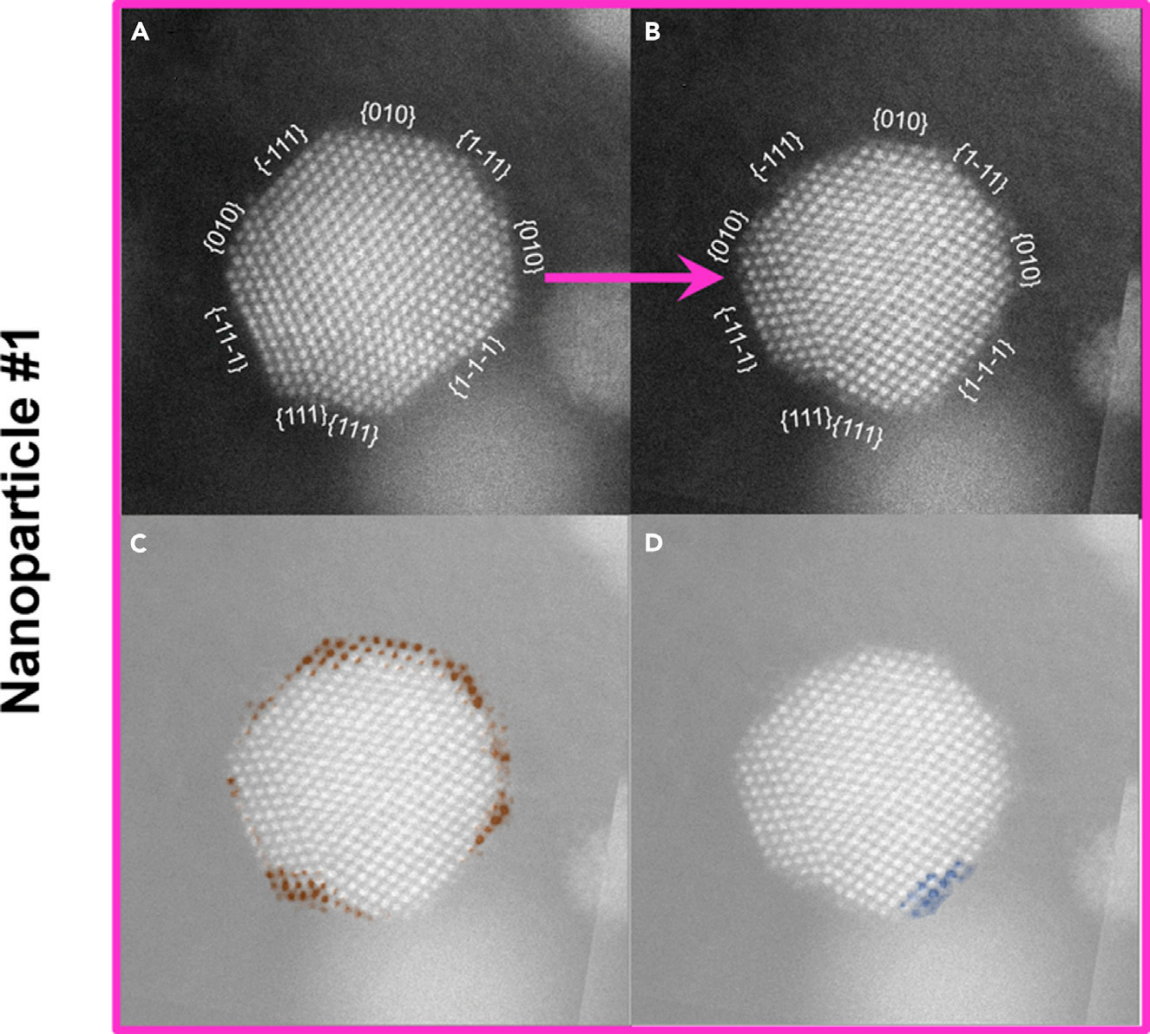
Atomically resolved STEM comparison of the same Pt-Co nanoparticle before and after PCA Atomically resolved dark-field STEM micrograph comparison of the same Pt-Co nanoparticle (number 1 in Figure 9) (A) before and (B) after an electrochemical activation (PCA; 200 cycles in 4 M HClO_4_, 0.05–1.2 V_RHE_, 300 mV s^−1^). Overlap of both images shows (C) disappearance of removed atomic columns colored in red and (D) appearance of new atomic columns colored in blue. See also Supplemental information for Figure S27 for similar representation of the evolution of atomistic columns as well as Figure S28 for another such example on a different Pt-Co nanoparticle. The color (Pt-Co = magenta) correspond to the one used for the graphs and borders in all of the figures.

To go one step further, we went in-depth with analysis of nanoparticle number 1 (Figure 10). More specifically, as a result of PCA (Figures 10A and 10B), the distribution of surface facets sizes is affected, with the main observation being the shrinkage of {111} facets. The overall effect gives the particle a less faceted and a more rounded shape. Interestingly, this goes in line with our previous studies of intermetallic Pt-Cu_3_ nanoparticles electrochemical shaping (Ruiz-Zepeda et al., 2019), where the disappearance of {111} facets has also been observed, despite the nanoparticles in that study having a different size, overall chemical composition, and different less-noble metal. At first glance, our past observations seem to intuitively contradict the findings from Pt single-crystal studies where Pt {111} was shown to be the most stable (and active) facet (Fuchs et al., 2020; Lopes et al., 2016, 2018). However, deeper inspection reveals that in-fact this observation is in line with the mentioned Pt single-crystal studies. In the abovementioned study of intermetallic Pt-Cu_3_ nanoparticles (Ruiz-Zepeda et al., 2019) that combined AR-IL-TEM experiments and Kinetic Monte Carlo simulations, we have concluded that the {111} facets were disappearing due to two reasons: (1) increased amounts of Cu in the corners of the nanoparticles and (2) higher dissolution of atoms from the less-stable neighboring Pt {100} and {110} facets, resulting in a phenomenon similar to “sharpening a pencil.” Thus, Pt {111} is indeed the most stable facet, but in nanoparticles, it will get attacked (etched) starting from the side weak-points such as less-stable facets, M-rich corners, defects, or edges. In the current case study of Pt-Co nanoparticle number 1, a close inspection reveals that the corners of the {111} facets are disappearing (Figure 10C, colored in red). We presume that the mechanism behind these observations is related to both the presence of steps and kinks (high-energy sites or less-stable sites) and, most likely, due to the higher local composition of Co that is getting de-alloyed during PCA. Interestingly, at the same time as the disappearance of some atomic columns, one can also observe the appearance of new atomic columns (Figure 10D, colored in blue). As already debated in the literature, this can be caused by either surface diffusion (redistribution of Pt) (Ruiz-Zepeda et al., 2019), redeposition of dissolved Pt during PCA from the electrolyte (Jovanovič et al., 2017a; Rasouli et al., 2019), or possibly even Ostwald ripening given that the nanoparticle number 2 has substantially shrunk (Figures 9C and 9D). We note that the most probable driving mechanism for this is the dissolution of low-coordinated sites from nanoparticle number 1 or the neighboring nanoparticles, followed by redeposition onto the available sites with high coordination number (i.e. {111} facets) (Rasouli et al., 2019). As we do not see the growth of {111} facets, in fact, they are disappearing, we believe the phenomenon of attachment of new Pt atoms is governed by surface diffusion on an individual nanoparticle (Pavlišič et al., 2016; Ruiz-Zepeda et al., 2019). Besides obvious disappearance of the Pt {111} facets, we also observed uniform dissolution of {010} facet at the top of the particle, as well as dissolution on corners and edges of {010} facets, which goes in line with our assumption that differently coordinated sites could be starting points for dissolution due to different binding energies (Campbell et al., 2002; Cherevko et al., 2016; Tang et al., 2010). These phenomena, however, need to be studied also on other nanoparticles first to confirm and secondly to truly understand the trends in more detail. We also note that (S)TEM images are only 2D projections of 3D objects, namely nanoparticles. In order to study the full 3D systems more projections are needed, namely one has to utilize 3D atomically resolved STEM tomography while having electron-dependent degradation and deformations in mind (Miao et al., 2016; Vanrompay et al., 2019; Yang et al., 2017).

**Figure 11:**
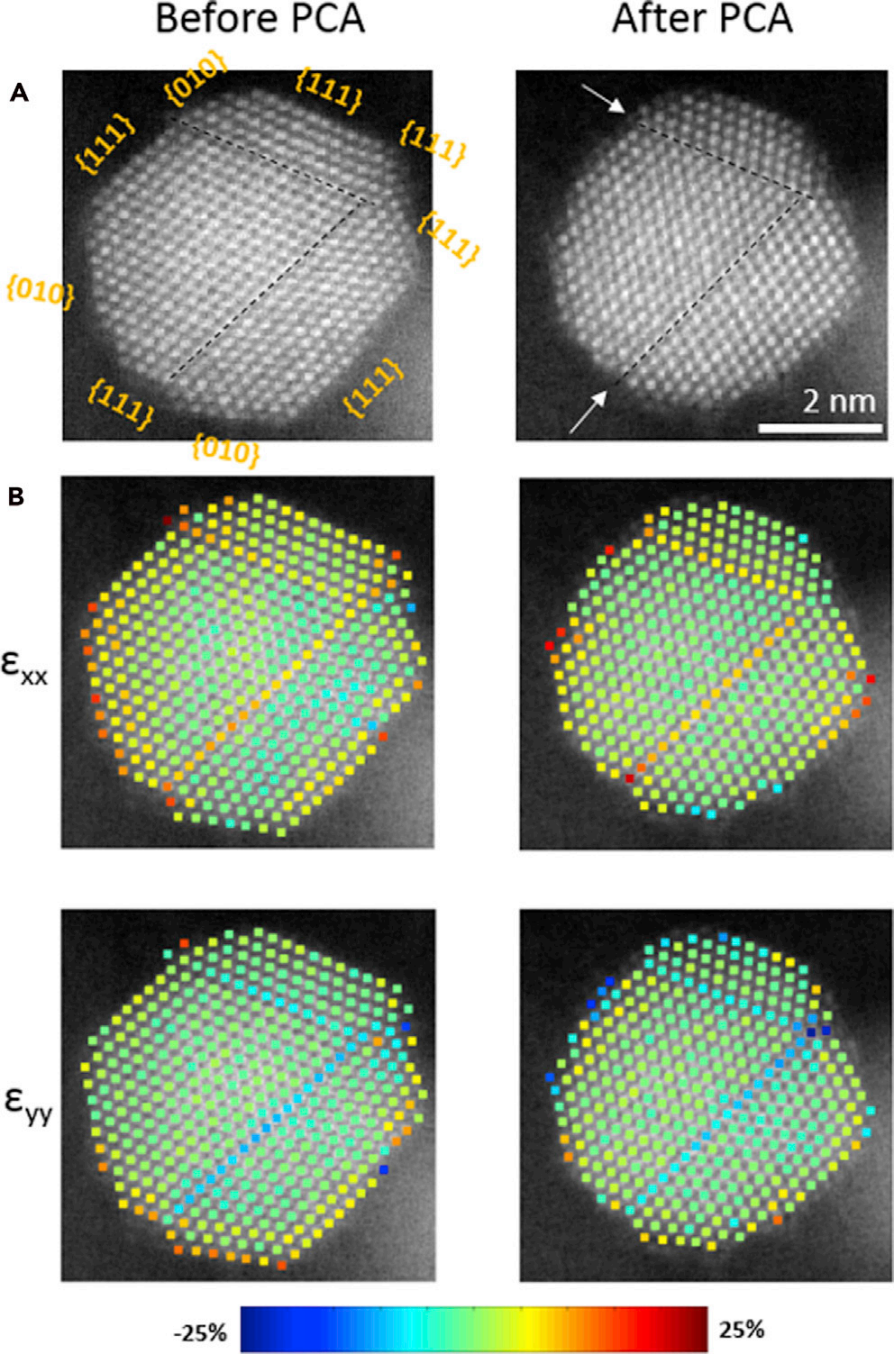
Strain map comparison on the same Pt-Co nanoparticle before and after PCA (A) A twinned PtCo nanoparticle (nanoparticle number 1 in Figure 9, but rotated) imaged before and after PCA with IL-(S)TEM ADF showing etching and reshaping effects. (B) Corresponding strain maps calculated for *ε*_*xx*_ and *ε*_*yy*_, respectively.

Although the “game” of “Spot the difference” (Scheme 2) on an atomic level is incredibly informative, the imaging also enables a more quantitative structural analysis. In addition to the identification of the disappearance and appearance of atomic columns, it is also of interest to analyze with great care changes in the observed nanostructure, as for instance strain in the nanoparticle. This is done by calculating derivatives from the measured displacements of the atomic columns when compared with an ideal reference (similar to the approach used in peak pairs analysis) (De Backer et al., 2017; Galindo et al., 2007; Ruiz-Zepeda et al., 2019; De wael et al., 2017). As can be noted from the strain maps in Figures 11B, the majority of the strain is distributed over the first two to three surface top layers of the nanoparticle, because for facets it has been shown that surface atom distances do not remain at the bulk parameter (Mamatkulov and Filhol, 2013). In addition, there is, as expected, an accumulated larger strain at the twins (marked with arrows in Figures 11A). The twins separate the crystal in three parts, hence the strain in each segment is computed piecewise in the maps using the internal lattice as reference. This helps to visualize the strain gradients within each domain (Johnson et al., 2008), facilitating the comparison of the deformations occurring in each segment individually, between the strain maps of before and after PCA. These kind of defects are characteristic of nanoparticles that may have had its origin during coalescence by a dislocation-mediated process (Luo and Guo, 2017). Overall, the strain maps of the nanoparticle reveal that facets have gained in average more compressive strain after PCA. That is, most of the facets have turned strain in a compressive or less tensile state. Interestingly, it is occurring in a similar trend for {111} and {100}, although some of them display variations along the same facet. This remark is important from the point of view of optimizing and designing the next catalyst. Identifying tensile or compressive strain for a particular facet is a crucial step to achieve a better performance of the catalyst, because the effect of surface strain on binding behavior has shown to be facet dependent (Luo and Guo, 2017). It is worth mentioning that in the present case, after PCA the particle has suffered dealloying, surface modifications, and size reduction (a change in volume), characteristics that strongly influence the strain distribution at the surface. Because the surface strain depends on the distribution of atoms located at the facets, vertices, and edges, it is reasonable to expect that twinned and non-twinned nanoparticles exhibit a different electrocatalytic performance. Moreover, it has been suggested that the defect structure of bimetallic nanoparticles differs from that of particles made of a single atomic species (José-Yacamán et al., 2007). Even though there are three important nanostructure factors that govern most of the electrocatalytic performance of the catalyst, the ensemble effects (presence and location of Pt and metallic-M atoms on the surface), ligand effects (corresponding electronic structure of surface Pt atoms due to neighboring M atoms), and strain effects (atomic spacing of surface Pt and/or M atoms), the last one is considered as the more significant (Luo and Guo, 2017). As has been shown earlier by other groups, surface strain parameter governs most of the ORR activity of Pt-based electrocatalysts (Mistry et al., 2016; Strasser et al., 2010), and lattice information from IL-(S)TEM provides such direct evidence on the exact location of these relative changes. Knowledge of strain distributions could lead to a better understanding of structure-properties relationships. It is therefore of high importance to conduct research focusing on the influence of strain on the stability of ORR electrocatalysts in relation to their performance.

We, however, do not know yet how this affects stability and understand that this adds to the complexity of the interpretation of Pt-alloy electrocatalysts activity changes. Thus, only further systematic studies, coupled with modeling, will start revealing insights and trends. For now, we only expose the fact that is not as straightforward as with the use of perfect core-shell model systems.

Finally, yet importantly, such an approach enables us, due to atomically resolved structure, to estimate the coordination of surface atoms. In addition to studying stability, this also opens a possibility of calculating the activity *via* the gCN theory (Calle-Vallejo et al., 2015) and also how this evolves with structural changes, namely stability. This can be tackled more specifically by theoretical calculations such as modeling or density functional theory (Ellaby et al., 2020; Fortunelli et al., 2015; Schnur and Groβ, 2010; Wu et al., 2012). However, we again note that full 3D structural information is needed, which without tomography is not possible in real nanoparticles due to severe complexity of their shapes, whereas for perfect polyhedral shapes one could estimate its shape from just one STEM appropriately orientated atomically resolved micrograph.

As exemplified on (for the most part) a single Pt-Co nanoparticle, the combination of experimental, computational image analysis and theoretical work is essential to correlate the information gathered using atomic resolution IL-(S)TEM bottom-up approach. However, to directly link nanoparticle-specific structure to its experimentally obtained activity, the missing piece of the puzzle remains measuring the activity of the individual particle in question, also referred to as single-entity electrochemistry (Albrecht et al., 2016; Baker, 2018). Although there have been striding steps made toward determining the activity of individual nanoparticles (Kleijn et al., 2014), this methodology is still not ripe and feasible for widespread adoption. Even when combining single-particle activity measurements and stability analysis, given the complexity of structures of individual particles and their operation-induced evolution, a definitive unraveling of the active sites and their activity will remain an elusive goal.

Nevertheless, this is a very important step toward an understanding of not only the activity of any specific active site but even more importantly also its stability. Thus, as exemplified on (for the most part) a single Pt-Co nanoparticle, a lot of further work is needed in order to fully utilize the information one can obtain using atomic resolution IL-(S)TEM bottom-up approach. Extracting this type of information, not only looking at atoms but also using machine-learning approaches for deep analysis, is what our platform enables, and this is in our opinion the next direction in nanoparticle research. As a methodology, it transcends just fuel cells and can be applied to all types of nanostructured electrocatalysts.

## Conclusions

The present manuscript addresses the complexity of the structure of real Pt-alloy PEMFC ORR electrocatalysts both as an experimental case study and through rigorous insight from the published literature and showcases our unique methodological approach that looks into structure-property relationships of individual nanoparticles at the atomic level.

By characterizing four commercial electrocatalysts (Pt-Cu, Pt-Ni, Pt-Co, and Pt-Fe) that have comparable overall characteristic with a set of conventional top-down techniques, we expose that the observed differences in behavior cannot be explained by relying on the averaged-out material properties (e.g. metal loading, Pt/M composition, carbon support, particle size distribution, electrochemical surface area, etc.) and that instead of presuming perfectly shaped model structures, the immense structural complexity of the material must be addressed in order to explain the observed stability behavior and devise strategies for improving it.

The nanoparticles in the studied materials vary in size, shape, crystal ordering, and alloy composition; they have different surface crystal facets, some are covered with carbon shell, some show the presence of twin boundaries, steps, concave sites, etc. These parameters vary not only on average when comparing different alloys but also, crucially, among individual nanoparticles in a given catalyst.

As the atomic architecture of each active site determines its ORR activity and stability (e.g. *via* generalized coordination number), it is inevitably necessary to study structure-property relationships at the atomic level of individual nanoparticles.

For this reason, we introduce a novel bottom-up approach that enables us to study structure-stability relationships at an atomic level. This is possible as we observe and analyze the atomic structure and its changes of the same nanoparticles under atomic-resolution (S)TEM and analyze the differences before and after electrochemical treatment in great detail.

The identical location (S)TEM methodology is combined with image analysis and a modified floating-electrode electrochemical cell that enables performing degradation protocols with high current densities, which mimic fuel cell conditions more closely than the conventional RDE technique. This approach provides fundamental insights into the structural stability behavior of real Pt-alloy electrocatalysts and can thus lead to novel solutions to improve their performance. It also opens a new frontier in nanostructured catalyst stability research that extends beyond Pt-alloy electrocatalysts.

### Limitations of study

- It is still not possible to measure the activity of individual nanoparticle in the arrangement of IL-TEM. This limits the possibility to get insights into the structure-activity relationship on an individual nanoparticle.
- Gaining knowledge and structure-stability relationship on individual nanoparticles is a slow process.
- The electron beam can damage and or change the structures of the nanoparticles.

### Resource availability

#### Lead contact

Further information and requests for resources should be directed to and will be fulfilled by the Lead Contact, Nejc Hodnik, nejc.hodnik@ki.si.

#### Materials availability

This study did not generate any new unique reagents.

#### Data and code availability

Any data utilized in this study can be found in the main manuscript and the Supplemental Information.

## Methods

All methods can be found in the accompanying Transparent methods supplemental file.

## Supplemental information

Document S1. Transparent methods, Figures S1–S28, and Tables S1 and S2

## Footnotes

Supplemental Information can be found online at https://doi.org/10.1016/j.isci.2021.102102.
