# Supplementary material for: Resolving the nanoparticles' structure-property relationships at the atomic level: a study of Pt-based electrocatalysts
Source: iScience. 2021 Jan 28;24(2):102102. doi: 10.1016/j.isci.2021.102102 (PMC7890412; doi:10.1016/j.isci.2021.102102)
Supplement: Document S1. Transparent methods, Figures S1–S28, and Tables S1 and S2 [file mmc1.pdf]

iScience, Volume *24*

Supplemental Information

Resolving the nanoparticles'

structure-property relationships at the atomic

level: a study of Pt-based electrocatalysts

Leonard Jean Moriau, Armin Hrnjić, Andraž Pavlišič, Ana Rebeka Kamšek, Urša Petek, Francisco Ruiz-Zepeda, Martin Šala, Luka Pavko, Vid Simon Šelih, Marjan Bele, Primož Jovanovič, Matija Gatalo, and Nejc Hodnik

Supplemental Information:

TRANSPARENT METHODS

ICP-OES and digestion. All reagents used were of analytical grade or better. For sample dilution and preparation of standards, ultrapure water (Milli-Q, Millipore) and ultrapure acids (HNO_3 and HCl, Merck-Suprapur) were used. Standards were prepared in-house by dilution of certified, traceable, inductively coupled plasma (ICP)-grade single-element standards (Merck CertiPUR). A Varian 715-ES ICP optical emission spectrometer was used. Prior to ICP-OES analysis, each sample was weighed (approximately 10 mg) and digested using a microwave-assisted digestion system (CEM MDS-2000) in a solution of 6 mL HCl and 2 mL HNO_3. The digested samples were cooled to RT and then diluted with 2 %v/v HNO_3 until their concentration was within the desired concentration range. After the digestion procedure, samples were centrifuged to yield clear solutions that were used in subsequent analysis.

XRD analysis. The powder X-ray diffraction (XRD) measurements of all samples were carried out on a Siemens D5000 diffractometer with Cu Kα1 radiation (λ = 1.5406 Å) in the 2θ range from 10° to 60° with the 0.04° step per 1 s. Samples were prepared on zero-background Si holder.

Electrochemical evaluation via Thin Film Rotating Disc Electrode (TF-RDE). *Preparation of thin films and the setup* – Electrochemical measurements were conducted in a two-compartment electrochemical cell in a 0.1 M $HClO_4$ (Merck, Suprapur, 70 %, diluted by Milli-Q, 18.2 MΩ cm) electrolyte with a conventional three-electrode system controlled by a potentiostat (CompactStat, Ivium technologies). Ag|AgCl was used as a reference and a Pt wire as a counter electrode. The working electrode was a glassy carbon disc embedded in Teflon (Pine Instruments) with a geometric surface area of 0.196 cm^2. The Ag|AgCl reference was

separated from both the working and the counter electrode via a salt bridge in order to avoid Cl^- ions contamination. Prior to each experiment, the two-compartment electrochemical cell was boiled in Milli-Q water for 1 hour, and the electrode was polished to mirror finish with Al_2O_3 paste (particle size 0.05 µm, Buehler) on a polishing cloth (Buehler). After polishing, the electrodes were rinsed and ultrasonicated (Ultrasound bath Iskra Sonis 4) in Milli-Q water for 5 min. 20 µL of 1 mg mL^{-1} water-based well-dispersed electrocatalyst ink was pipetted on the glassy carbon electrode completely covering it and dried under ambient conditions. After the drop had dried, 5 µL of Nafion solution (ElectroChem, 5 % aqueous solution) diluted in isopropanol (1:50) was added. Such preparation resulted in the electrocatalyst loading of 20 µg for all four Pt/C (Hi-spec 4000 from Johnson Matthey, Elyst Pt50 0550 from Umicore, TEC10E50E and TEC10E50E-HT from Tanaka Kikinzoku Kogyo) and Pt-M/C electrocatalysts (Fuel Cell Store; M being Cu, Fe, Ni and Co). Each time, at least 4 GC electrodes were prepared and only the best electrocatalyst film was selected for the measurement.

Electrochemical potential cycling activation – After drying, the electrode was mounted on a rotator (Pine Instruments). The electrode was placed in Ar saturated electrolyte under potential control at 0.05 V_{RHE}. All Pt-M/C electrocatalysts were electrochemically activated for 200 cycles between 0.05 and 1.2 V_{RHE} with a scan rate of 300 mV s^{-1} under a rotation rate of 600 rpm (hereinafter referred to as PCA). After PCA, the electrolyte was again exchanged for a fresh one. ORR polarisation curves were measured in an oxygen saturated electrolyte with a rotation rate of 1600 RPM in the potential window 0.05–1.0 V_{RHE} with a scan rate of 20 mV s^{-1}. At the end of ORR polarisation curve measurement, the electrolyte was purged with CO under potentiostatic mode (0.05 V_{RHE}) in order to ensure efficient CO adsorption. Afterwards, the electrolyte was saturated with Ar. CO electrooxidation was performed using the same potential window and scan rate as in ORR, but without rotation and in an Ar saturated electrolyte. Electrochemically active surface area (ECSA) was determined by integrating the charge in CO

electrooxidation experiments as described in reference (Mayrhofer et al., 2008). After subtraction of background current due to capacitive currents, kinetic parameters were calculated at 0.9 V_{RHE}. Ohmic resistance of the electrolyte was determined and compensated for as reported in reference (van der Vliet et al., 2010). In the case no further accelerated degradation tests (ADTs), the electrocatalyst film was suspended in 0.5 mL of isopropanol (removed from the glassy carbon electrode with a few seconds of ultrasonication in an ultrasound bath Iskra Sonis 4) and stored for subsequent *ex-situ* TEM analysis.

Electrochemical accelerated degradation tests – After PCA, each Pt-M/C electrocatalyst was subjected to an ADT (5000 cycles, 0.4–1.2 V_{RHE}, 1 V s^{-1}). Prior to the start of the ADT, the counter electrode was switched for a graphite rod in order to prevent excess Pt dissolution and redeposition from the Pt wire counter electrode on the working electrode. Afterwards, graphite rod was once again switched for a Pt wire and electrolyte was once again replaced with a fresh one. ORR polarisation curves (including ohmic resistance and compensation) and CO stripping measurements were repeated using the same process as described during PCA. For each Pt-M/C electrocatalyst, the ADT was performed on at least 3 films after PCA. The electrocatalyst film was at the end suspended in 0.5 mL of isopropanol (removed from the glassy carbon electrode with a few seconds of ultrasonication in an ultrasound bath Iskra Sonis 4) and stored for subsequent *ex-situ* TEM analysis.

EFC-ICP-MS. *Electrochemical Flow Cell Setup* – The working and counter electrode in the electrochemical flow cell (EFC) were glassy carbon discs (3 mm diameter) embedded into PEEK material (BASi). The discs were aligned in series; the counter electrode was placed first and the working electrode second in the direction of the electrolyte flow. The electrocatalysts were deposited on the electrode by drop casting 5 µL drop of the ultrasonically homogenised catalyst ink (1 mg mL^{-1}). Such preparation resulted in the electrocatalyst loading of 5 µg for all Pt-M/C electrocatalysts. After the drop had dried, 5 µL of Nafion solution (ElectroChem, 5 %

aqueous solution) diluted in isopropanol (1:50) was added. The Ag|AgCl reference electrode potential against RHE was determined before the start of the experiment. The housing of the cell was made from PEEK material and the design was modelled after a commercial cross-flow cell (BASi, MF-1092, cross-flow cell). The volume of the cell was established with a home-made silicon gasket with 1.0 mm thickness and 1.5 cm^2 ellipsoidal cut. The carrier solution (0.1 M $HClO_4$) was pumped through the cell at a constant flow of 400 μL min^{-1}. Two 25 mL glass syringes (PTFE LUER-LOCK GT SYRINGE; Thermo Fischer), two syringe pumps (WPI AL1000-220Z) and a diagonal 4-way flow valve (Idex, V-100D) were used to enable a continuous flow of the solution.

ICP-MS – The EFC was coupled with an ICP-MS detector, namely Agilent 7900ce ICP-MS instrument (Agilent Technologies, Palo Alto, CA), equipped with a MicroMist glass concentric nebulizer and a Peltier cooled Scott-type double-pass quartz spray chamber. A forward radio-frequency power of 1500 W was used with Ar gas flows: carrier 0.85 L min^{-1}; makeup 0.28 L min^{-1}; plasma 1 L min^{-1}; and cooling 15 L min^{-1}. The signals were recorded for Fe^{56}, Co^{59}, Ni^{60}, Cu^{63}, and Pt^{195} with 0.5 s integration per data point. To convert the ICP-MS signals to concentration (ppb), a standard solution of Cu, Fe, Ni, Co and Pt in 0.1 M $HClO_4$ were recorded with the following concentrations: 1, 2, 5, 10, 20, 50 and 100 ppb.

Electrochemical protocol – Electrochemical experiments were performed with an Ivium potentiostat (CompactStat, Ivium technologies) with a typical three-electrode setup. No ohmic drop compensation method was used. Initially, Milli-Q water was pumped through the cell before switching to the 0.1 M $HClO_4$ in order to notice any dissolution due to acid contact. After reaching a stable background level, in one set of experiments, slow potential cycles from 0.05 V_{RHE} to an increasing upper-potential limit (1.0, 1.2 and 1.4 V_{RHE}) were performed to as-purchased electrocatalysts. Two cycles were repeated in each potential window. In another set of experiments, the electrocatalysts were first electrochemically activated (PCA; 200 cycles

between 0.05 and 1.2 V_{RHE} with a scan rate of 300 mV s^{-1}). Afterwards, as in the case of the first set of experiments, slow potential cycles from 0.05 V_{RHE} to an increasing upper-potential limit (1.0, 1.2 and 1.4 V_{RHE}) were performed as well in the same manner. In both sets of experiments, a sequence of potential pulses was performed in order to synchronise the electrochemical experiment with the ICP-MS signal.

Electrochemistry – Mass Spectrometry (EC-MS) for carbon degradation. *Electrochemistry – mass spectrometry apparatus* (SpectroInlets) consists of a mass spectrometer with a quadrupole mass analyser (Pfeiffer Vacuum, PrismaPro QMG 250 M2) and a specially designed thin-layer three-electrode electrochemical cell, controlled with a potentiostat (BioLogic, SP-300). A porous chip serves as an interface between the electrolyte in the electrochemical cell and the vacuum system of the MS. The cell is assembled on top of the chip in such a way that the working electrode (glassy carbon disk, 5 mm diameter, Pine) is put in parallel with the porous chip at a constant distance of 100 μm. The spacing is filled with electrolyte (0.1 M $HClO_4$, Carl Roth, ROTIPURAN, diluted with Milli-Q) and connected to compartments containing a reference (Ag/AgCl, 3 M NaOH, BASi) and counter electrode (graphite rod, Alfa Aesar, 99.9995% purity). Volatile species that form at the working electrode diffuse through the electrolyte layer, equilibrate across the membrane chip and are then picked up by a carrying gas (He 5.0) before being detected by the quadrupole analyser. The carrying gas also deaerates the electrolyte layer. Several *m*/*z* signals were recorded, namely 2, 4, 28, 32, 44, 30, and 46, corresponding to H_2, He, N_2/CO, O_2, CO_2, NO and NO_2 ionized fragments. The temporal MS resolution was 1.5 s and the data were precisely timed with the electrochemical experiment.

Sample preparation. Suspensions of electrocatalysts (1 mg mL^{-1}) were prepared in Milli-Q, sonicated in an ultrasound bath for 10 min, deposited on the glassy carbon disk (10 μL) and

dried in air in order to prepare a homogeneous sample film (51 μg cm^{-2}). Prior to assembling the cell, the film was wetted with a drop of 0.1 M $HClO_4$.

Electrochemical protocol. Due to the resistive nature of the cell design, the applied electrochemical protocols had to be adjusted from those used in the EFC-ICP-MS system in order to enable control of the cell. In all measurements, the bandwidth parameter was set to 1. The activation protocol was carried out in the same potential range as PCA (0.05–1.2 V_{RHE}), but at a slower scan rate (150 mV s^{-1}, 100 cycles). To remove the dissolved M ions, the electrolyte in the cell was exchanged following the activation protocol with fresh 0.1 M $HClO_4$ before further tests. Instead of AST, sets of 20 cycles in potential ranges with increasing UPL were carried out (0.40–1.X V_{RHE}; X = 2, 3, 4, 5; 100 mV s^{-1}), separated by 5 minutes of open-circuit potential.

It should be noted that in cases with high Pt loading (Pt/C electrocatalysts), a signal for H_2 was detected during the activation protocol (data not shown), which was attributed to instabilities in the potentiostat–cell system, causing oscillations at the low-potential limit.

TEM analysis. Scanning transmission electron microscopy was performed in a JEOL ARM 200 CF equipped with a SSD Jeol EDX spectrometer. The instrument was operated at 80 kV, and imaging with an estimated current density of 14.5 pA using 68-175 mrad of inner and outer angles of the detector.

Ex-situ TEM analysis – for *ex-situ* TEM analysis of as-purchased Pt-M/C electrocatalysts, 50 µL of 1 mg mL^{-1} suspension was diluted in 0.5 mL of Milli-Q water and 0.45 mL of isopropanol. After brief ultrasonication of the diluted suspension in the ultrasound bath (Iskra Sonis 4), 5 µL of the suspension was pipetted on the TEM grid and dried under ambient conditions. For *ex-situ* TEM analysis of Pt-M/C electrocatalysts after PCA and ADT, also 5 µL of the Pt-M/C suspensions in isopropanol that were collected from the GC electrodes were pipetted on the TEM grid and dried under ambient conditions. Pt-Ni/C, Pt-Fe/C and Pt-Co/C were pipetted on

copper TEM grids, while Pt-Cu/C electrocatalyst was pipetted on a nickel TEM grid to avoid the influence of M during EDX measurements.

Modified floating electrode (MFE). The working electrode compartment was made of two-piece Teflon housing, which was assembled with Tekka Peek screws. Between these elements of the housing, a metallic spring, two metallic cones, a gas diffusion layer (GDL, 280 µm thickness) with 40% Teflon weight wet proofing (Toray Carbon Paper 090, Fuel Cell Store) and gold TEM grid working electrode (Agar Scientific, Holey Carbon Films on 300 Mesh Gold) were inserted. The TEM grid was inserted below the GDL, whereas the spring and two metallic cones were pressed above the GDL as described in our prior work (Hrnjić et al., 2020). We note that the Teflon in GDL gave the carbon material a hydrophobic property, which prevented the potentially corrosive electrolyte from penetrating and creeping to the metallic cone and spring contacts – hence preventing their damage or even dissolution and thus poisoning of the sample. The GDE, therefore, served as a spacer to separate the working electrode and metallic cones and spring. All three served as an electrical contact for the gold TEM grid working electrode. A gas purging tube was connected to one of the holes of the peek housing *via* plastic tubing.

Catalyst film preparation – commercially obtained catalyst composed of platinum cobalt alloy nanoparticles dispersed on carbon support was investigated: Fuel Cell Store Pt-Co (1:1 molar ratio) supported on carbon, hereinafter referred to as Pt-Co/C. The ink composition consisted of the following ratios of catalyst mass (mg) vs. solvent volume (mL) 1:1. Solvent composition in terms of volume fractions was set as follows: (75% propane-2-ol, 25% Milli-Q water and 1.33% Nafion® (ElectroChem, 5 wt. % aqueous solution). The ink was ultrasonicated (Ultrasound bath Iskra Sonis 4) for 30 min prior to drop-casting. Catalyst ink was deposited on a TEM grid by drop-casting with a syringe (Hamilton), resulting in the Pt loading of 0.9 μg_{Pt} cm^{-2}_{geo}. The catalyst film was dried on a heating stage. Drop-casting was performed on Au side

of the grid. The other side of the grid was coated with a very thin carbon film. The results shown were obtained by the Au side facing the electrolyte.

Identical location transmission electron microscopy characterisation (IL-TEM) in combination with MFE electrochemical characterisation – TEM was operated at 80 kV with a camera length of 8 cm, meaning 68-175 mrad of inner and outer angles of the detector. The used probe current was around 14.5 pA. Once coated with the catalyst, the grid was inspected under TEM. Several spots were identified and imaged in TEM and STEM at different magnifications. After the initial TEM analysis, the grid was removed from the microscope and placed into the MFE assembly to perform the electrochemical treatment. A two-compartment (H-type), Teflon based 10 mL electrochemical cell was used. A platinum mesh counter electrode (GoodFellow 50 × 50 mm) and a reversible hydrogen reference electrode (HydroFlex®) were both placed in the same compartment. In a separate compartment, a modified floating electrode housing was placed. The two compartments were separated by a Nafion membrane (Nafion 117, Fuel Cell Store). A 4 M perchloric acid ($HClO_4$, Merck, Suprapur, 70 %, diluted by Milli-Q, 18.2 MΩ cm) was used as the working electrolyte, as typically used in similar electrode configurations to reduce the ionic resistance of the electrolyte.(Martens et al., 2018; Zalitis et al., 2013) The following electrochemical protocol was used: Initially, a typical electrochemical pre-treatment (PCA) was performed in order to obtain a stable cyclic voltammogram (CV). This consisted of a potentiodynamic perturbation by cycling the potential between 0.05 and 1.2 V vs. RHE (200 cycles, 300 mV s^{-1}) under N_2 atmosphere. Afterwards, N_2 was replaced by O_2 and ORR polarisation curves with ohmic drop compensation were recorded at 300 mV s^{-1} and 20 mV s^{-1}. iR resistance between the working and reference electrode was determined using the high-frequency intercept of an impedance scan for each electrode (measured at open circuit potential). Ohmic drop compensation (85 %) was applied during electrochemical experiments *via* a positive feedback mode. Afterwards, the

TEM grid was dipped into fresh Milli-Q water and left to dry at room temperature. Once dried, the grid was again inspected under TEM, tracking the areas previously identified as locations of interest and imaged for analysis. The images were then taken under the same conditions as described above.

Strain analysis – For the present study, strain maps were obtained with StatSTEM developed by De Backer and co-workers.(De Backer et al., 2017) The algorithm uses Gaussian functions to fit atomic column positions. The giving displacements are then obtained by comparing the measured column positions with those of an ideal lattice column positions, the strain maps are computed from the first derivatives afterwards. To reduce the external influence from scanning acquisition when calculating strain, scan artefacts, known as non-linear distortions, were corrected (**Figure 11a** of the main manuscript**)** using orthogonal scan pairs with an algorithm developed by C. Orphus and co-workers.(Ophus et al., 2016) The software uses Gaussian functions to fit atomic column positions. The giving displacements are then obtained by comparing the measured column positions with those of an ideal lattice column positions, the strain maps are computed from the first derivatives afterwards.

DATA

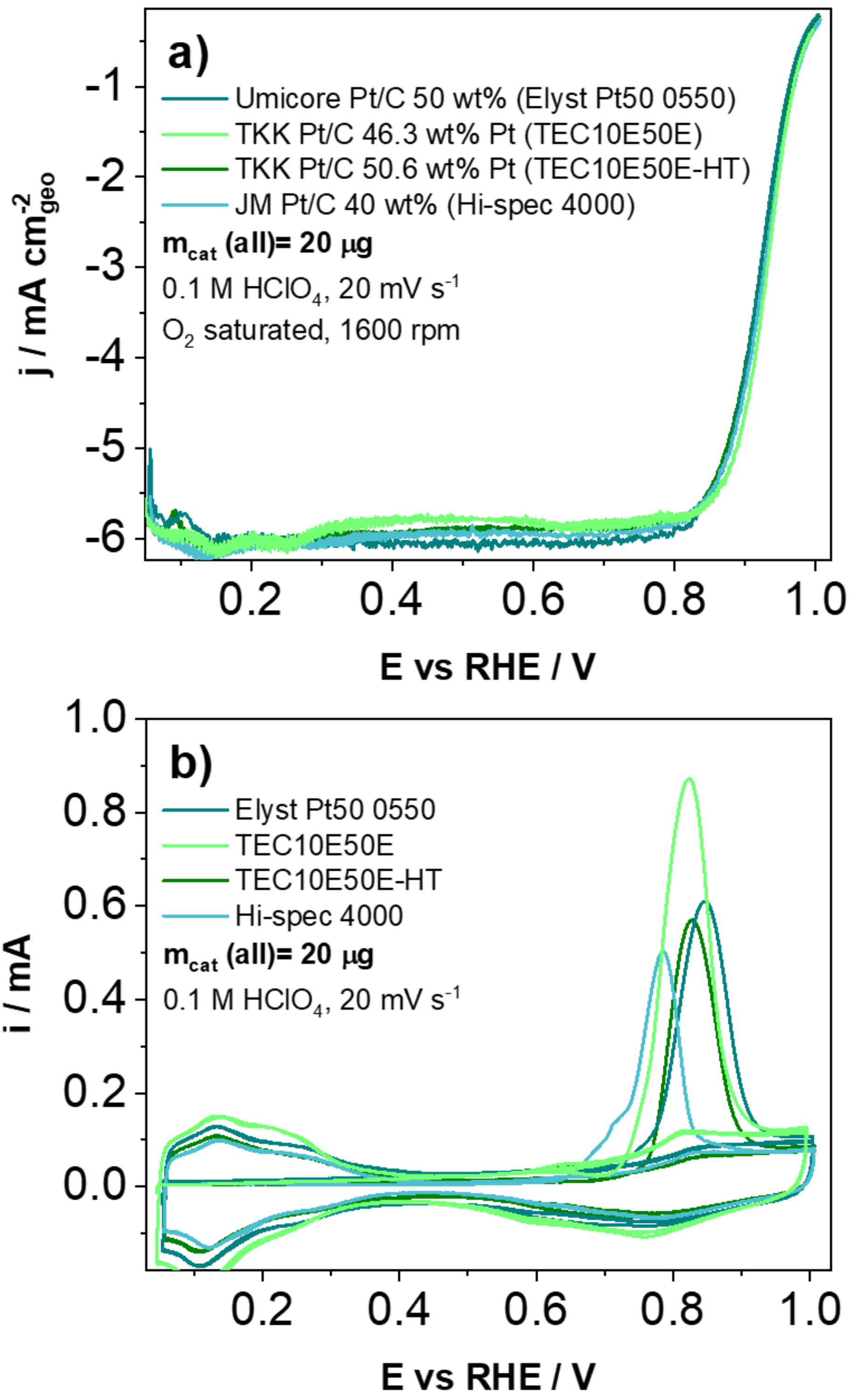

Figure S1. (a) ORR polarisation curves and **(b)** CO-electrooxidation CVs of various commercially available Pt/C electrocatalysts. The TF-RDE data is presented in **Table S1**.

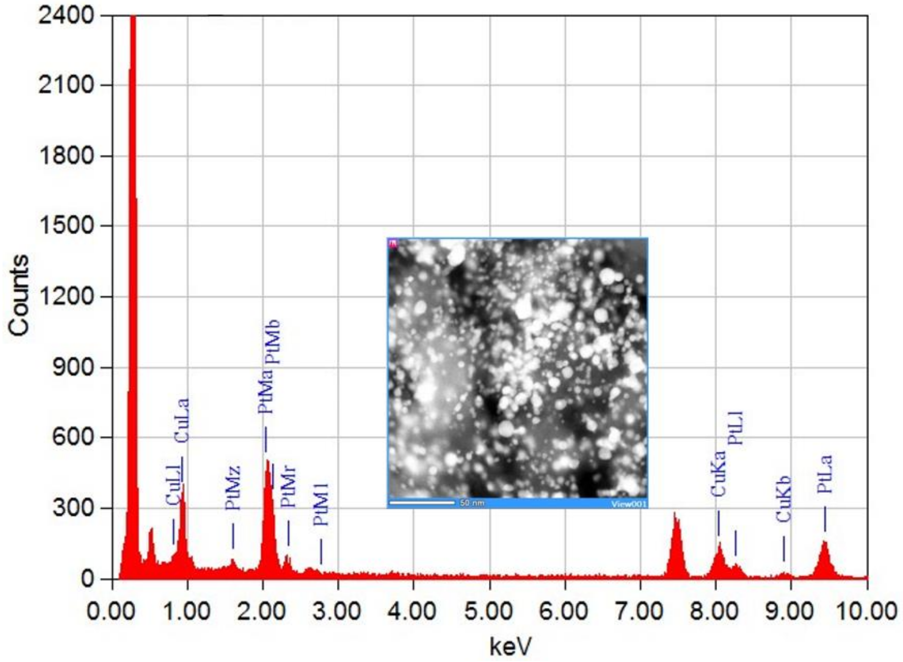

Figure S2. EDX-TEM energy spectra with HAADF image of one of the analysed areas of as-purchased Pt-Cu/C (FCS) electrocatalyst.

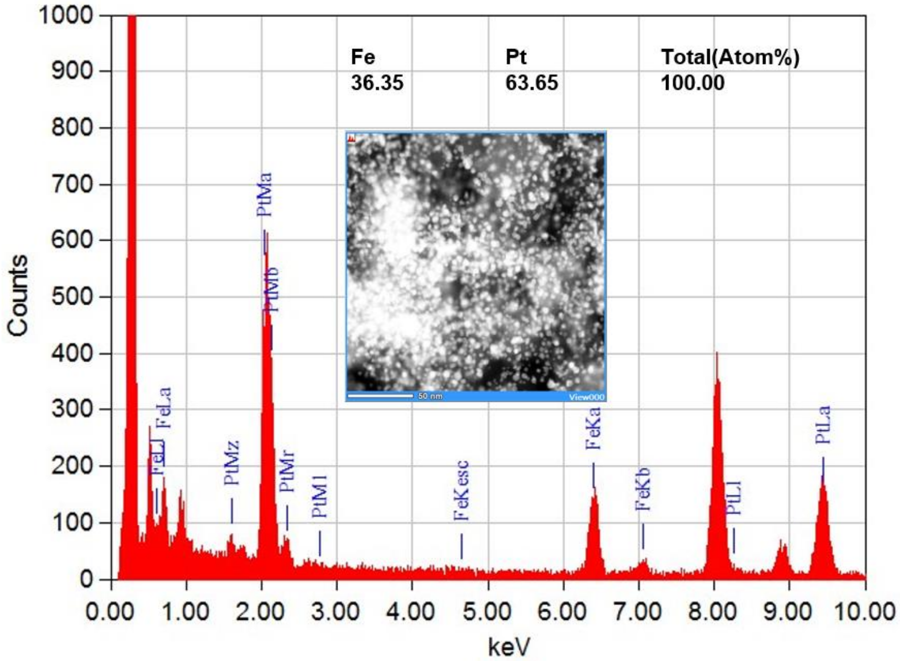

Figure S3. EDX-TEM energy spectra with HAADF image of one of the analysed areas of as-purchased Pt-Fe/C (FCS) electrocatalyst.

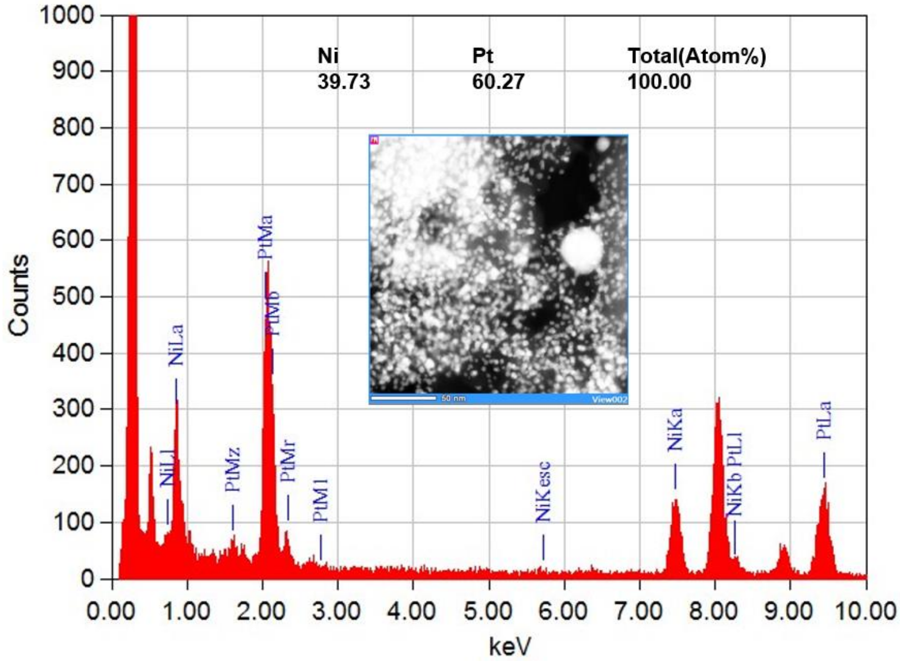

Figure S4. EDX-TEM energy spectra with HAADF image of one of the analysed areas of as-purchased Pt-Ni/C (FCS) electrocatalyst.

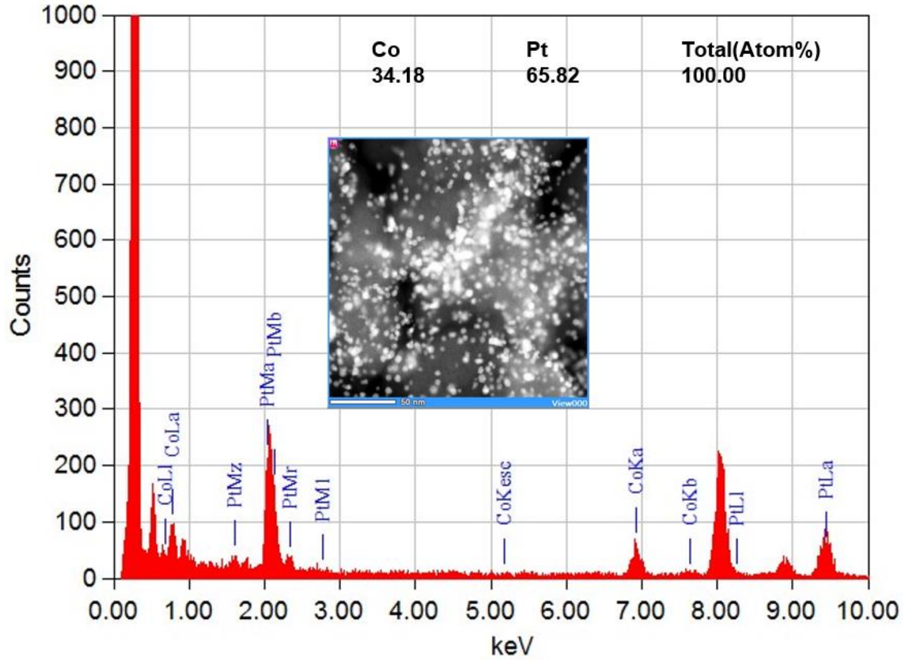

Figure S5. EDX-TEM energy spectra with HAADF image of one of the analysed areas of as-purchased Pt-Co/C (FCS) electrocatalyst.

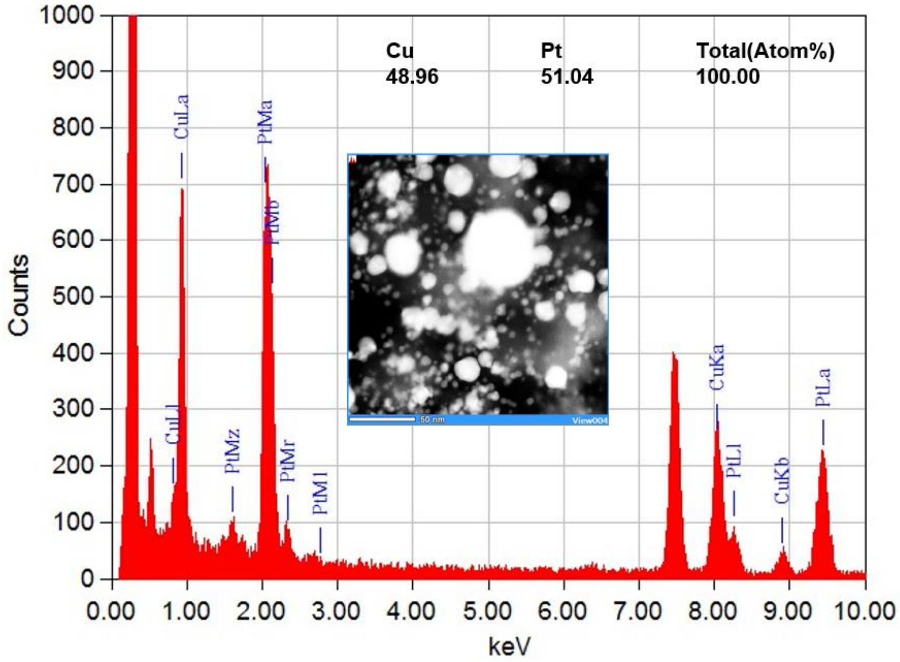

Figure S6. EDX-TEM energy spectra with HAADF image of one of the analysed Cu-richer areas of as-purchased Pt-Cu/C (FCS) electrocatalyst.

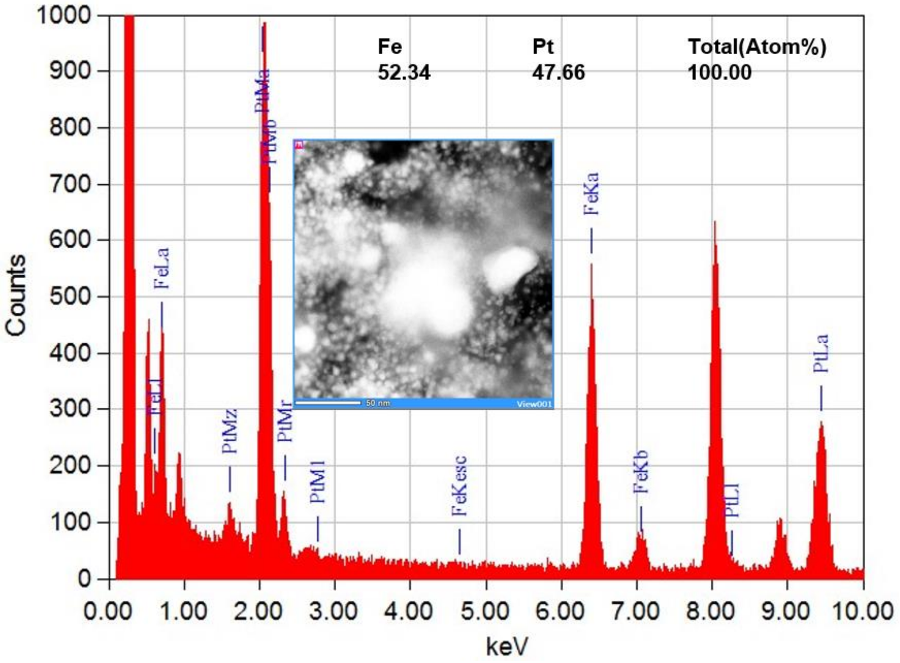

Figure S7. EDX-TEM energy spectra with HAADF image of one of the analysed Fe-richer areas of as-purchased Pt-Fe/C (FCS) electrocatalyst.

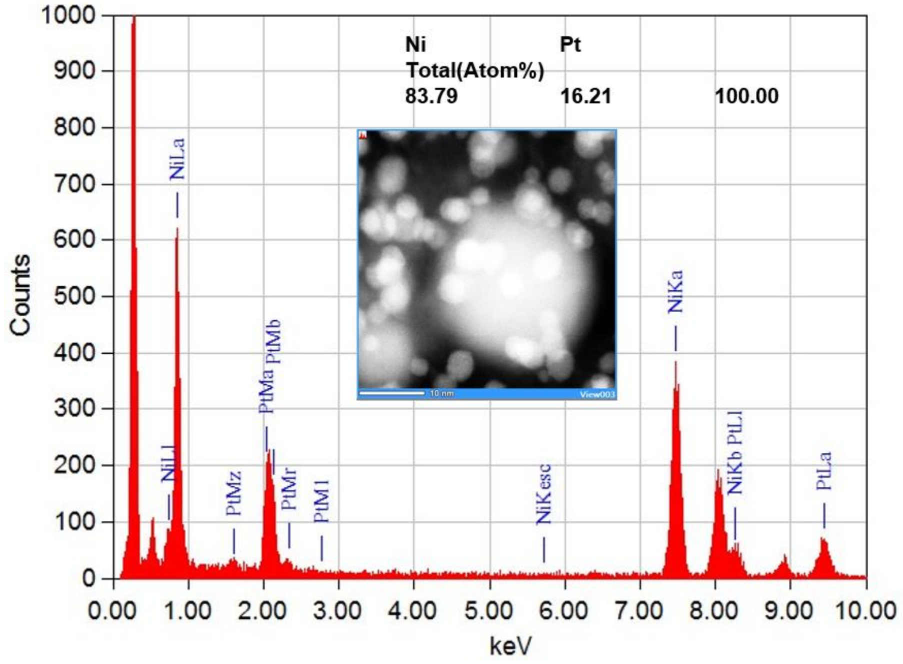

Figure S8. EDX-TEM energy spectra with HAADF image of one of the analysed Ni-richer areas of as-purchased Pt-Ni/C (FCS) electrocatalyst.

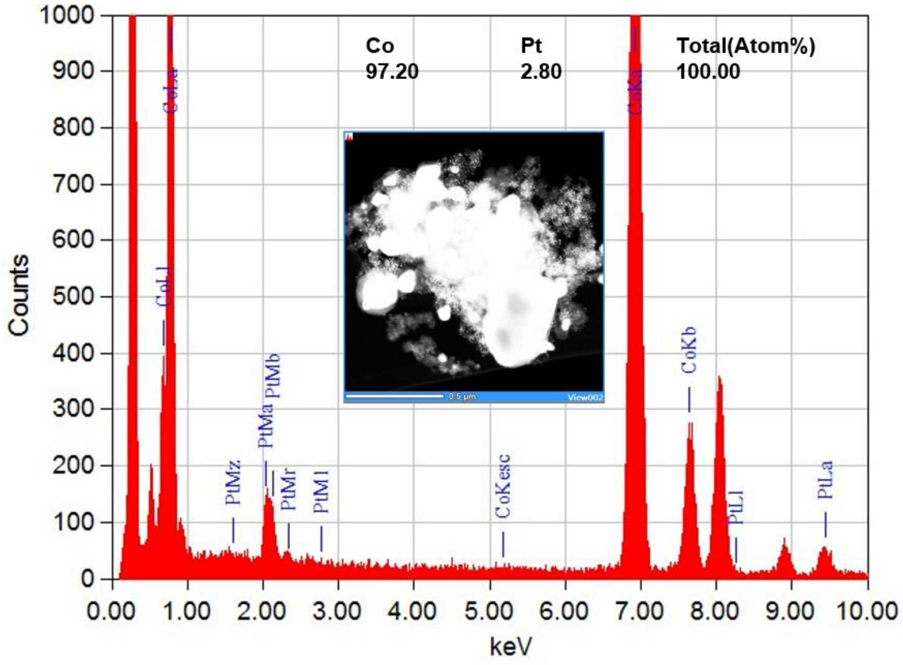

Figure S9. EDX-TEM energy spectra with HAADF image of one of the analysed Co-richer areas of as-purchased Pt-Co/C (FCS) electrocatalyst.

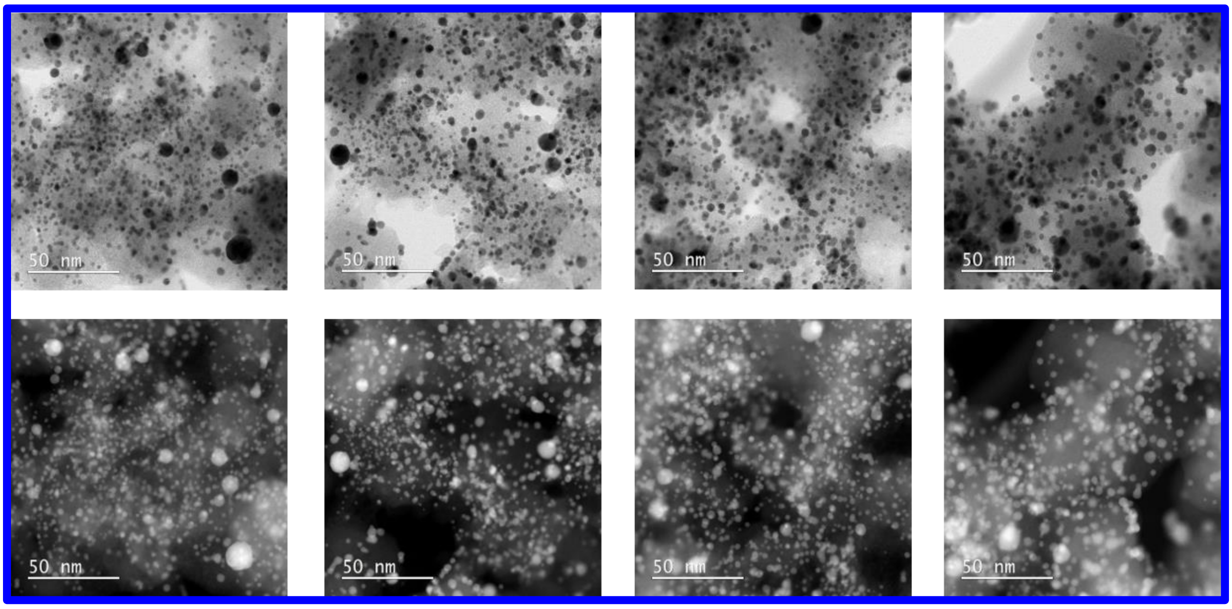

Figure S10. STEM BF and HAADF imaging of Pt-Cu/C (FCS) electrocatalyst after PCA (200 cycles in 0.1 M $HClO_4$, 0.05–1.2 V_{RHE}, 300 mV s^{-1}).

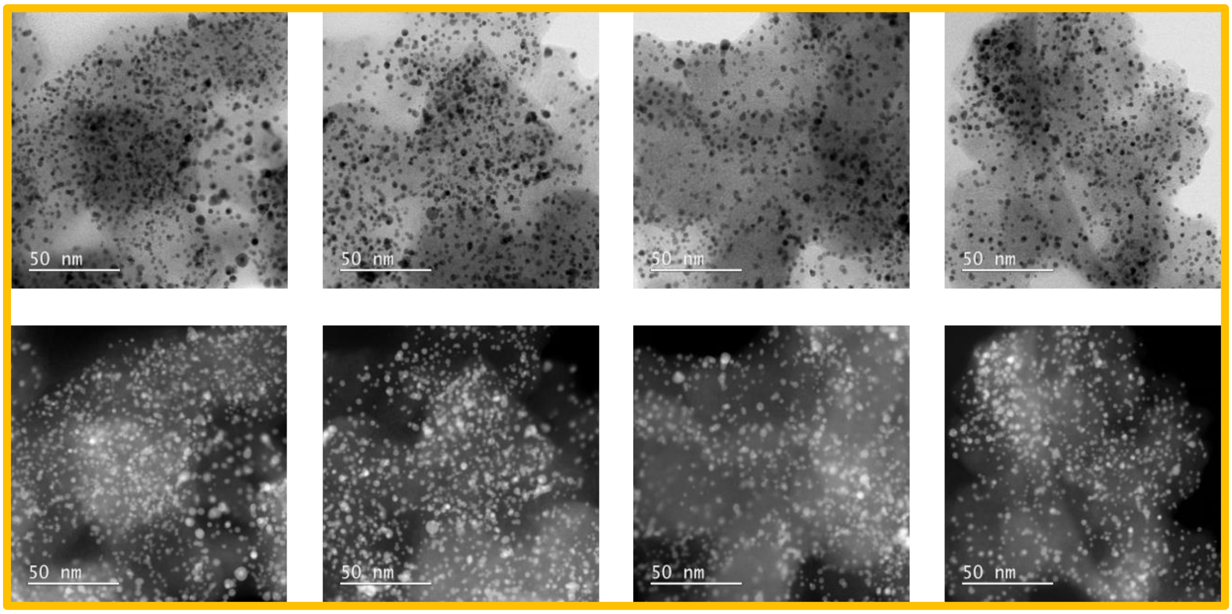

Figure S11. STEM BF and HAADF imaging of Pt-Fe/C (FCS) electrocatalyst after PCA (200 cycles in 0.1 M $HClO_4$, 0.05–1.2 V_{RHE}, 300 mV s^{-1}).

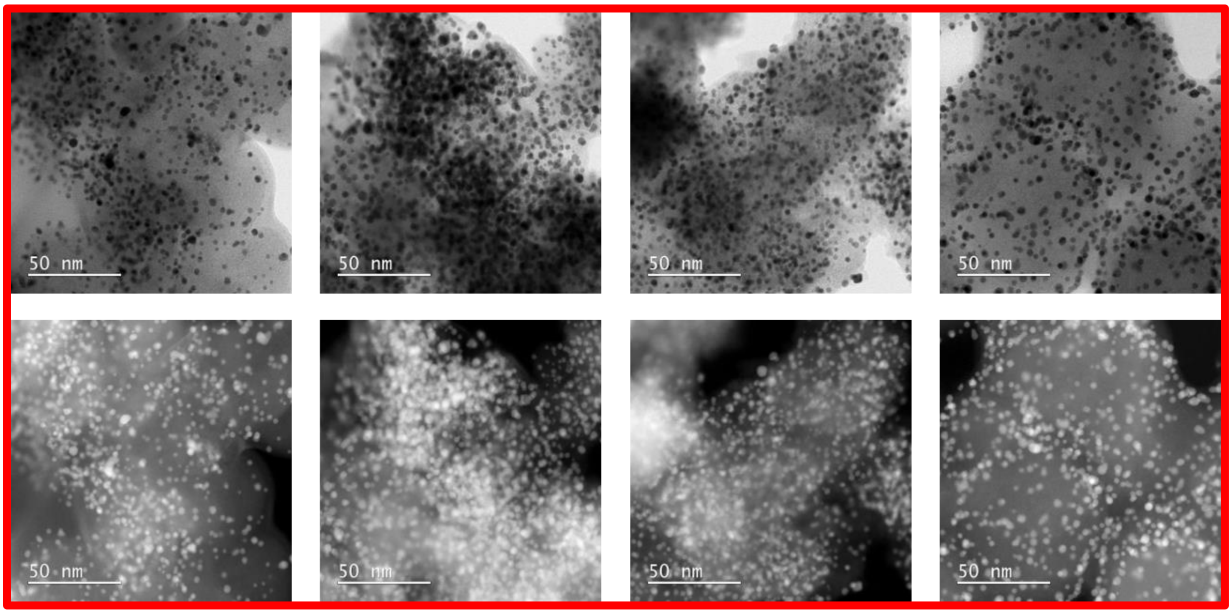

Figure S12. STEM BF and HAADF imaging of Pt-Ni/C (FCS) electrocatalyst after PCA (200 cycles in 0.1 M $HClO_4$, 0.05–1.2 V_{RHE}, 300 mV s^{-1}).

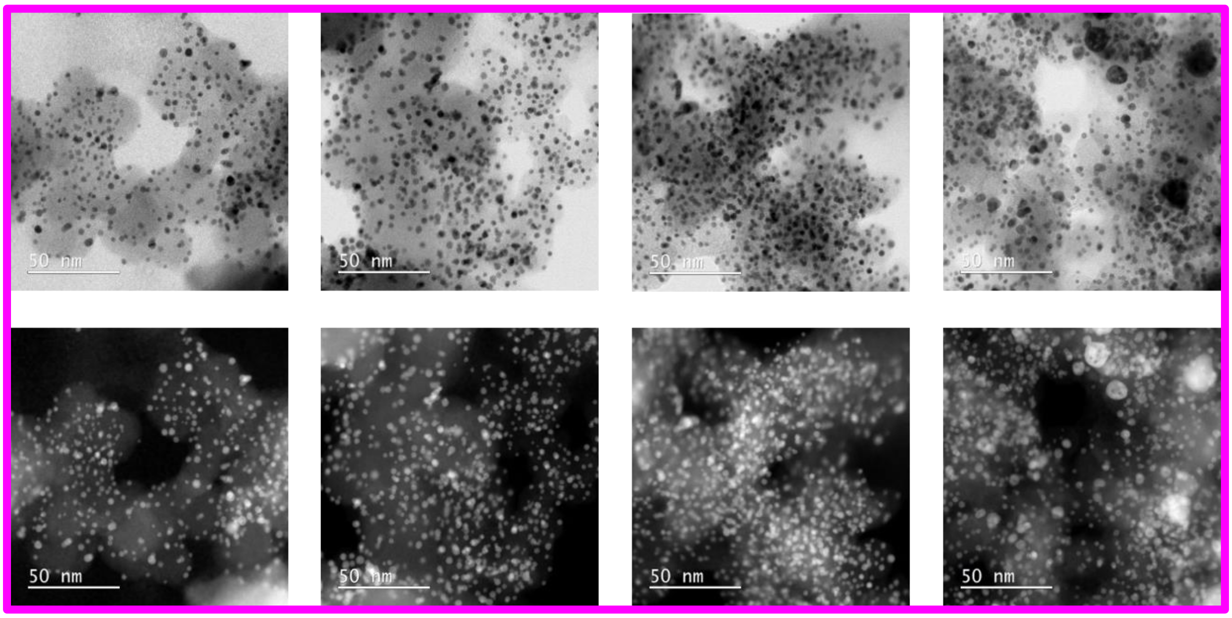

Figure S13. STEM BF and HAADF imaging of Pt-Co/C (FCS) electrocatalyst after PCA (200 cycles in 0.1 M $HClO_4$, 0.05–1.2 V_{RHE}, 300 mV s^{-1}).

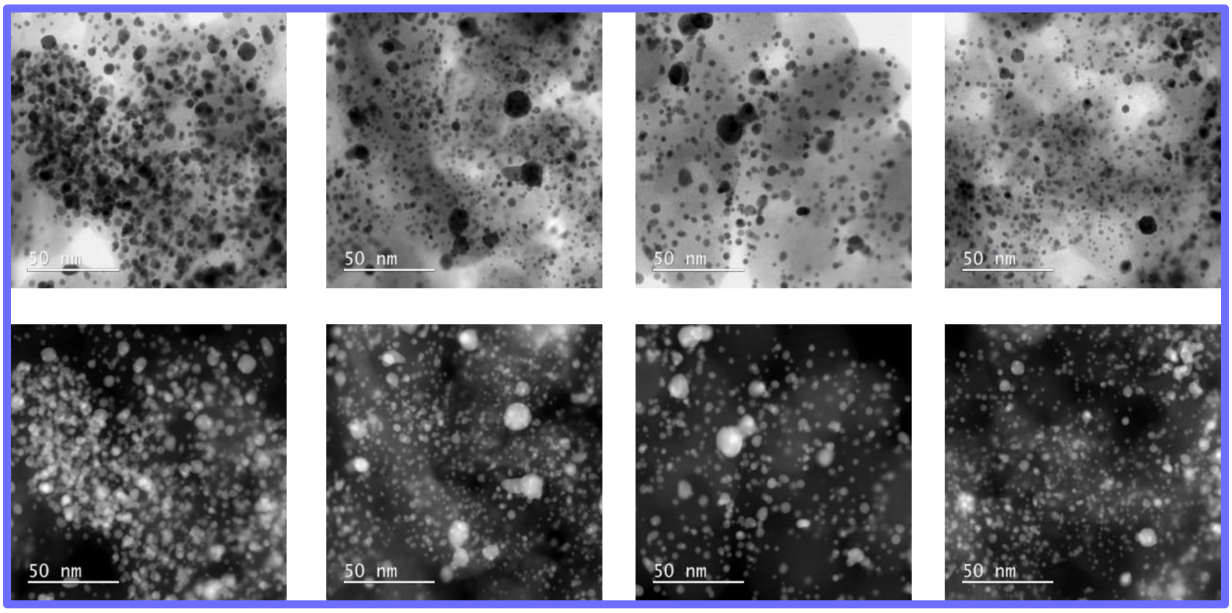

Figure S14. STEM BF and HAADF imaging of Pt-Cu/C (FCS) electrocatalyst after additional ADT (5000 cycles in 0.1 M $HClO_4$, 0.4–1.2 V_{RHE}, 1 V s^{-1}).

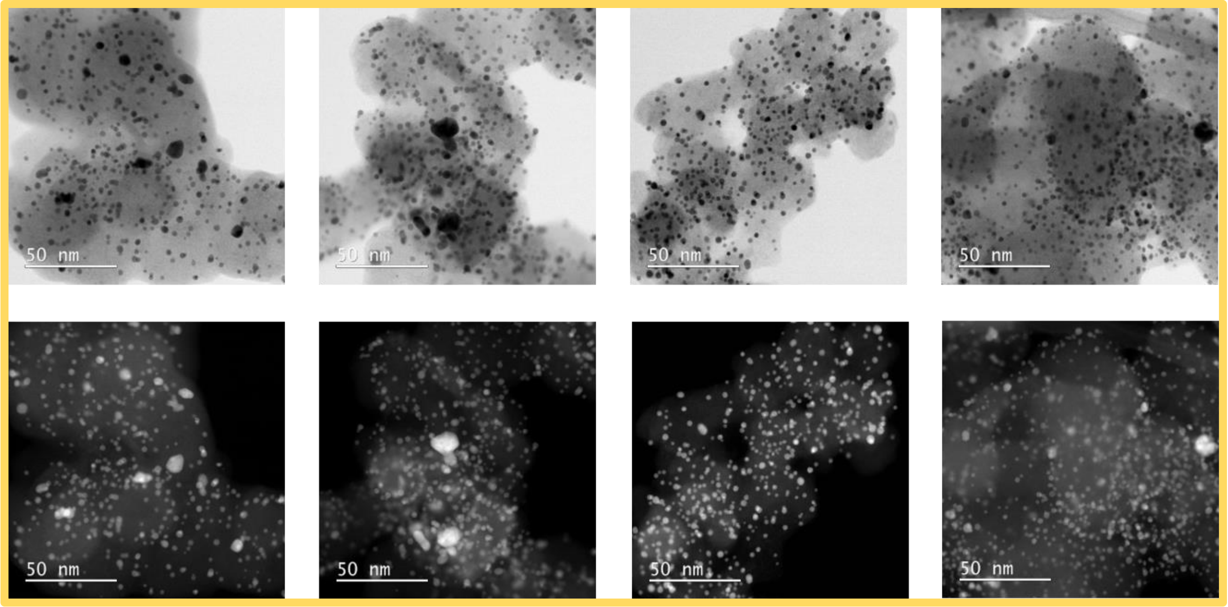

Figure S15. STEM BF and HAADF imaging of Pt-Fe/C (FCS) electrocatalyst after additional ADT (5000 cycles in 0.1 M $HClO_4$, 0.4–1.2 V_{RHE}, 1 V s^{-1}).

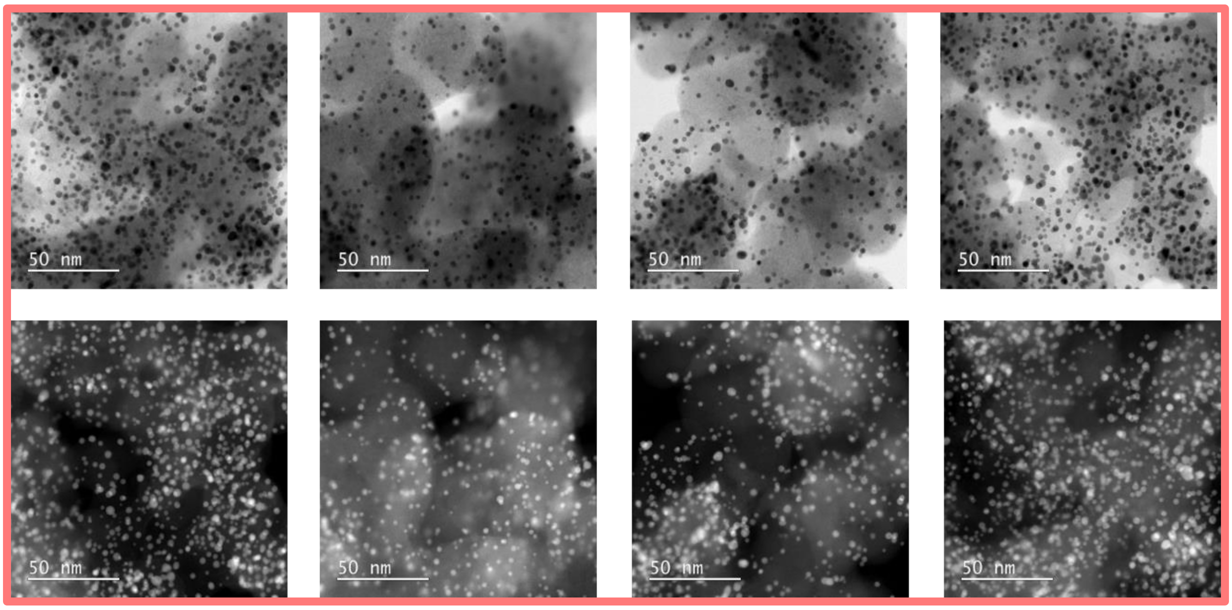

Figure S16. STEM BF and HAADF imaging of Pt-Ni/C (FCS) electrocatalyst after additional ADT (5000 cycles in 0.1 M $HClO_4$, 0.4–1.2 V_{RHE}, 1 V s^{-1}).

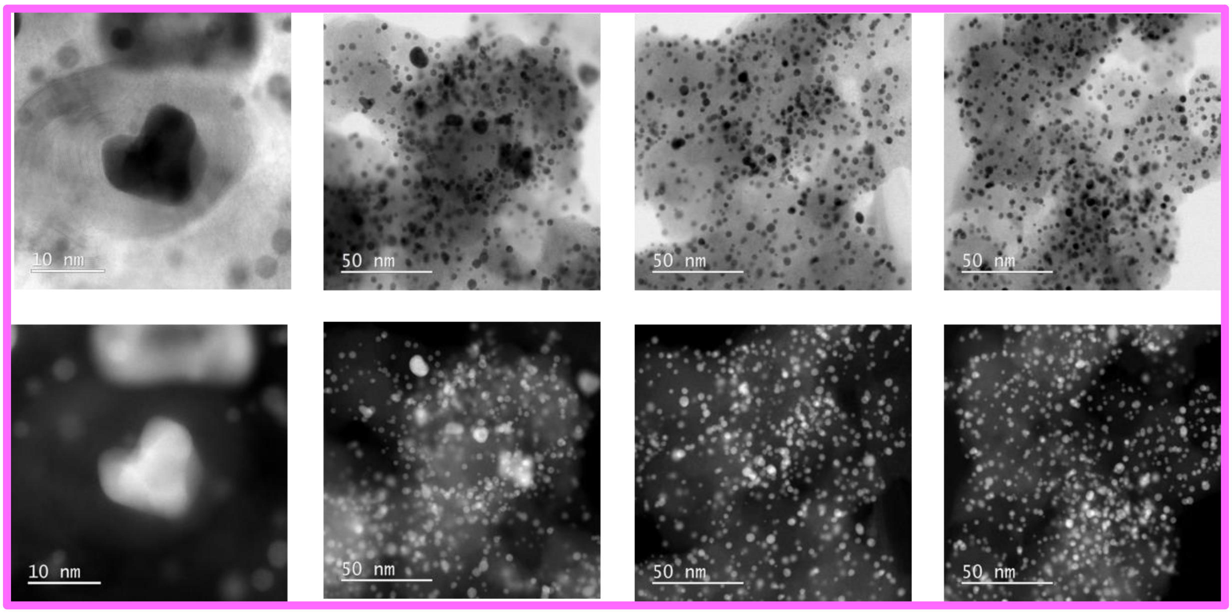

Figure S17. STEM BF and HAADF imaging of Pt-Co/C (FCS) electrocatalyst after additional ADT (5000 cycles in 0.1 M $HClO_4$, 0.4–1.2 V_{RHE}, 1 V s^{-1}). First image on the left shows a heart-shaped nanoparticle surrounded with a spiral-shaped carbon nanotube.

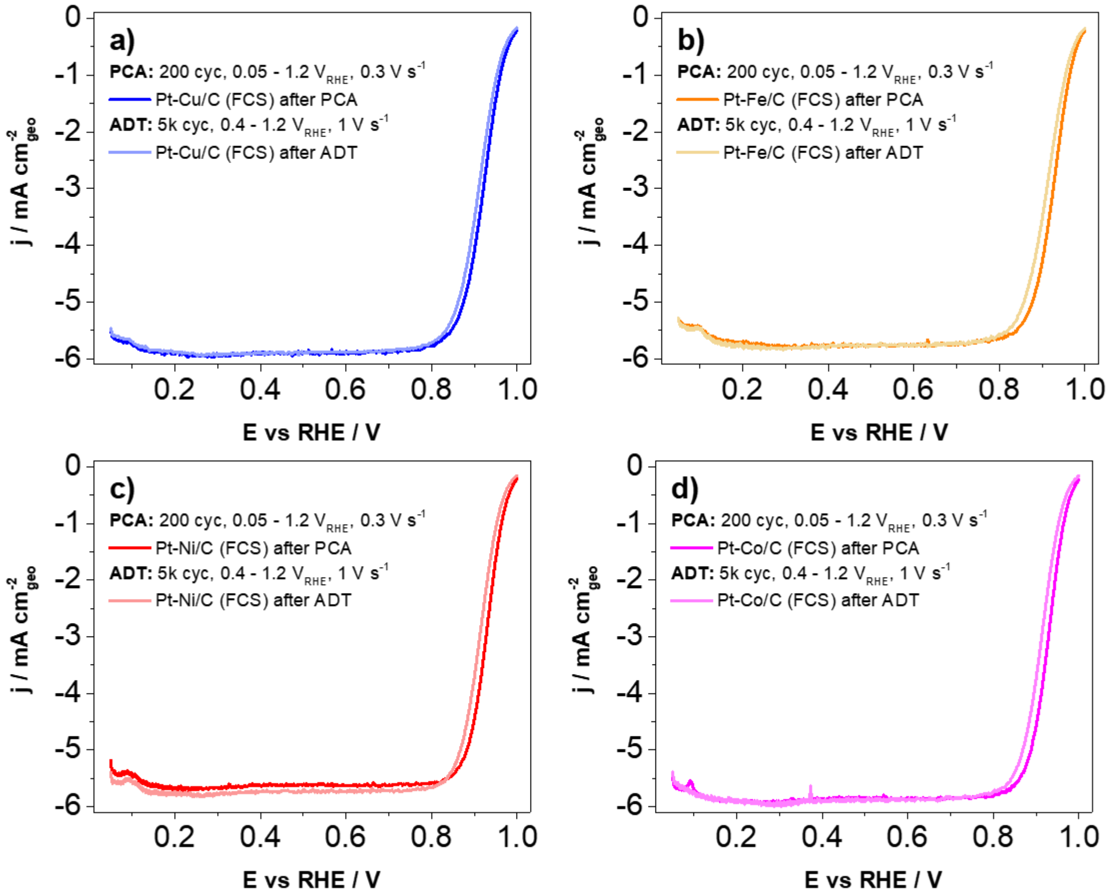

Figure S18. Comparison of ORR polarisation curves (0.1 M $HClO_4$, background corrected, IR compensated, 1600 rpm, O_2 saturated) after PCA (200 cycles in 0.1 M $HClO_4$, 0.05–1.2 V_{RHE}, 300 mV s^{-1}) and after an additional ADT (5000 cycles in 0.1 M $HClO_4$, 0.4–1.2 V_{RHE}, 1 V s^{-1}) for **(a)** Pt-Cu/C, **(b)** Pt-Fe/C, **(c)** Pt-Ni/C and **(d)** Pt-Co/C.

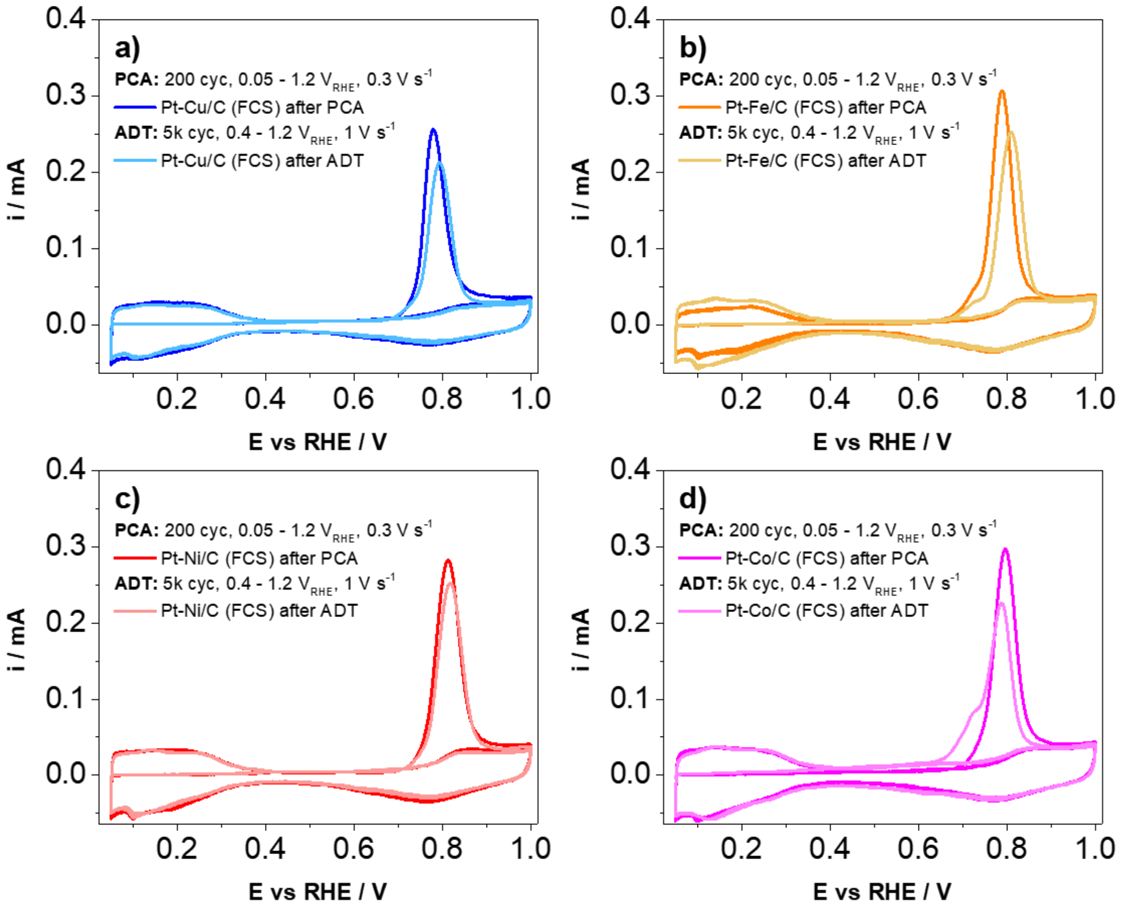

Figure S19. Comparison of CO electrooxidation experiments (0.1 M $HClO_4$, no rotation, Ar saturated) after PCA (200 cycles in 0.1 M $HClO_4$, 0.05–1.2 V_{RHE}, 300 mV s^{-1}) and after an additional ADT (5000 cycles in 0.1 M $HClO_4$, 0.4–1.2 V_{RHE}, 1 V s^{-1}) for **(a)** Pt-Cu/C, **(b)** Pt-Fe/C, **(c)** Pt-Ni/C and **(d)** Pt-Co/C.

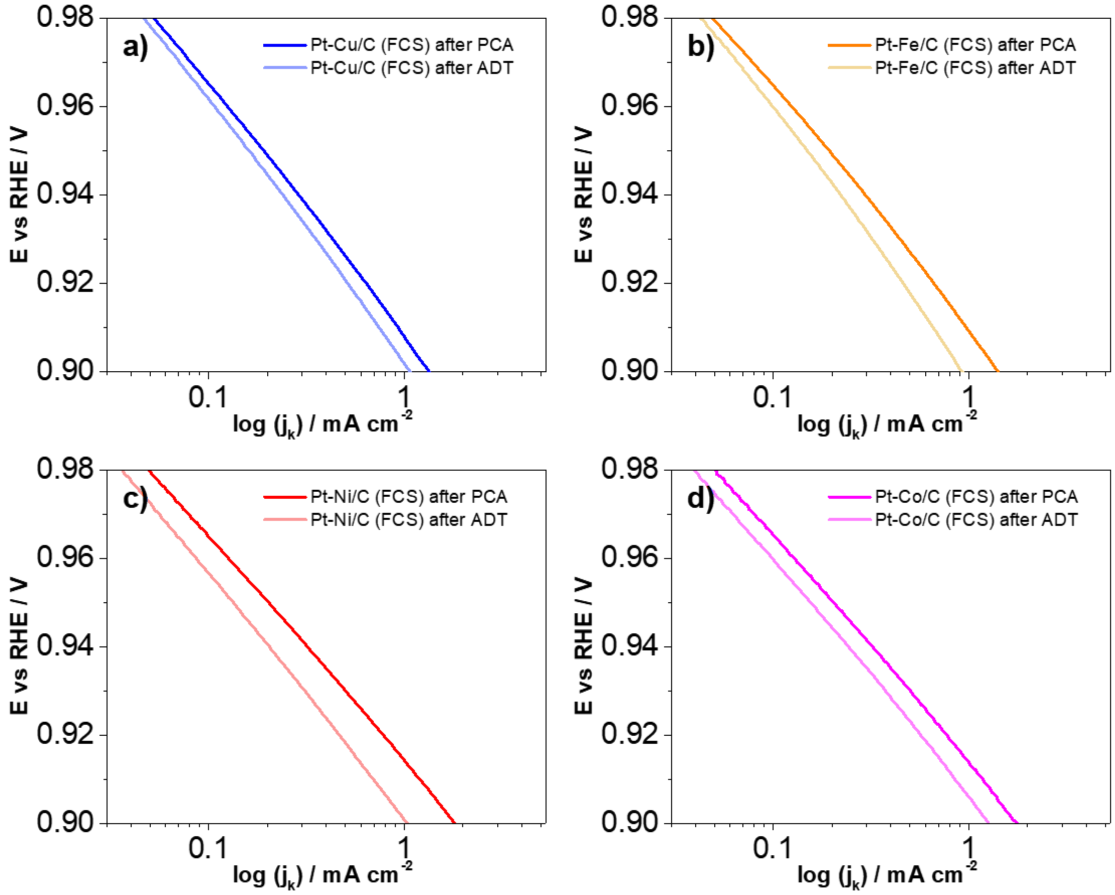

Figure S20. Comparison of Tafel plots (0.1 M $HClO_4$, background corrected, IR compensated, 1600 rpm, O_2 saturated) after PCA (200 cycles in 0.1 M $HClO_4$, 0.05–1.2 V_{RHE}, 300 mV s^{-1}) and after an additional ADT (5000 cycles in 0.1 M $HClO_4$, 0.4–1.2 V_{RHE}, 1 V s^{-1}) for **(a)** Pt-Cu/C, **(b)** Pt-Fe/C, **(c)** Pt-Ni/C and **(d)** Pt-Co/C.

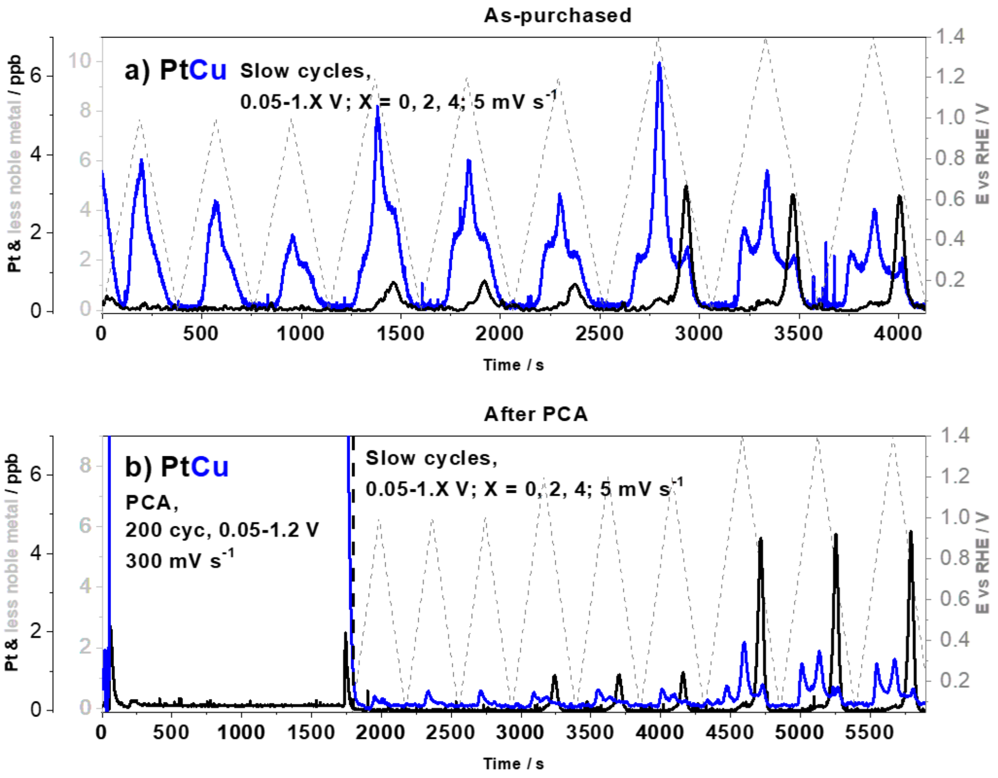

Figure S21. Comparison of EFC-ICP-MS measurements of Pt and Cu dissolution (slow cycles of 5 mV s^{-1} between 0.05–1.X V_{RHE}; X = 0, 2, 4) for Pt-Cu/C (FCS) electrocatalyst in **(a)** as-purchased state and **(b)** after PCA (200 cycles in 0.1 M $HClO_4$, 0.05–1.2 V_{RHE}, 300 mV s^{-1}).

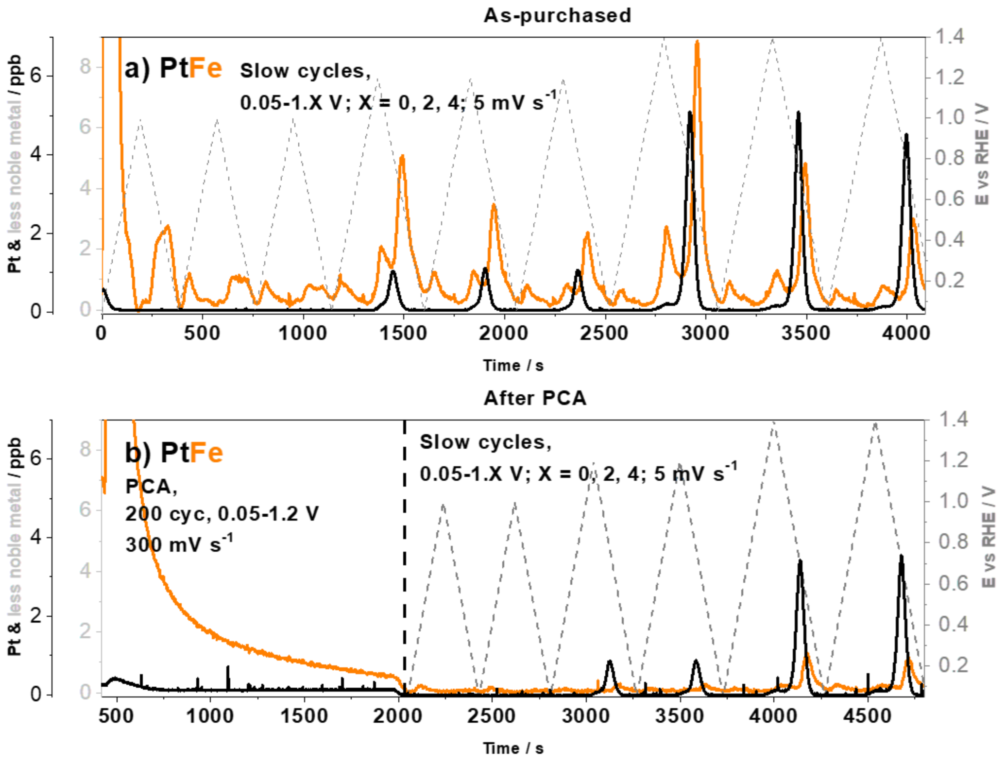

Figure S22. Comparison of EFC-ICP-MS measurements of Pt and Fe dissolution (slow cycles of 5 mV s^{-1} between 0.05–1.X V_{RHE}; X = 0, 2, 4) for Pt-Fe/C (FCS) electrocatalyst in **(a)** as-purchased state and **(b)** after PCA (200 cycles in 0.1 M $HClO_4$, 0.05–1.2 V_{RHE}, 300 mV s^{-1}).

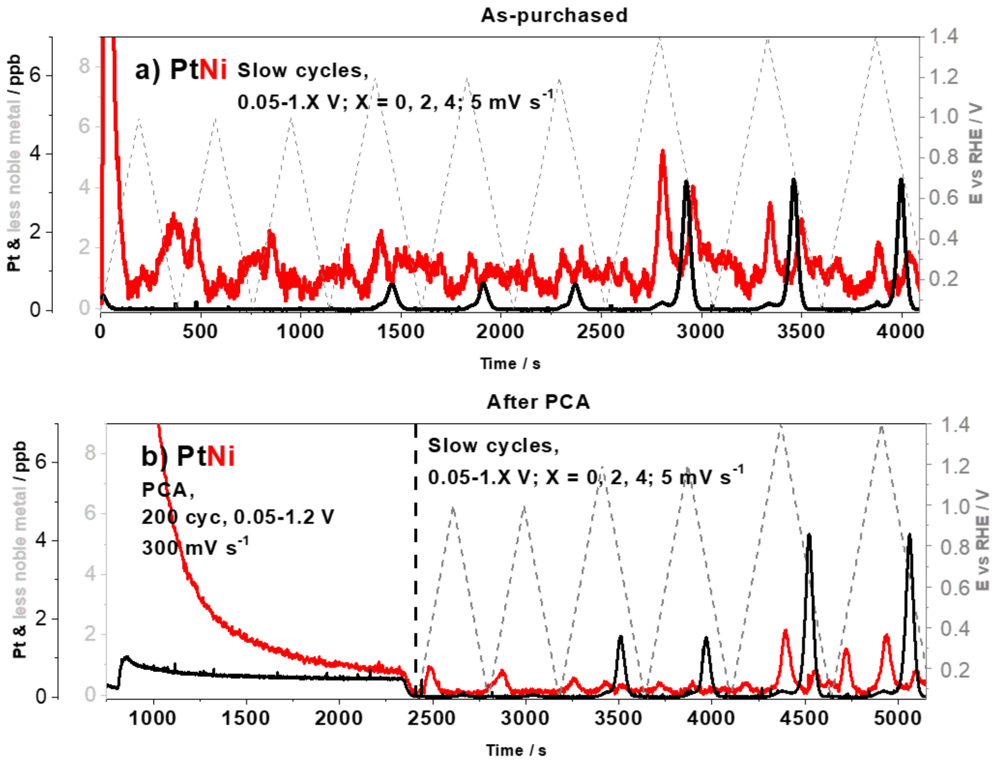

Figure S23. Comparison of EFC-ICP-MS measurements of Pt and Ni dissolution (slow cycles of 5 mV s^{-1} between 0.05–1.X V_{RHE}; X = 0, 2, 4) for Pt-Ni/C (FCS) electrocatalyst in **(a)** as-purchased state and **(b)** after PCA (200 cycles in 0.1 M $HClO_4$, 0.05–1.2 V_{RHE}, 300 mV s^{-1}).

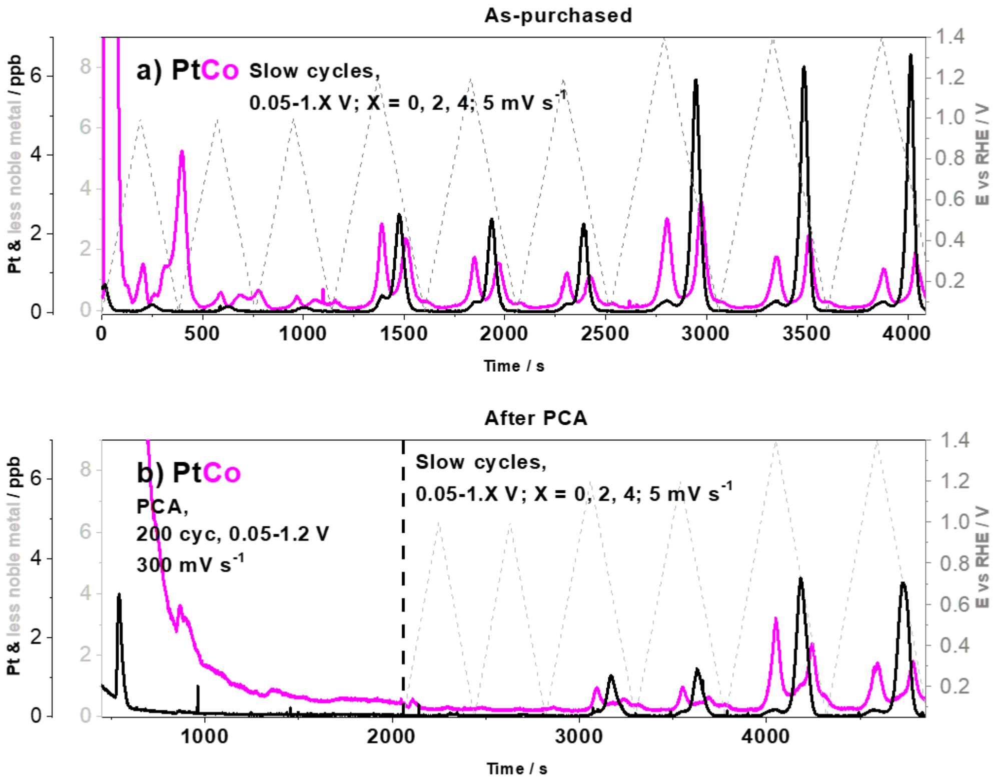

Figure S24. Comparison of EFC-ICP-MS measurements of Pt and Co dissolution (slow cycles of 5 mV s^{-1} between 0.05–1.X V_{RHE}; X = 0, 2, 4) for Pt-Co/C (FCS) electrocatalyst in **(a)** as-purchased state and **(b)** after PCA (200 cycles in 0.1 M $HClO_4$, 0.05–1.2 V_{RHE}, 300 mV s^{-1}).

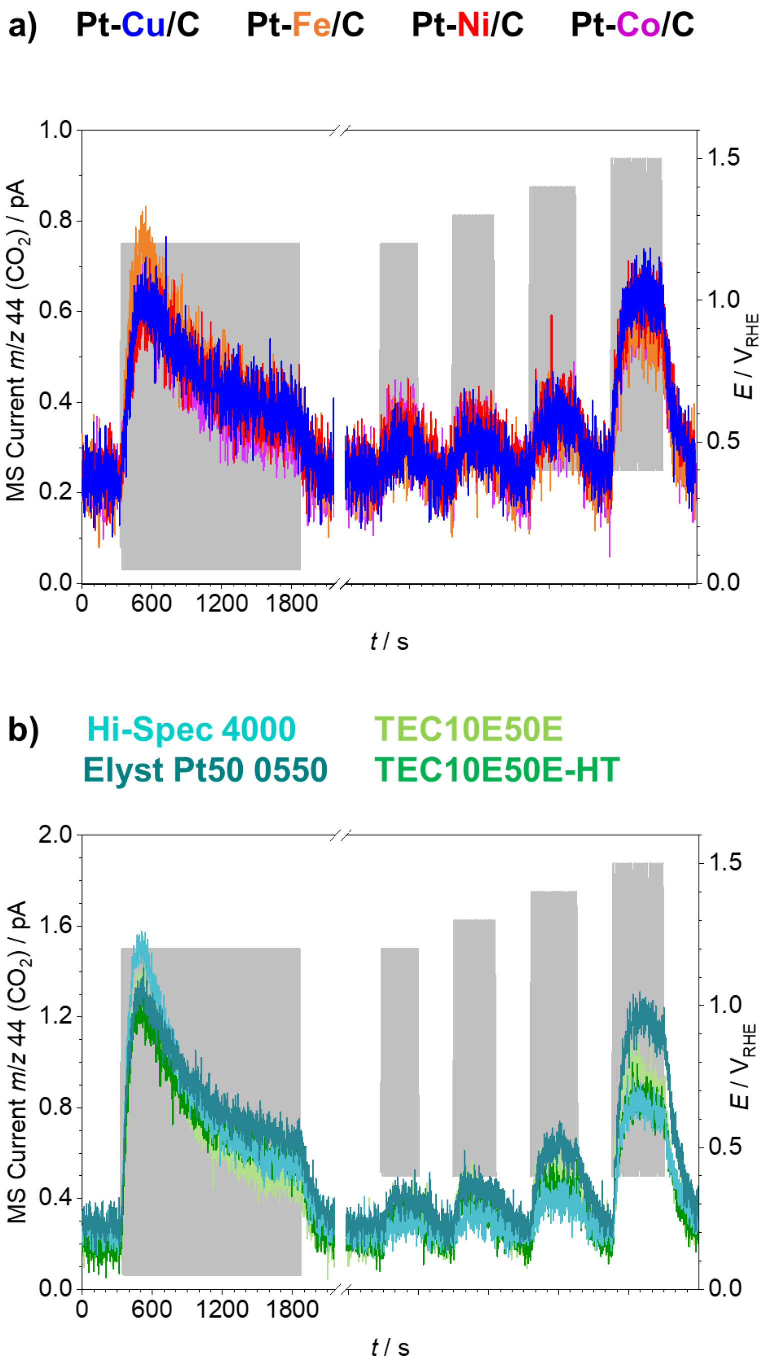

Figure S25. EC-MS measurements of carbon corrosion recorded as the MS current at *m*/*z*=44, attributed to CO_2. The electrochemical experiment was performed in 0.1 M $HClO_4$ and consisted of 100 activation cycles (0.05–1.2 V_{RHE}, 150 mV s^{-1}), followed by electrolyte exchange (not shown, x-axis break) and subsequent sets of 20 cycles with increasing UPL (0.40–1.X V_{RHE}; X = 2, 3, 4, 5; 100 mV s^{-1}), separated by 5 min of open-circuit potential. The results show degradation of **(a)** FCS Pt-M/C electrocatalysts (M = Cu, Fe, Ni, Co) and **(b)** Pt/C electrocatalysts (HI-Spec 4000, Elyst Pt50 0550, TEC10E50E, and TEC10E50E-HT).

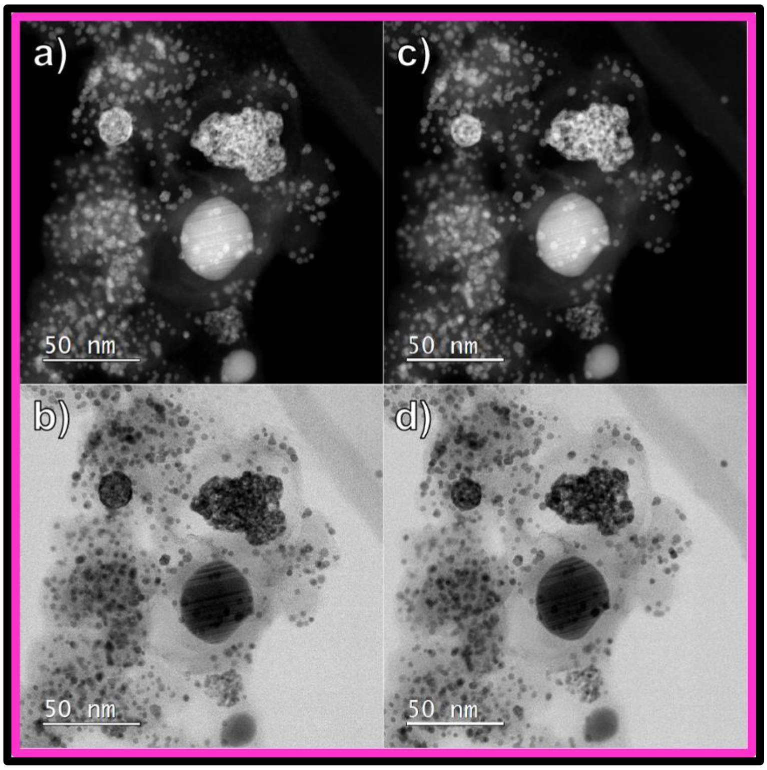

Figure S25. IL-(S)TEM comparison of Pt-Co/C FCS electrocatalyst before **(a-b)** and after **(c-d)** PCA using MFE methodology.

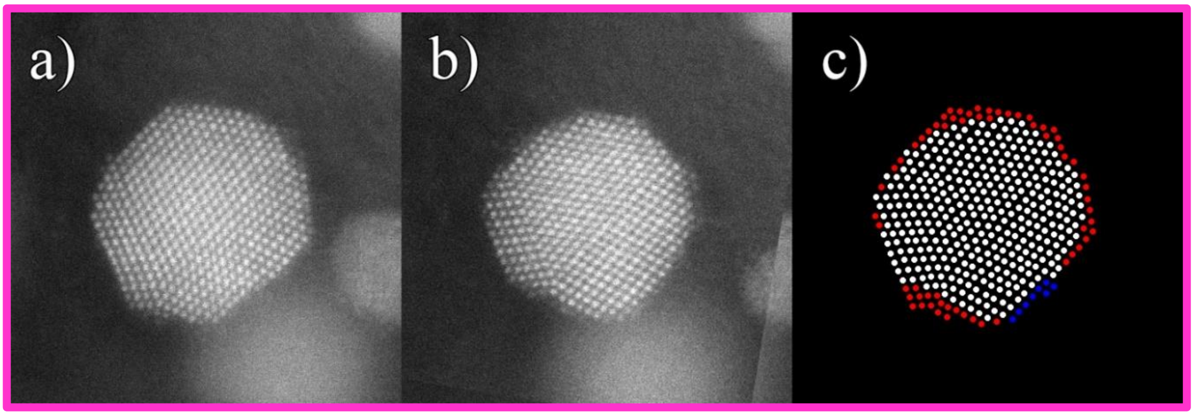

Figure S26. IL-(S)TEM comparison of a Pt-Co/C FCS electrocatalyst nanoparticle **(a)** before and **(b)** after PCA (nanoparticle number 1 from **Figure 9** of the main manuscript). Atomic resolution analysis showing evolution of atomic columns is shown in **(c)**, combining before and after images. Nanoparticle is exhibiting significant facet changes, attributed mainly to dissolution of entire facets. Images **(a)** and **(b)** were overlaid using image registration methods and subtracted one from another to reveal differences and construct **(c)**. White columns in **(c)** represent ever-present columns in the nanoparticle, red columns represent disappearing columns due to dissolution and blue columns represent appearing columns due to redeposition.

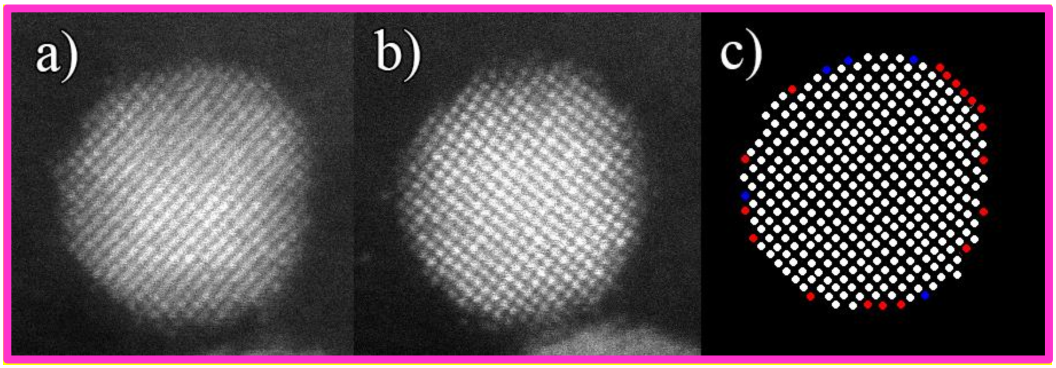

Figure S27. IL-(S)TEM comparison of a Pt-Co/C FCS electrocatalyst nanoparticle **(a)** before and **(b)** after PCA. Atomic resolution analysis showing the evolution of atomic columns is shown in **(c)**, combining before and after images. Nanoparticle is exhibiting only minor changes with individual columns disappearing or appearing and one facet uniformly dissolving.

Table S1. Commercially available Pt/C electrocatalysts from various PEMFC electrocatalyst producers (Johnson Matthey, Umicore, Tanaka Kikinzoku Kogyo).

Producer	Name	wt% (Pt)	$ECSA_{CO}$ [m^2 g_{Pt}^{-1}]	SA @0.9V [mA cm^{-2}]	MA @0.9V [A mg^{-1}_{Pt}]	Type of carbon support	Thermally annealed
Johnson Matthey	Hi-spec 4000 Pt/C	40	53.5	0.64	0.34	Vulcan XC72	No
Umicore	Elyst Pt50 0550 Pt/C	50	56.76	0.42	0.24	Ketjen Black EC300J	Yes
Tanaka Kikinzoku Kogyo	TEC10E50E Pt/C	46.3	79.7	0.6	0.48	Ketjen Black EC300J	No
Tanaka Kikinzoku Kogyo	TEC10E50E-HT Pt/C	50.6	50	0.42	0.21	Ketjen Black EC300J	Yes

Table S2. ICP-OES digestion data of Pt-M/C (M = Cu, Fe, Ni and Co) electrocatalysts.

Pt-M/C	M (wt%)	Pt (wt%)	M (at%)	Pt (at%)
**Pt-Cu/C FCS**	4.57	14.24	49.63	50.37
**Pt-Fe/C FCS**	5.06	17.84	49.77	50.23
**Pt-Ni/C FCS**	4.76	17.84	47	53
**Pt-Co/C FCS**	5.35	18.08	49.48	50.52

REFERENCES:

De Backer, A., van den Bos, K.H.W., Van den Broek, W., Sijbers, J., and Van Aert, S. (2017). StatSTEM: An efficient program for accurate and precise model-based quantification of atomic resolution electron microscopy images. J. Phys. Conf. Ser. *902*, 12013.

Hrnjić, A., Ruiz-Zepeda, F., Gaberscek, M., Bele, M., Suhadolnik, L., Hodnik, N., and Jovanovič, P. (2020). Modified Floating Electrode Apparatus for Advanced Characterization of Oxygen Reduction Reaction Electrocatalysts. J. Electrochem. Soc.

Martens, S., Asen, L., Ercolano, G., Dionigi, F., Zalitis, C., Hawkins, A., Martinez Bonastre, A., Seidl, L., Knoll, A.C., Sharman, J., et al. (2018). A comparison of rotating disc electrode, floating electrode technique and membrane electrode assembly measurements for catalyst testing. J. Power Sources *392*, 274–284.

Mayrhofer, K.J.J., Strmcnik, D., Blizanac, B.B., Stamenkovic, V., Arenz, M., and Markovic, N.M. (2008). Measurement of oxygen reduction activities via the rotating disc electrode method: From Pt model surfaces to carbon-supported high surface area catalysts. Electrochim. Acta *53*, 3181–3188.

Ophus, C., Ciston, J., and Nelson, C.T. (2016). Correcting nonlinear drift distortion of scanning probe and scanning transmission electron microscopies from image pairs with orthogonal scan directions. Ultramicroscopy *162*, 1–9.

van der Vliet, D., Strmčnik, D., Wang, C., Stamenković, V.R., Marković, N.M., and Koper, M.T.M. (2010). On the importance of correcting for the uncompensated Ohmic resistance in model experiments of the Oxygen Reduction Reaction. J. Electroanal. Chem. *647*, 29–34.

Zalitis, C.M., Kramer, D., and Kucernak, A.R. (2013). Electrocatalytic performance of fuel cell reactions at low catalyst loading and high mass transport. Phys. Chem. Chem. Phys. *15*, 4329–4340.
